# Proton-Coupled OAT Triggers C_2_H_2_ Activation in a Bioinspired Molybdenum Complex

**DOI:** 10.1021/jacs.6c08123

**Published:** 2026-08-27

**Authors:** Miljan Z. Ćorović, Lorenz Steiner, Milan R. Milovanović, Antoine Dupé, Madeleine A. Ehweiner, Michael Buchsteiner, Ferdinand Belaj, Dmytro Neshchadin, Nadia C. Mösch-Zanetti

**Affiliations:** † Institute of Chemistry, Inorganic Chemistry, University of Graz, 8010 Graz, Austria; ‡ Institute of Physical and Theoretical Chemistry, Graz University of Technology, 8010 Graz, Austria

## Abstract

Understanding acetylene coordination to group VI metals is crucial for elucidating the long-standing mechanistic ambiguities of tungstoenzyme acetylene hydratase (AH), for which several contrasting pathways have been proposed. Motivated by a recently hypothesized mechanism ( Biochemistry 2025, 64, 10, 2154–2172.40323690 10.1021/acs.biochem.5c00116PMC12096430) involving one-electron oxidation of the tungsten center, we explore the reactivity of the acetylene ligand in Mo– and W–AH models bearing 4,6-dimethylpyrimidine-2-thiolate (PymS) ligands, chosen to mimic the sulfur-rich environment of the AH active site. To trigger the redox chemistry and provide protons, half an equivalent of trimethylamine-*N*-oxide dihydrate (TMAO·2H_2_O) was added to the [MoO­(C_2_H_2_)­(PymS)_2_], yielding two distinct vinylated products: 4,6-dimethyl-1-vinylpyrimidine-2­(1H)-thione and 4,6-dimethyl-2-(vinylthio)­pyrimidine, which could be interpreted as acetaldehyde surrogates. In this transformation, TMAO serves as an oxygen atom donor, accepting electrons from Mo­(IV), and triggers the formation of the μ-oxido Mo­(V) dimer, which destabilizes the coordinated C_2_H_2_ and promotes the reaction with the neighboring ligand. The presence of a Mo­(V) species was detected by EPR spectroscopy, supporting a radical nature of the reaction. In contrast, the C_2_H_2_ ligand in the tungsten analogue does not undergo vinylation, likely due to its ligand environment not stabilizing the +V oxidation state. These observations may be extended to the biochemistry of the AH active site, where the W­(V) oxidation state would be more readily accessible. Finally, our data provide experimental evidence for potential redox events during biological acetylene hydration and additionally suggest that acetylene coordination is essential to the process.

## Graphical abstract

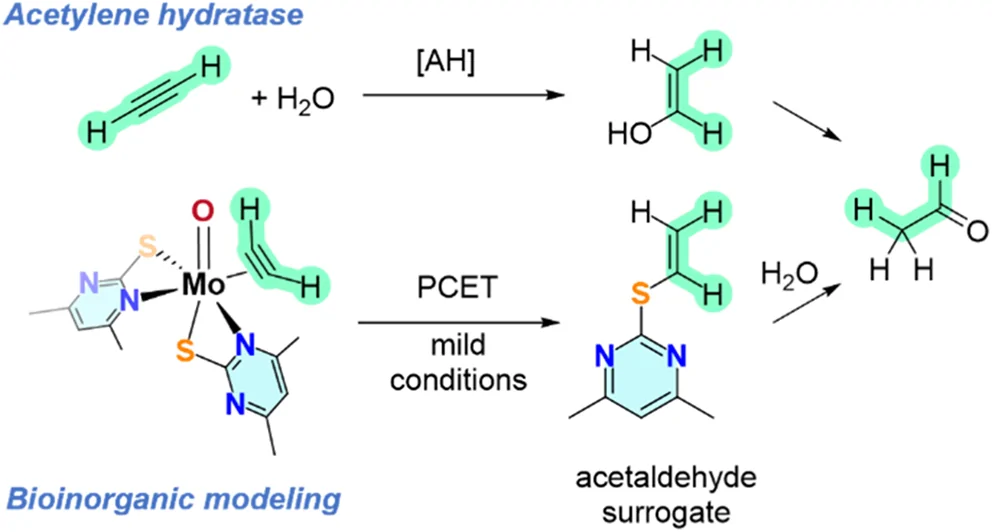

## Introduction

Studying the properties of molybdenum and tungsten complexes is crucial for understanding their biological roles, particularly the factors that allow enzymes to discriminate between the two metals.^1^ A growing number of discovered Mo and W enzymes demonstrate versatile chemistry catalyzed by pyranopterin dithiolene-stabilized active sites.^2–5^ Cells incorporate both metals into enzymes because of the redox flexibility of their high oxidation states and their capacity to support both one- and two-electron transfers, properties not available to first-row transition metals.^6^ The differences between molybdenum and tungsten biochemistry can, in part, be rationalized through relativistic effects, which influence orbital energies and metal–ligand bonding characteristics.^7^

Mo- and W-enzymes mostly operate under an oxygen atom transfer (OAT) mechanism, in which the metal cycles between high oxidation states (+IV, + V, + VI) by forming or breaking the M–O bonds.^8,9^ Notable exceptions to the typical OAT pathways include nitrogenases and acetylene hydratase, both of which have mechanisms that remain challenging to elucidate. The latter even seems to operate via a nonredox acetylene hydration at its sulfur-rich tungsten­(IV) active site, deviating from the typical mechanistic pathways of related pyranopterin dithiolene-based enzymes.^10–13^ Moreover, a molybdenum-containing variant of acetylene hydratase also catalyzed acetylene hydration, though with a 10-fold lower efficiency.^14^ Several competing interpretations of the AH mechanism have been proposed, including: (1) first-sphere acetylene coordination followed by nucleophilic attack of water,^15,16^ (2) second-sphere attack at free acetylene by coordinated water,^17^ (3) tungsten-catalyzed hydrolysis of a vinylated neighboring amino acid residue,^18^ and (4) the most recent proposal involving two coupled redox events that effectively cancel each other, making the overall reaction appear nonredox despite electron transfer occurring at the metal center.^5^ In the discussion below, mechanism (4) will be referred to as the AH redox mechanism. These mechanistic suggestions are summarized in Figure 1.

**Figure 1:**
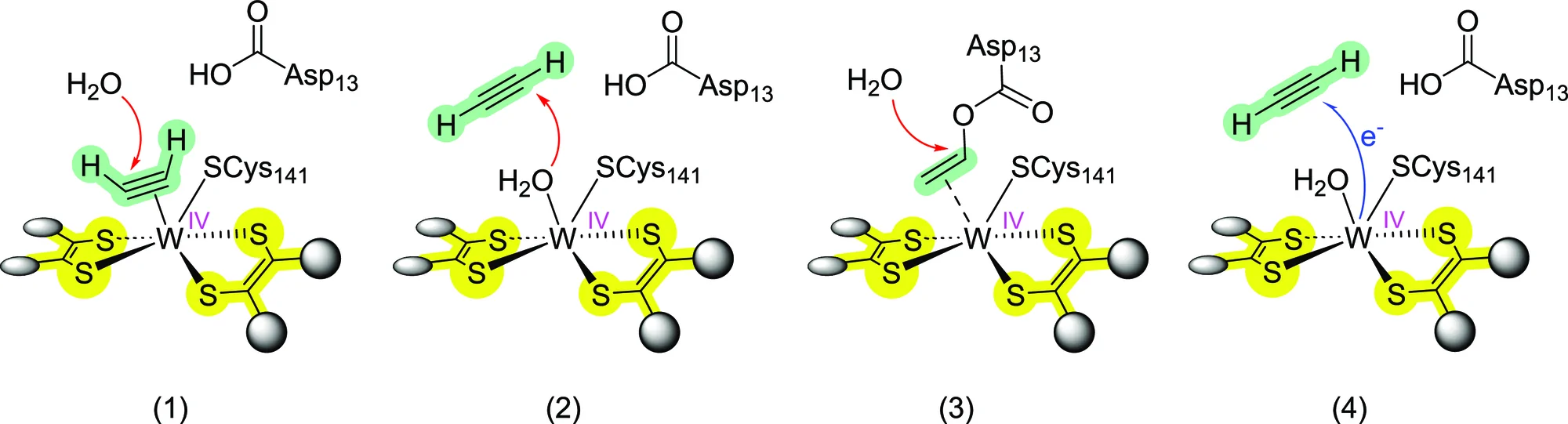
Simplified presentation of the main mechanistic proposals for acetylene hydratase: (1) first-sphere acetylene activation followed by nucleophilic attack of water;^15,16^ (2) second-sphere attack at free acetylene by coordinated water;^17^ (3) tungsten-catalyzed hydrolysis of a vinylated neighboring amino acid residue;^18^ and (4) a redox mechanism which starts by one-electron transfer from W­(IV) to free acetylene.^5^

Another notable exception to the typical oxygen atom transfer (OAT) mechanism has recently been reported for the molybdenum-containing formate dehydrogenase from *Cupriavidus necator*.^19^ Using a combination of X-ray absorption spectroscopy (including HERFD), EXAFS, and DFT calculations, the study revealed that only subtle spectroscopic changes occur upon reduction of the oxidized enzyme by formate, indicating that the redox chemistry does not primarily involve the molybdenum center. These findings suggest that, in contrast to classical molybdoenzyme mechanisms, ligand-based redox processes may play a central role.

Mimicking the active sites of some enzyme families remains a significant challenge, with AH being a notable example. Several mutually canceling mechanistic proposals, combined with the lack of functional model systems, make its modeling particularly difficult.^20^ Despite numerous attempts, coordination of unsubstituted acetylene proved infeasible at the dithiolene-coordinated tungsten compounds, which closely resemble the AH active site. Only recently, the first tungsten–alkyne adduct with a dithiolene platform was successfully isolated, featuring a disubstituted, electron-poor alkyne.^21^ For several years, we have studied tungsten-acetylene adducts supported by the bioinspired bidentate monoanionic SN ligands in the context of the first-sphere mechanism and found them generally inert,^22,23^ suggesting that simple attack at the η^2^-acetylene may not be part of the catalytic cycle of AH. However, acetylene activation and reactivity patterns were found to depend on the tungsten oxidation state, as demonstrated with complexes in both the physiological + IV and nonphysiological + II states.^24^ Furthermore, when solutions containing tungsten­(II) were subjected to an acetylene flow, N-vinylation of ancillary N-based ligands was observed in several systems.^18,25^ Upon exposure to water, the resulting N-vinyl species could generate stoichiometric amounts of acetaldehyde, supporting a mechanism involving amino acid vinylation in the enzyme and subsequent W-catalyzed hydrolysis.^18,25^

Acetylene adducts of tungsten’s lighter congener, molybdenum, are exceedingly rare, and the complex reported by our group represents the only crystallized and fully characterized Mo­(IV) η^2^-C_2_H_2_ species.^26^ In general, mononuclear Mo­(IV) oxido complexes are challenging to isolate, as they have a strong tendency to form oligomeric structures.^27^ This scarcity has so far prevented systematic comparative studies of acetylene binding in isostructural Mo and W complexes, limiting the understanding of how subtle differences between these congeners affect π-acid activation and electronic structure of the M:η^2^-C_2_H_2_ moiety.

In this work, we prepared a series of molybdenum and tungsten complexes bearing PymS ligands (4,6-dimethylpyrimidine-2-thiolate) in two oxidation states. Furthermore, we aim to test the possibility of the oxidation the Mo/W center in the high-valent acetylene complexes and, by diminishing the π-backbonding, to trigger its reactivity with nucleophiles. Thiolate ligands are chosen for their strong donor ability, which increases the electron density at S-coordinated metal centers.^28^ This elevated electron density should facilitate the oxidation of the metal centers and enhance the activation of the π-acidic acetylene ligand. Finally, the implications of these findings are discussed in the context of acetylene hydratase.

## Results and Discussion

### Synthesis and Characterization

Four new tungsten and molybdenum acetylene complexes have been prepared in two oxidation states, namely, + II (d^4^) and + IV (d^2^), following the standard procedures developed in our group.^29^ Details about the syntheses and characterization are given in the Supporting Information, and the reaction pathway is depicted in Scheme 1. After reacting the metal dihalogenido precursors with two equivalents of Na­(PymS) (4,6-dimethylpyrimidine-2-thiolate), acetylene has been purged through the solutions, thereby replacing two CO ligands in each precursor, leading to the formation of two isostructural compounds of the general structure [M­(CO)­(C_2_H_2_)­(PymS)_2_] (M = W, **W1**; M = Mo, **Mo1**) in good yields.

**Scheme 1:**
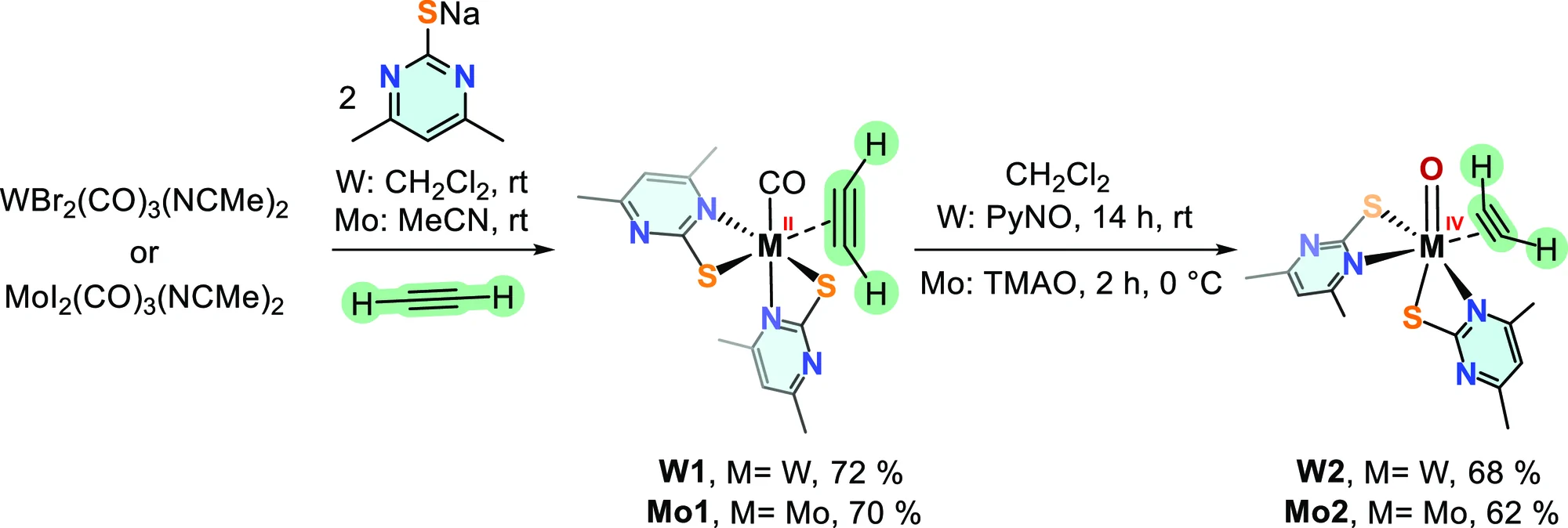
Synthesis of Tungsten and Molybdenum Compounds with an η^2^-C_2_H_2_ Ligand

The W­(II) carbonyl complex **W1** was isolated as a red-purple powder, whereas the Mo variant **Mo1** exists as a mint green powder. The compounds are air-stable in the solid state, soluble in CH_2_Cl_2_ and CHCl_3,_ and slightly soluble in THF and MeCN. NMR spectra of **W1** and **Mo1** show one isomer in CD_2_Cl_2_ solutions, asymmetric coordination of acetylene, and two sets of ancillary NS ligands. ^1^H NMR spectra in CD_2_Cl_2_ reveal low-field resonances of acetylenic protons, with the W-coordinated C_2_H_2_ shifted to a slightly lower field (13.79 and 12.47 ppm vs **Mo1** shifts of 12.52 and 12.07 ppm). ^13^C NMR shifts for both compounds are found between 200 and 206 ppm, and may therefore be interpreted as four-electron donor alkyne ligands.^30^ IR frequencies of the CO ligands are found at 1915 cm^–1^ for **W1** and 1931 cm^–1^ for **Mo1**, which corresponds to a more π-basic character of the W center. Single crystals suitable for X-ray diffraction analyses of both carbonyl acetylene compounds were obtained from saturated CH_2_Cl_2_/heptane solutions at −35 °C. The structural analysis (see the Supporting Information) reveals a parallel orientation of the η^2^-acetylene ligand to the CO moiety, as also observed for the compounds with pyridine-2-thiolate ligands.^22^ The W­(IV) oxido complex [WO­(C_2_H_2_)­(PymS)_2_] (**W2**) was synthesized according to procedures established in our group as a bright yellow microcrystalline powder in 68% yield.^23,26,31^ In this case, pyridine-*N*-oxide (Py*N*O) was used as an O-donor, and the reaction took place overnight in CH_2_Cl_2_. On the other hand, synthesis of [MoO­(C_2_H_2_)­(PymS)_2_] (**Mo2**) was not possible with Py*N*O, and the starting **Mo1** was thus reacted with a slight excess of anhydrous trimethylamine-*N*-oxide (TMAO) at 0 °C, which, after 2 h, led to isolation of a yellow powder in 62% yield. To prevent overoxidation, a low temperature must be maintained. We observed that the crude reaction mixture is extremely sensitive to moisture; even brief exposure to solvents that are not freshly dried results in the formation of a complex mixture at the cost of the desired product. However, once isolated in the solid state, **Mo2** exhibits stability with respect to both moisture and oxygen. The oxido compounds display a similar solubility to that of the respective carbonyl complexes. ^1^H NMR spectra reveal different behavior in solution for the two analogous oxido acetylene compounds at rt. The tungsten congener **W2** exhibits two well-defined isomers (regarding the orientation of the two SN ligands) in solution (7:1), with acetylene protons of the major isomer resonating at 11.25 and 10.94 ppm. The C_2_H_2_ resonances of **W2** are almost identical to those of the 6-methylpyridine-2-thiolate analogue WO­(C_2_H_2_)­(6-MePyS)_2_ (11.23 and 10.99 ppm).^31^ In contrast, **Mo2** shows fluxional behavior as evidenced by two broad peaks in the acetylenic region (Supporting Information). Variable-temperature ^1^H NMR analysis showed the splitting of the broad signals upon lowering the temperature, finally revealing the existence of the two isomers at −40 °C, presumably similar to **W2**. ^13^C NMR spectroscopy reveals a significantly lower degree of acetylene activation in the d^2^ oxido metal complexes in comparison to previously discussed d^4^ carbonyl analogues. In **W2**, acetylenic carbons resonate close to 156 ppm, while in **Mo2**, they resonate at higher field, around 137 ppm. Single crystals of **W2** and **Mo2** were obtained at −35 °C from ethyl acetate/heptane and CH_2_Cl_2_/heptane solutions, respectively. X-ray diffraction analysis revealed almost identical ligand orientation between the two isotypic structures. The metal oxido bond lies perpendicular to the metallacyclopropene moiety M:η^2^-C_2_H_2_, resembling similar oxido alkyne complexes from the literature.^22^ Molecular representations of the carbonyl and oxido forms of the Mo variants are shown in Figure 2.

**Figure 2:**
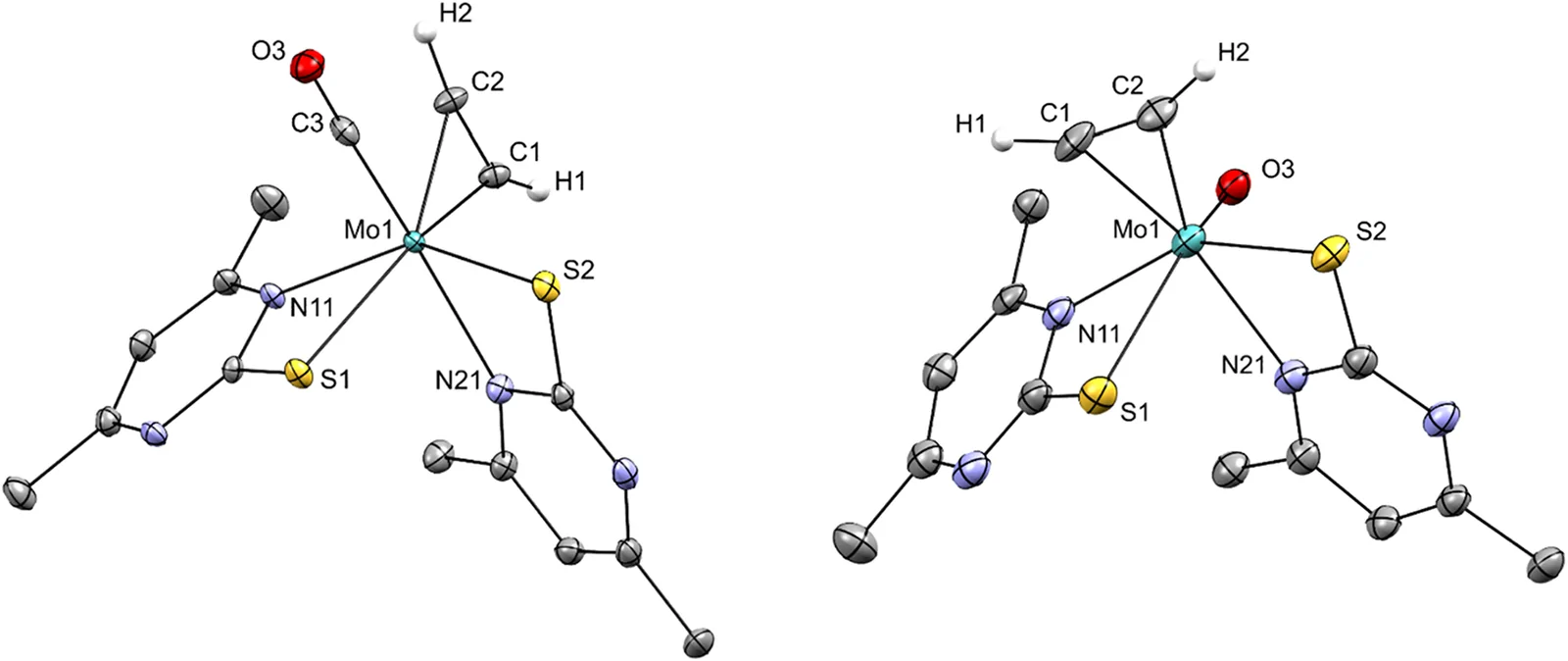
Molecular views of compounds **Mo1** and **Mo2** with labeled atoms of the first coordination sphere. The tungsten analogues are isotypic. Hydrogen atoms of the ancillary ligands are omitted for clarity.

### Nucleophilic Attack at the C_2_H_2_ Ligand

To evaluate the acetylene hydration activity of our model complexes, we introduced water to solutions of each of the four compounds. In all cases, however, no formation of acetaldehyde was detected, indicating that the coordinated acetylene ligand is not sufficiently activated for nucleophilic attack under these conditions. To address this limitation, we sought to promote such reactivity by destabilizing the acetylene ligand through oxidation of the metal center. This approach was inspired by a recent mechanistic proposal from Heider and Szaleniec,^5^ who suggested that, in acetylene hydratase, reactivity may be initiated by an outer-sphere one-electron transfer from W­(IV) to free acetylene, thereby facilitating its reaction with a neighboring aspartate residue. This process would ultimately lead to the formation of acetaldehyde and the regeneration of W­(IV). Despite this, we still consider coordination of acetylene to the biological Mo and W centers plausible (inner-sphere activation), supported by favorable orbital interactions and the plethora of W–alkyne adducts with different ligand platforms. Therefore, we proceeded to react our AH model complexes bearing coordinated C_2_H_2_ with the combination of an external electron acceptor and proton donor, to allow the natural PCET mechanism, commonly observed for group VI metalloenzymes.^3,32^ For this purpose, we have selected commercially available trimethylamine-*N*-oxide dihydrate (TMAO·2H_2_O), which can function as an electron acceptor, a proton source, and an oxygen atom donor. Notably, TMAO serves as a biological substrate for molybdoenzymes,^33,34^ and is widely utilized in synthetic chemistry as an oxidant or decarbonylation agent.^35^ To the best of our knowledge, the combination of TMAO with water to promote proton-coupled redox processes has not previously been reported in the enzyme modeling chemistry. Thus, **Mo2** and **W2** were treated with varying equivalents of TMAO·2H_2_O in CD_3_CN. The progress of the reaction was monitored via NMR spectroscopy. No matter the equivalents added, the tungsten variant **W2** undergoes a slow degradation to free C_2_H_2_ (2.17 ppm in CD_3_CN), protonated PymS ligand, corresponding disulfide (as confirmed by comparison to literature data),^36^ and insoluble precipitate (most likely W-polyoxometalate). The same was observed for **Mo2** upon the addition of the excess TMAO·2H_2_O, likely due to the rapid formation of Mo­(VI), which does not bind acetylene. However, upon addition of substoichiometric amounts of TMAO·2H_2_O to a CD_3_CN solution of **Mo2**, and 24 h of stirring at room temperature, the ^1^H NMR spectrum revealed the formation of two new organic species derived from the PymS ligand. Additionally, small amounts of free acetylene, free protonated ligand, and unreacted starting complex were detected (Figure S17). After 48 h, each of the two new organic species accounted for up to 20% yield (referenced against 1,3,5-trimethoxybenzene as an internal standard). Furthermore, HPLC–MS analysis of the NMR sample revealed two distinct peaks, each corresponding to a [M + H]^+^ ion with *m*/*z* 167. One species was identified as 4,6-dimethyl-1-vinylpyrimidine-2­(1H)-thione (**A**), as evidenced by its characteristic vinyl proton resonances at 6.71 (dd, *J* = 15.8, 8.2 Hz), 5.51 (dd, *J* = 8.2, 1.1 Hz), and 5.27 (dd, *J* = 15.8, 1.1 Hz) ppm, supported by one aromatic signal and two signals corresponding to the asymmetric methyl groups in the aliphatic region. Although this particular species has not previously been prepared, the related 6-methyl-1-vinylpyridine-2­(1*H*)-thione shows almost identical resonances in the vinyl region.^26^ The second organic species observed in the NMR spectrum also exhibited vinyl protons: 7.36 (dd, *J* = 17.5, 10.2 Hz), 5.52 (d, *J* = 17.5 Hz), and 5.46 (d, J = 10.2 Hz) ppm, along with two singlets corresponding to an aromatic proton and two symmetric methyl groups. These signals match literature values for 4,6-dimethyl-2-(vinylthio)­pyrimidine (**B**),^37^ and this compound could also be synthesized via an alternative route to further confirm its identity.^38^ The addition of excess HCl to a CD_3_CN solution of **B** and heating at 80 °C overnight led to the formation of acetaldehyde. The overall reaction is summarized in Scheme 2.

**Scheme 2:**
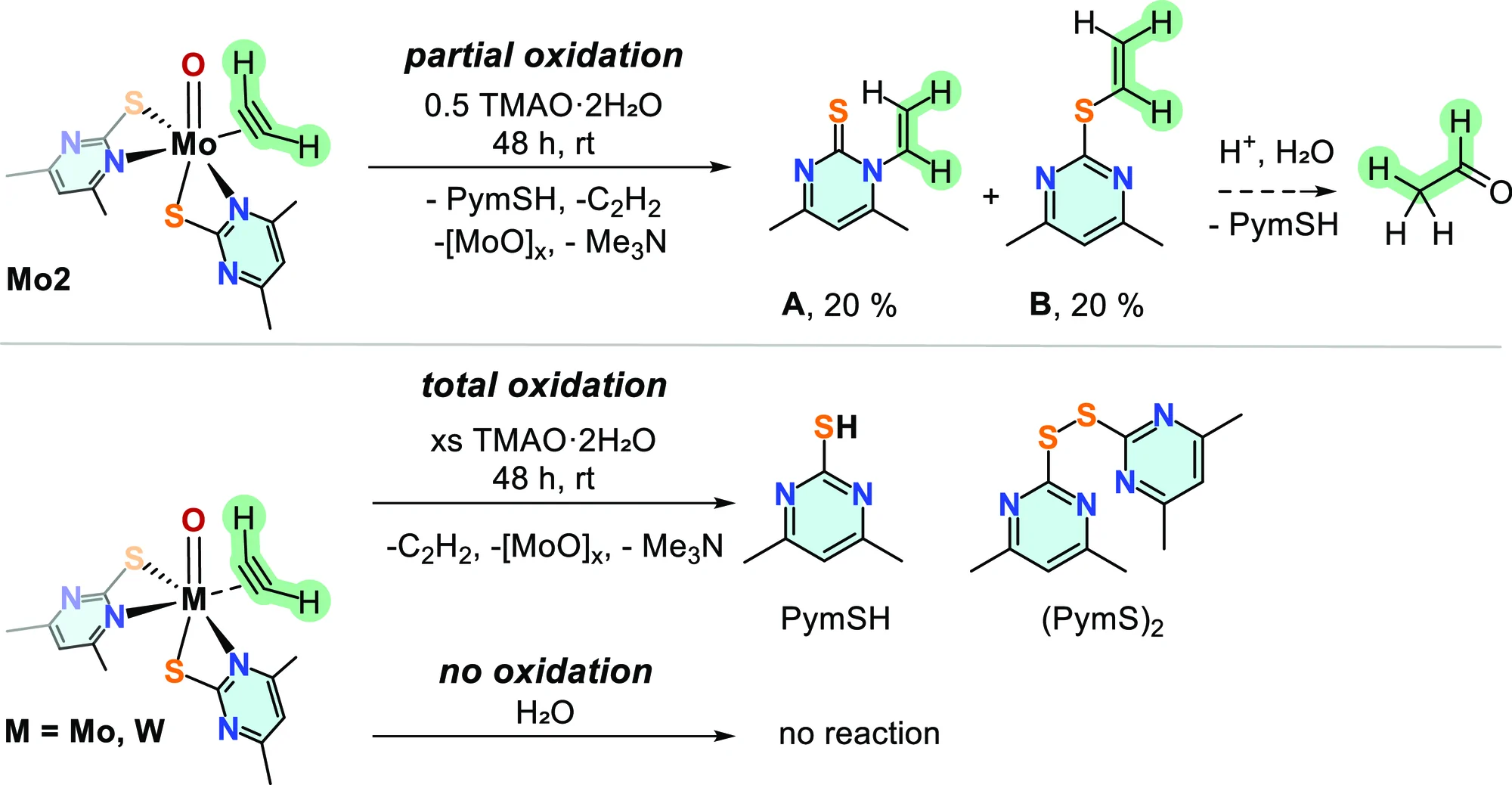
Reaction of the **Mo2** Complex with TMAO·2H_2_O Leads to Different Products Depending on the Number of Equivalents Added^a^

a Vinylation of the PymS ligand is observed only when substoichiometric amounts of TMAO are used, whereas an excess results in decomposition. Acetylene activation could not be achieved with the tungsten analogue.

To our knowledge, the formation of species **B** is the first evidence of a reaction between a chalcogen-based nucleophile and acetylene facilitated by a high-valent group VI metal. So far, only the nucleophilic attack by pnictogen-based nucleophiles was observed with pyridine-2-thiolate^22,24,26^ and pyrazole complexes,^18^ which complicated drawing direct parallels with the sulfur-rich environment of the AH active site. This unusual shift in reactivity can be attributed to an electron-poorer nature and therefore weaker nucleophilicity of aromatic nitrogen atoms in the pyrimidine- vs pyridine-2-thiolate ligands. The presence of N- and S-vinyl species is most likely a consequence of the thioketone-thioenolate tautomeric forms of the here studied PymS ligand. The easy interchange, between the two forms of the ligand was also found to be the key for activity of the closely related pyridine-2-thiolate ligands in Co-catalyzed CO_2_ electroreduction to formate.^39^ Moreover, our previous studies with analogous pyridine-2-selenolate ligands offered a closer look at chalcogene chemical environement via ^77^Se NMR and confirmed the presence of both taumeric forms in [W­(CO)­(C_2_H_2_)­(6-MePySe)_2_] (6-MePySe = 6-methylpyridine-2-selenolate).^40^ In addition to the thioketone and thioenolate forms, a thiyl radical form of the PymS ligand could be envisioned under oxidative conditions. The presence of such S-centered radicals has been detected in Ru- and Re–thiolate complexes, which could engage in reactivity with unsaturated substrates such as alkynes.^41–44^ However, our experimental data do not support such a mechanism in the Mo–C_2_H_2_ system for several reasons: The detection of both N- and S-vinyl products indicates that the reaction cannot be explained solely by the presence of S-based radicals. Moreover, treatment of a closely related pyridine-2-thiolate-based molybdenum complex with one equivalent of TMAO results in oxidation to a dioxido-molybdenum­(VI) species, which is indicative of metal-centered rather than sulfur-centered oxidation.^26^ If sulfur-centered oxidation were responsible for the reactivity, the acetylene activation with TMAO·2H_2_O would also occur with the tungsten analogue. In addition, we have not observed any sulfur-based radicals via EPR spectroscopy (see below). Nevertheless, we observed thiolate oxidation only after the metal center in the C_2_H_2_ complexes is fully oxidized. Thus, with excess oxidant present, ligand disulfide is observed as a side product.

### Mechanistic Investigations

Several control experiments were conducted to gain further insights into the reaction (summarized in Table S1). All experiments were carried out in CD_3_CN or CD_2_Cl_2_ and allowed to proceed for 48 h, after which the reaction mixtures were analyzed by ^1^H NMR spectroscopy. A detailed description is provided in the Supporting Information. To determine whether acetylene coordination to the molybdenum center is required for the vinylation, a solution of the PymS salt in CD_3_CN was treated with TMAO·2H_2_O, acetylene, and water, and monitored for 48 h. No vinylation products were detected by ^1^H NMR spectroscopy. To probe the involvement of radical intermediates, one equivalent of the galvinoxyl radical scavenger was introduced, resulting in a significant decrease in the spectroscopic yields of both **A** and **B**, suggesting the radical nature of the reaction. Moreover, to assess whether an alternative oxidant incapable of oxygen atom transfer could promote the reaction, one equivalent of the one-electron oxidant ″magic blue″ ([tris­(4-bromophenyl)­aminium hexachloroantimonate]) was added to a solution of **Mo2**, followed by addition of acetylene and water. No reaction of the acetylene ligand was observed under these conditions. This result suggests that oxygen atom transfer is essential for the process. Thus, we aimed to investigate an alternative oxygen atom transfer donor, and a newly prepared dioxido complex [MoO_2_(PymS)_2_] (for synthesis and characterization see Supporting Information) was reacted with an equimolar amount of **Mo2** in CD_2_Cl_2_ (Scheme 3). The reaction mixture was monitored via ^1^H NMR spectroscopy in CD_2_Cl_2_, due to the low solubility of [MoO_2_(PymS)_2_] and intermediate species in CD_3_CN. Although the reaction between the equimolar amounts of the dioxido complex [MoO_2_(PymS)_2_] and **Mo2** was incomplete, even after several days, imidiate color change to deep purple occurs upon mixing the solutions, indicating that at least to some extent, the comporportionation occurs.^48^ A ^1^H NMR spectrum of the reaction mixture, recorded immediately after mixing (Figure S22), reveals a partial formation of a new species characterized by broad resonances in the aromatic region. These features are consistent with those of the dimeric complex [Mo_2_O_3_(PymS)_4_], as confirmed by comparison with independently synthesized material (Figure S12). Notably, this dinuclear species crystallizes readily from reaction mixtures in acetonitrile, and its structure was unambiguously established via single-crystal X-ray diffraction (Figure S29). As observed via NMR spectroscopy, purging C_2_H_2_ through a CD_2_Cl_2_ solution of [Mo_2_O_3_(PymS)_4_] leads to the partial formation of the **Mo2**, consistent with the equilibrium suggested in Scheme 3. Furthermore, the addition of excess water (30 equiv) to an equimolar mixture of **Mo2** and [MoO_2_(PymS)_2_] in CD_2_Cl_2_ resulted in the formation of the vinyl species **A** and **B** after stirring at room temperature for 3 days (Scheme 3, Figure S22). Both species are produced in approximately 15% yield, based on comparison to the integral of the trimethoxybenzene, in line with the results of related experiments employing TMAO·2H_2_O as the oxidant. Attempts to enhance the reaction by saturating the solution with acetylene, or cooling at 0 °C, do not alter the reaction outcome.

**Scheme 3:**
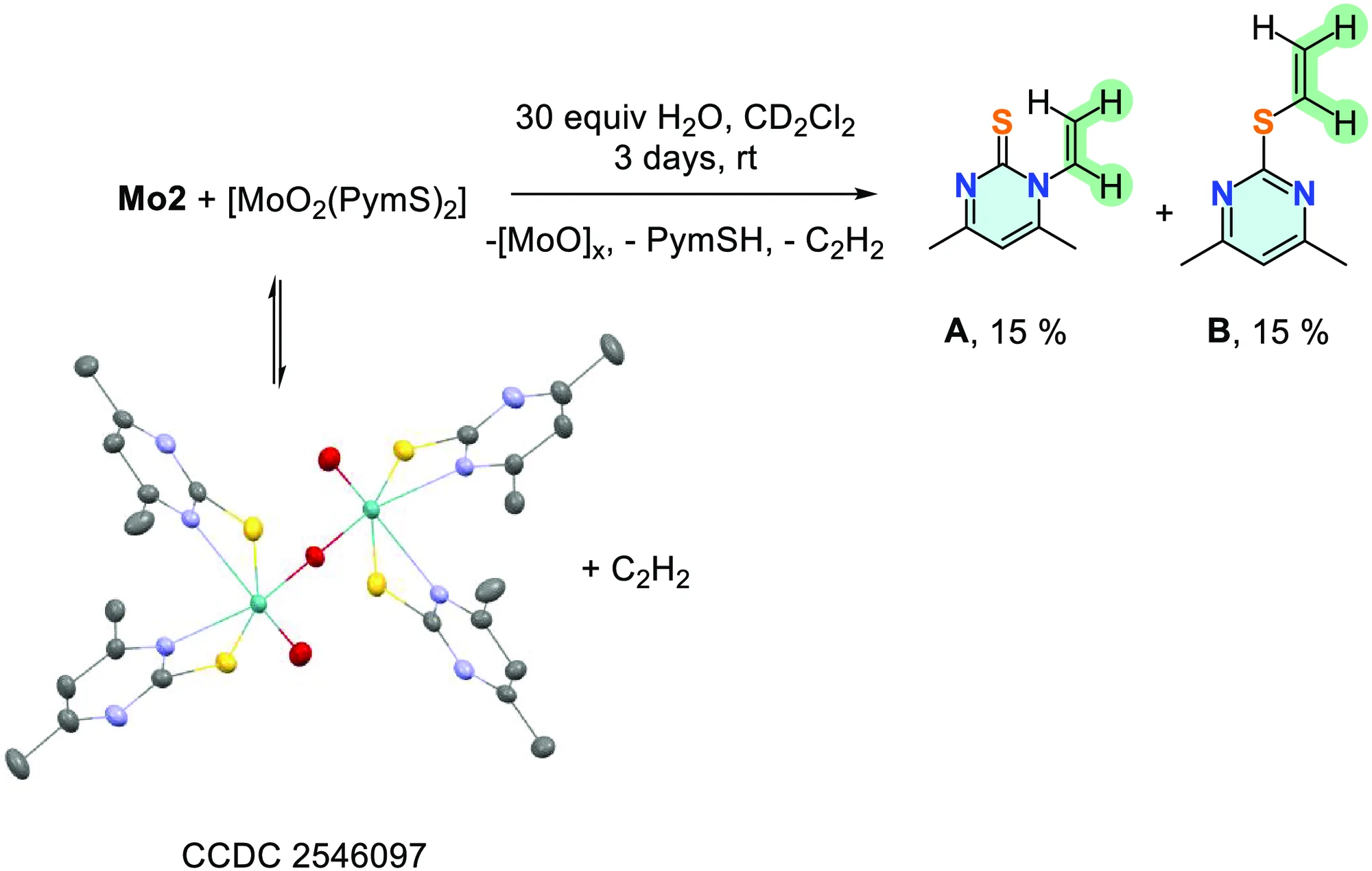
Reaction between **Mo2**, [MoO_2_(PymS)_2_], and Water in CD_2_Cl_2_ Leads to the Formation of the Vinyl Species **A** and **B** (30% vs **Mo2**)­^a^

a In addition, a molecular view of the isolated [Mo_2_O_3_(PymS)_4_] dimer is presented.

Whereas a 1:1 ratio of S- and N-vinyl species was observed in the previously described reaction with 0.5 equiv TMAO·2H_2_O or [MoO_2_(PymS)_2_], two control experiments yielded a different product distribution. As ligand consumption occurs during the oxidation of the model complex, an additional equivalent of Na­(PymS) was added to the standard reaction mixture containing **Mo2** and TMAO·2H_2_O to possibly enhance acetylene consumption. After 48 h, ^1^H NMR analysis revealed a significant increase in the overall spectroscopic yield and complete conversion of the acetylene ligand to products **A** (25%) and **B** (75%). The significantly higher yield of the S-vinyl species and almost identical yield of the N-vinyl species vs standard experiments indicates an alternative mechanism occurring after the Mo–C_2_H_2_ compound has been consumed. Similar behavior has been observed when acetylene and water have been added to an equimolar mixture of the dioxido complex [MoO_2_(PymS)_2_] and cobaltocene as a one-electron reductant. After 48 h, ^1^H NMR spectroscopy revealed the quantitative formation of the S-vinyl species vs cobaltocenium, alongside several unidentifiable decomposition products. The higher yield of the S-vinyl product is likely a consequence of the intermediate thiyl radical formation, which reacts with free acetylene in the solution.

To probe the nature of the oxidized species in situ, we investigated the reactions between **Mo2** and 0.5 equiv of TMAO·2H_2_O in CH_2_Cl_2_ via EPR spectroscopy (Figures S30 and S31). The resulting spectrum revealed only signals attributable to Mo­(V) species, with no evidence for S-centered radical intermediates. The pattern is consistent with hyperfine coupling of an S = 1/2 paramagnetic center to a nucleus with I = 5/2, as expected for ^95/97^ Mo in Mo­(V)-centered species.^49,50^ When [MoO_2_(PymS)_2_] and water were added to **Mo2** in CH_2_Cl_2_, the same pattern was observed, which did not change over 72 h during which the reactions were followed. Although the obtained signals in the EPR spectra are weak, they are consistently reproducible across both samples. Variations in line intensities and the presence of minor additional features imply that more than one Mo­(V)-type species may be contributing to the spectra. Consistent with this, attempts to simulate the experimental spectra using a model with only a single Mo­(V) species were unsuccessful. In contrast to molybdenum, EPR spectroscopy of the analogous mixture of **W2** with 0.5 equiv of TMAO·2H_2_O in CH_2_Cl_2_ showed no signals. This is consistent with tungsten μ-oxido dimers being less readily formed and the + VI oxidation state more stable.^47^ To potentially observe the oxidation state + V, we investigated the oxidation behavior of the compounds **Mo2** and **W2** via cyclic voltammetry. The CV traces for both compounds (1 mM solutions of each compound in 0.1 M nBu_4_NPF_6_/MeCN) are highly similar, each displaying a single irreversible oxidation event: at 0.98 V for **W2** and 1.03 V for **Mo2**. The number of electrons involved in the oxidation events was determined to be two in both cases by applying the Randles–Ševčík equation to cyclic voltammetry data obtained from equimolar mixtures of the complexes and ferrocene.^51^ Diffusion coefficients, required for this analysis, were independently determined by DOSY NMR spectroscopy (see Supporting Information). These features likely correspond to complete oxidation to the + VI oxidation state, accompanied by decomposition of the complex. We also attempted to observe an additional redox event in a **Mo2** CV by introducing proton sources (trifluoroacetic acid and water) to support the potential vinylation at the + V oxidation state, but no new features appeared under these conditions. This suggests that, in the absence of an oxygen atom source, the Mo­(V) species cannot be stabilized in the μ-oxido dimer form and is directly oxidized to Mo­(VI). Alternatively, the observed oxidation waves could be attributed to the formation of the ligand disulfide, which would account for the relatively similar oxidation potentials observed for both complexes.

On the basis of our experimental evidence, we propose two distinct pathways for acetylene activation in our model system, namely, a proton-coupled oxygen atom transfer (PCOAT) and a thiyl radical-based one, as illustrated in Scheme 4 and discussed below. The PCOAT mechanism requires acetylene coordination to the Mo center and predominates as long as **Mo2** remains in solution. Once the acetylene complex is fully consumed, the thiyl radical pathway may emerge.

**Scheme 4:**
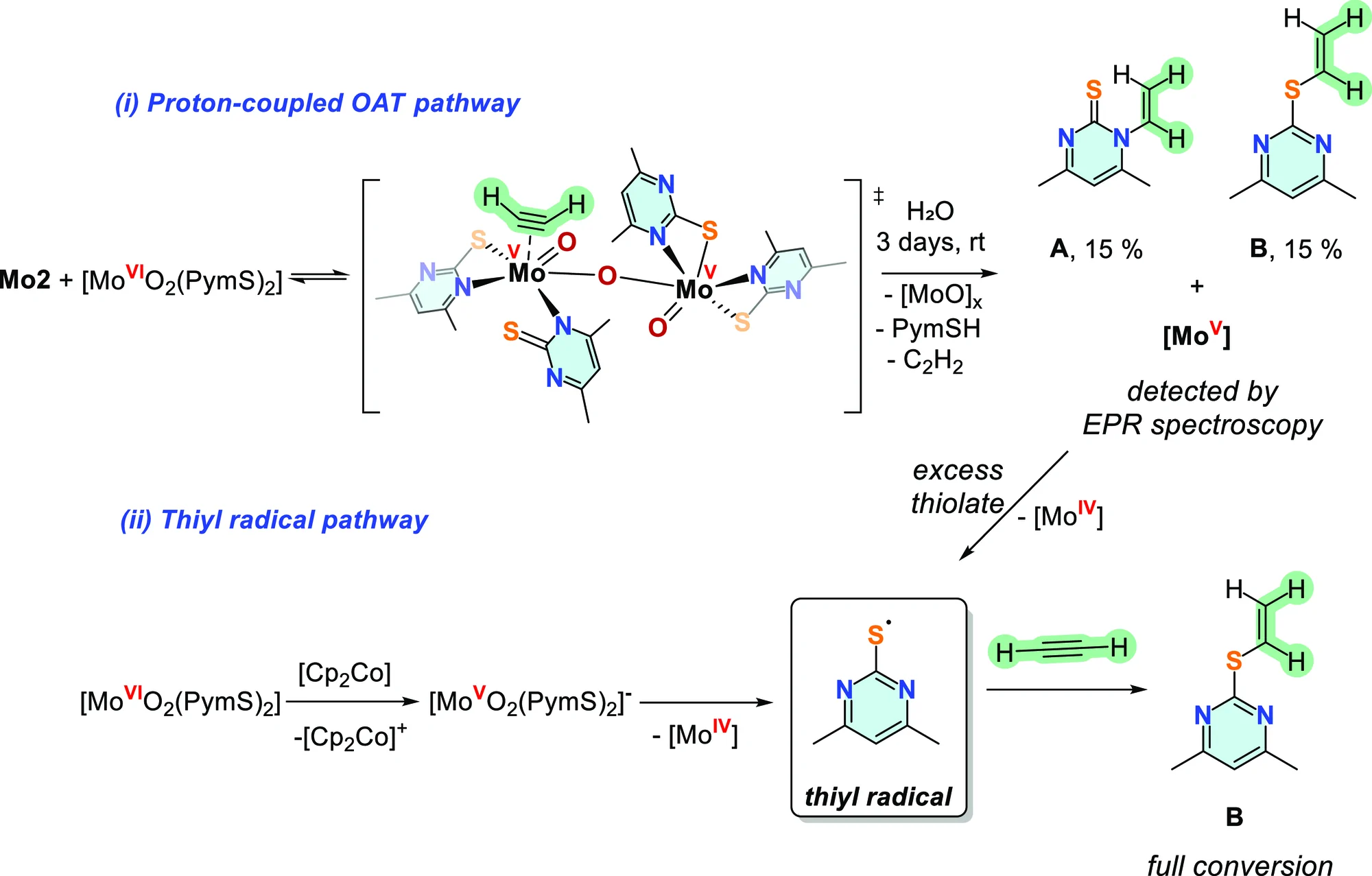
Two Distinct Mechanistic Routes for Acetylene Activation in the Model Complex **Mo2**, Based on Experimental Evidence^a^

a (i) Proton-Coupled Oxygen Atom Transfer (PCOAT), and (ii) Thiyl Radical Pathway.

The PCOAT pathway is proposed for the reaction of **Mo2** with either TMAO·2H_2_O or the dioxido complex. During the reaction of **Mo2** with TMAO·2H_2_O, [MoO_2_(PymS)_2_] is formed *in situ*, upon the two-electron oxidation of **Mo2** involving oxygen atom transfer.^46^ Given that molybdenum compounds readily form μ-oxido dimers upon comproportionation of [MoOL_n_] and [MoO_2_L_n_] species,^45^ we speculate that the Mo­(V)–C_2_H_2_ moiety may be a part of such a short-lived dinuclear species. The comproportionation (again an oxygen atom transfer process) leads to the oxidation of the metallacyclopropene moiety (Mo:η^2^-C_2_H_2_) in **Mo2** by one electron, and in the presence of water, the coordinated ligands which exist as either a thioketone (N-nucleophilic) or thioenolate (S-nucleophilic) can attack the activated C_2_H_2_ ligand. The dinuclear species would likely feature a highly destabilized acetylene ligand, which facilitates its reaction with a nearby PymS ligand, ultimately leading to vinylation. In this system, water most likely facilitates the nucleophilic attack of the PymS ligand at the coordinated C_2_H_2_ by providing protons, after the OAT has occurred. The requirement of water for acetylene activation suggests that proton transfer and acetylene activation are closely linked in the reaction pathway. However, whether these steps occur in a concerted or sequential manner (two coupled equilibria) cannot be distinguished based on the current observations. Furthermore, the reactivity of **Mo2** in the presence of both TMAO and H_2_O likely explains the high sensitivity of the crude reaction mixtures and the need for absolutely dry conditions and low temperature during the preparation. Moreover, the lack of vinyl product formation with **W2** can be attributed to the inherent resistance of high-valent tungsten complexes to undergo dimerization. This is supported by DFT calculations, which indicate that oxygen atom abstraction from tungsten dioxido complexes is energetically unfavorable, consistent with the experimental absence of μ-oxido tungsten dimers.^47^

The thiyl radical pathway is likely responsible for the predominant formation of the S-vinyl product under conditions of excess thiolate or one-electron reduction of [MoO_2_(PymS)_2_]. Notably, when excess thiolate is added to the standard **Mo2**/TMAO·2H_2_O reaction, both N-vinyl and S-vinyl products are observed, with the S-vinyl yield increasing over time and ultimately leading to complete acetylene consumption, while the N-vinyl yield remains largely unchanged. This outcome can be rationalized by considering two distinct processes: First, the PCOAT takes place, identically to what happens under standard conditions as explained in the previous paragraph. A second process most likely becomes significant only after coordinated acetylene has been consumed and a Mo­(V) species is generated. At this stage, the Mo­(V) species may be involved in a redox reaction with the ligand, accepting an electron to form Mo­(IV) and generating a thiyl radical. The latter could then react with residual acetylene in solution (liberated as half of the starting **Mo2** complex is oxidized), and subsequent quenching with water would yield the S-vinyl product. This is supported by a related experiment, in which [MoO_2_(PymS)_2_] was treated with one equivalent of [Cp_2_Co] in CD_2_Cl_2_ followed by the addition of water and acetylene, resulting in the formation of the S-vinyl species as the only product originating from C_2_H_2_. This observation suggests that, also here, the S-thiyl radical is involved, generated upon one-electron reduction of [MoO_2_(PymS)_2_] to a Mo­(V) species, which may then undergo a redox reaction with the ligand, producing Mo­(IV) and the thiyl radical. In the latter scenario, molybdenum does not play a role in activating C_2_H_2_ via coordination.

### Computational Analysis of C_2_H_2_ Complexes

All DFT calculations were performed using the ORCA program package (version 6.1).^52–61^ Starting geometries of the complexes were based on those obtained by single-crystal X-ray diffraction analysis. Geometry optimization calculations of the starting geometries of the complexes were performed using the hybrid GGA class PBE0 exchange–correlation functional^62,63^ augmented by Grimme’s fourth generation dispersion correction (i.e., PBE0-D4).^58,59,64^ All electron basis set def2-TZVP^65,66^ was used for all the atoms. Auxiliary basis sets were generated automatically.^57^ All of the geometry optimizations by energy gradient minimization were carried out with an energy gradient convergence criterion of 5 × 10^–6^ au and a tight SCF convergence criterion (1 × 10^–8^ au).

The computed DFT results align well with the experimental data (see comparison of the bond lengths from optimized geometries vs XRD data in the Supporting Information). The stronger binding in W:η^2^-C_2_H_2_ vs Mo:η^2^-C_2_H_2_ moiety can be explained by relativistic effects that contract the 6s orbital and expand the 5d orbitals, improving their energy match and overlap with the acetylene π* system.^67^ The weakest M:η^2^-C_2_H_2_ computed for **Mo2** is evidenced by the dynamic behavior in the solution at rt, further confirming that tungsten forms stronger interactions with the acetylene ligand than molybdenum. Mayer bond orders further show that the C–C bond of the acetylene ligand is strengthened upon oxidation from M­(II) to M­(IV), a consequence of reduced backbonding in the metal–oxido complexes. Analysis of atomic charges reveals that tungsten is more electropositive than molybdenum in both oxidation states examined (Table 1).

**Table 1:** Selected Löwdin Charges and Mayer Bond Orders for Tungsten and Molybdenum Acetylene Complexes

	**Löwdin charges**			**Mayer bond orders**		
**compounds**	M (W/Mo)	C1	C2	C1–C2	M-C1	M-C2
**W1**	0.075	–0.259	–0.206	1.672	1.068	0.954
**Mo1**	–0.092	–0.235	–0.189	1.714	0.984	0.940
**W2**	0.281	–0.234	–0.239	1.836	0.951	0.853
**Mo2**	0.139	–0.204	–0.208	1.995	0.750	0.879

In all cases, considerable electron density is localized at the acetylenic carbons; nevertheless, their electrophilicity increases upon replacing W with Mo. Still, in every structure, the metal center remains the most electrophilic site, which explains the commonly observed reactivity with small nucleophiles: attack at the tungsten center rather than the acetylenic carbons.^68^ Finally, the absence of the desired reactivity of the complexes **Mo1**, **W1**, **Mo2**, and **W2** with water and weak nucleophiles can be attributed to the strong and generally inert group VI metal–acetylene bonding. Comparable stability has also been reported for related tungsten compounds supported by dithiocarbamates.^69^

### Mechanistic Implications and Relevance to Acetylene Hydratase

The observed vinylation of the PymS ligand offers insights relevant to the tungstoenzyme acetylene hydratase. Both species **A** and **B** could be considered as acetaldehyde surrogates,^18^ as their hydrolysis yields acetaldehyde, the product of AH-catalyzed C_2_H_2_ hydration. We have proposed that the C_2_H_2_ ligand in **Mo2** is activated through one-electron oxidation of the molybdenum center, yielding a transient Mo­(V)–C_2_H_2_ species. This species would be characterized by diminished π-backbonding, which leads to destabilization of the coordinated acetylene ligand and increased electrophilicity. Therefore, our model experiments offer aspects which could be relevant for understanding the AH mechanism. Most likely, the active site of AH is capable of accessing the +V oxidation state, due to stabilization by the pyranopterin dithiolene ligands.^4,70^ Despite this, the precise fate of the electron removed from the starting W­(IV) center during this process remains uncertain, as there is currently no direct evidence for its transfer either to the substrate (acetylene) or to the pyranopterin dithiolene ligand. However, the latter was found not just to be a passive spectator, but is electronically active and can participate in redox chemistry alongside the metal center.^71^ Thus, the single electron transfer from the metal center, coupled by proton transfer from a nearby water, likely triggers acetylene activation for the nucleophilic attack either by a neighboring nucleophile, water, or a nearby amino acid residue in the active site. Based on experimental evidence with our model complex, we propose a mechanism that involves acetylene coordination, as shown in Scheme 5. In the AH active site, once acetylene is coordinated, a one-electron oxidation of the W center couples with a proton transfer (PCET) which triggers the acetylene activation by a neighboring nucleophile (Asp13, Cys141, or water/hydroxide). The W­(V) center and the radical formed (an organic intermediate or the pyranopterin dithiolene) undergo another PCET to recover the active site and release the acetaldehyde. To better convey our idea, we have chosen to present the reaction with the aspartate residue, based on the geometry of the active site and to localize the radical on the pyranopterin dithiolene ligand; however, we emphasize that this assignment remains speculative.

**Scheme 5:**
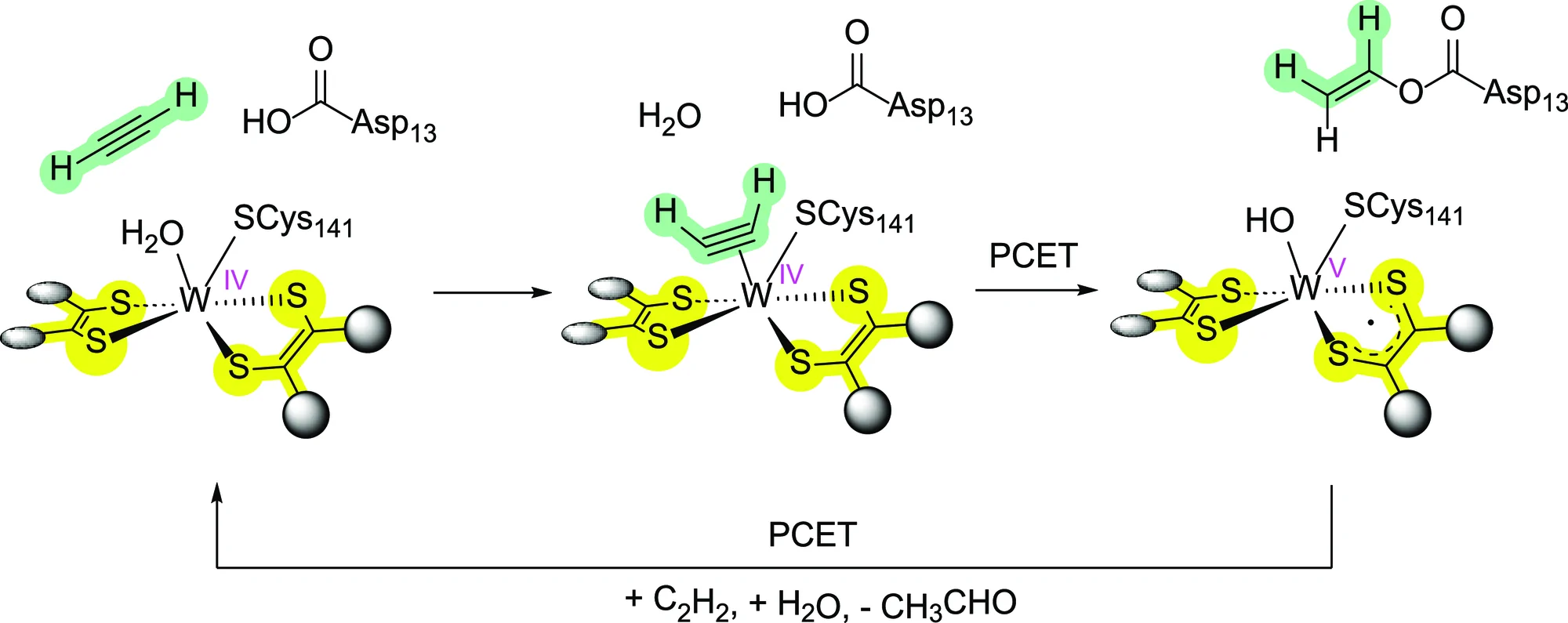
Proposed Mechanistic Scenario for AH^a^

a The mechanism involves acetylene coordination and activation via proton-coupled electron transfer (PCET), forming W­(V). The active site is regenerated by acetaldehyde release and recovery of the W­(IV) center again via PCET. For clarity, the two pyranopterin dithiolene cofactors are depicted in a simplified manner, showing only the first coordination sphere of the biological cofactor, as well as the neighboring Asp13 and Cys141 residues.

In addition, we observed that in situ-generated sulfur-centered radicals can interact with C_2_H_2_. As a result, an alternative S-centered redox pathway cannot be fully excluded, and X-ray absorption spectroscopic studies of the enzyme would be necessary to clarify this. Overall, our findings suggest that redox chemistry could be involved in the AH mechanism, and we challenge the recently proposed redox mechanism^5^ by providing evidence that acetylene coordination may still occur.

## Conclusion

Our investigations of the reactivity of acetylene ligand at bioinspired molybdenum and tungsten centers reveal that [MoO­(C_2_H_2_)­(PymS)_2_] undergoes both S- and N-vinylation of the ancillary pyrimidine-2-thiolate ligand upon treatment with an O-atom donor and protons. The transformation is enabled by the ligand’s ability to access both thioketone and thioenolate tautomers. Particularly noteworthy is the observed S-vinylation, which potentially occurs in the sulfur-rich environment of the AH active site. This supports the hypothesis that enzymatic acetylene activation may involve direct functionalization by neighboring groups to generate vinyl intermediates prior to hydrolysis to acetaldehyde. Furthermore, our results highlight the crucial role of ligand design in bioinorganic AH modeling, especially in supporting the metals’ +IV and +V oxidation states. With our PymS ligand system, both states could be achieved with molybdenum, allowing coordination at the lower and activation at the higher oxidation state. In contrast, the tungsten analogue bypasses the W­(V) state and is directly oxidized to W­(VI), which does not activate the acetylene ligand. Taken together, our findings challenge the prevailing view of a redox-free mechanism in acetylene hydratase and provide experimental support for the plausibility of redox-mediated pathways.

## Supplementary Material



## Data Availability

The data supporting this article, including NMR files (.csv), IR spectra (.dpt), and coordinates of the optimized geometries (.xyz), are available on Zenodo at 10.5281/zenodo.17223511.

## Notes

Full experimental procedures, NMR spectra and computational data with optimized geometries (PDF)
