# Supplementary material for: Proton-Coupled OAT Triggers C2H2 Activation in a Bioinspired Molybdenum Complex
Source: J Am Chem Soc. 2026 Aug 27;148(35):37841–51. doi: 10.1021/jacs.6c08123 (PMC13564428; doi:10.1021/jacs.6c08123)
Supplement: Supplementary file 1 [file ja6c08123_si_001.pdf]

Supporting Information

Proton-coupled OAT Triggers C_2H_2 Activation in a Bioinspired Molybdenum Complex

Miljan Z. Ćorović,[a] Lorenz Steiner,[a] Milan R. Milovanović,[a] Antoine Dupé,[a] Madeleine A. Ehweiner,[a] Michael Buchsteiner,[a] Ferdinand Belaj,[a] Dmytro Neshchadin,[b] Nadia C. Mösch-Zanetti,[a]*

[a]*Institute of Chemistry, Inorganic Chemistry, University of Graz, 8010 Graz, Austria*

[b]*Institute of Physical and Theoretical Chemistry, Graz University of Technology, 8010 Graz, Austria*

*Corresponding author email: nadia.moesch@uni-graz.at

Contents

1 General Considerations 2

2 Synthetic Procedures 4

3 NMR Spectra 7

4 Reactivity Studies 22

5 X-Ray Crystallographic Data 35

6 Electron Paramagnetic Resonance 46

7 Cyclic Voltammetry 49

8 Computational Data 53

References 56

1 General Considerations

All synthetic manipulations and NMR experiments were performed under a nitrogen atmosphere using standard Schlenk and glovebox techniques. Solvents were purified via a Pure Solv Solvent Purification System. Chemicals were purchased from commercial sources and apart from acetylene, sodium hydride, and pyridine *N*-oxide, all were used without further purification. Acetylene 2.6 was purified by bubbling it through water and conc. H_2SO_4 and subsequently dried by passing it through $CaCl_2$ and KOH. Pyridine *N*-oxide was purified by recrystallization from Et_2O and subsequent sublimation. Trimethylamine *N*-oxide was purified by sublimation. NaH 60% in mineral oil was washed with pentane to give pure NaH. Celite was dried at 100 °C prior to use, and silica gel was washed with Et_3N and subsequently dried in vacuo under heating. The metal precursor complexes $[WBr_2(CO)_3(NCMe)_2]$,[1] and $[MoI_2(CO)_3(NCMe)_2]$,[2] and the ligand PymSH[3] were synthesized according to established procedures. The ligand was deprotonated as previously reported for similar systems with NaH in THF.[4] 4,6-Dimethyl-2-(vinylthio)pyrimidine has been prepared according to the literature procedure.[5]

Standard NMR spectra were recorded using a Bruker Avance III 300 MHz spectrometer. Chemical shifts δ are given in ppm. Diffusion-ordered spectroscopy (DOSY) experiments were performed on a Bruker Avance III spectrometer (Ascent 700 MHz magnet) equipped with a triple resonance 5 mm cryoprobe featuring ATMA and Z-gradient capabilities. ^{1}H NMR spectra are referenced to residual protons in the solvent and ^{13}C NMR spectra to the deuterated solvent peak. The multiplicity of peaks is denoted as singlet (s), doublet (d), triplet (t), quadruplet (q), doublet of doublets (dd), doublet of doublets of doublets (ddd), doublet of triplets (dt), triplet of doublets (td), or multiplet (m). NMR solvents were stored over molecular sieves. NMR experiments were performed in J. Young NMR tubes. Solid state IR spectra were measured on a Bruker ALPHA ATR-FT-IR spectrometer at a resolution of 2 cm^{-1}. The relative intensity of signals is declared as strong (s), medium (m), weak (w), and very weak (vw). Elemental analyses (C, H, N, S) were performed at the Department of Inorganic Chemistry at the University of Technology in Graz. Values for elemental analyses are given as percentages. UV–Vis spectra were acquired with a Varian Cary 50 spectrophotometer equipped with a VWR thermostat.

Electrochemical measurements were performed using a Gamry Instruments Reference 600 potentiostat. A conventional three-electrode setup was employed, consisting of a platinum disk

working electrode (3 mm diameter, 7 mm OD PEEK body), a silver/silver nitrate ($Ag/AgNO_3$) reference electrode in MeCN, and a platinum wire counter electrode (0.4 mm diameter, 15 cm length). The supporting electrolyte was 0.1 M tetraethylammonium hexafluorophosphate (Et_4NPF_6) in dry acetonitrile. Cyclic voltammograms were recorded inside a glovebox at a scan rate of 100 mV s^{-1}. All potentials were referenced and are reported versus the Fc^+/Fc redox couple.

2 Synthetic Procedures

$[W(CO)(C_2H_2)(PymS)_2]$ (**W1**): Na(PymS) (1.01 g, 6.21 mmol) was added portionwise to a stirred solution of $[WBr_2(CO)_3(NCMe)_2]$ (1.53 g, 3.00 mmol) in 40 mL of CH_2Cl_2. After 40 min, the resulting suspension was purged with acetylene for 20 min and then allowed to stir under an acetylene atmosphere for 1 h. Thereafter, all volatiles were removed in vacuo. The resulting light purple-brown solid was suspended in 100 mL of CH_2Cl_2, and this suspension was then filtered through a pad of Celite/silica gel 1:1. The volume of the filtrate was reduced to 40 mL, whereupon 20 mL of heptane was added. After slow evaporation to approx. 15 mL, purple crystals were isolated by filtration, washed with pentane (3x 7 mL) and dried in vacuo to give pure $[W(CO)(C_2H_2)(PymS)_2]$ (1.11 g, 72%). ^{1}H NMR (CD_2Cl_2, 300 MHz): δ 13.79 (s, 1H, C≡CH), 12.47 (s, 1H, C≡CH), 6.88 (s, 1H, pymH), 6.56 (s, 1H, pymH), 2.44 (s, 3H, CH_3), 2.36 (s, 3H, CH_3), 1.93 (s, 3H, CH_3), 1.21 (s, 3H, CH_3). ^{13}C NMR (75 MHz, CD_2Cl_2): δ 234.3 (CO), 206.0 (C≡C), 203.7 (C≡C), 184.9 (CS), 176.3 (CS), 170.2 (CCH_3), 167.7 (CCH_3), 165.6 (CCH_3), 162.3 (CCH_3), 117.7 (pymCH), 116.3 (pymCH), 26.0 (CH_3), 24.8 (CH_3), 24.1 (CH_3), 22.1 (CH_3). IR (cm^{-1}): 1915 (s, C≡O). EI-MS (70 eV) *m/z*: $[M - CO]^+$ 488.0, $[M - CO - C_2H_2]^+$ 462.0. Anal. Calcd for $C_{15}H_{16}N_4OS_2W$: C, 34.90; H, 3.12; N, 10.85; S, 12.42. Found: C, 34.85; H, 3.04; N, 10.94; S, 12.25.

$[WO(C_2H_2)(PymS)_2]$ (**W2**): A solution of $[W(CO)(C_2H_2)(PymS)_2]$ (1.82 g, 3.52 mmol) and pyridine *N*-oxide (351 mg, 3.70 mmol) in 60 mL MeCN was stirred in a flask equipped with a bubbler for 14 h. After evaporation to dryness, the yellow solid was washed with Et_2O (5x 5 mL) using an ultrasonic bath and dried in vacuo to yield $[WO(C_2H_2)(PymS)_2]$ (1.20 g, 68%) as bright yellow powder. ^{1}H NMR (300 MHz, CD_2Cl_2, major isomer): δ 11.25 (s(d), $^2J_{WH}$ = 10.3 Hz, 1H, C≡CH), 10.94 (s(d), $^2J_{WH}$ = 11.7 Hz, 1H, C≡CH), 6.98 (s, 1H, pymH), 6.90 (s, 1H, pymH), 2.56 (s, 3H, CH_3), 2.47 (s, 3H, CH_3), 2.44 (s, 3H, CH_3), 2.05 (s, 3H, CH_3) ppm. ^{13}C NMR (75 MHz, CD_2Cl_2): δ 181.4 (CS), 179.1 (CS), 170.9 (CCH_3), 170.1 (CCH_3), 166.0 (CCH_3), 163.0 (CCH_3), 158.2 (C≡C), 157.0 (C≡C), 118.6 (pymCH), 116.8 (pymCH), 24.9 (CH_3), 24.8 (CH_3), 24.7 (CH_3), 21.2 (CH_3) ppm. IR (cm^{-1}): 933 (s, W=O). EI-MS (70 eV) *m/z*: $[M - C_2H_2]^+$ 478.0. Anal. Calcd for $C_{14}H_{16}N_4OS_2W$: C, 33.35; H, 3.20; N, 11.11; S, 12.72. Found: C, 33.55; H, 3.11; N, 11.15; S, 13.05.

$[Mo(CO)(C_2H_2)(PymS)_2]$ (**Mo1**): Na(PymS) (1.02 g, 6.30 mmol) was added portionwise to a stirred solution of $[MoI_2(CO)_3(NCMe)_2]$ (1.55 g, 3.00 mmol) in 35 mL MeCN. After 45 min, the resulting suspension was purged with acetylene for 15 min and was then allowed to stir

under an acetylene atmosphere for 16 h at rt. Thereafter, all volatiles were removed in vacuo. The resulting green-brown solid was suspended in 40 mL CH_2Cl_2, and this suspension was then filtered through silica gel. The volume of the filtrate was reduced to 8 mL, whereupon 6 mL heptane were added. The resulting precipitate was isolated by filtration, then washed twice with a mixture of 4 mL CH_2Cl_2 and 3 mL heptane and dried in vacuo to give [Mo(CO)(C_2H_2)(PymS)$_2$] (900 mg, 70%) as mint green powder. ^{1}H NMR (CD_2Cl_2, 300 MHz): δ 12.52 (s, 1H, C≡CH), 12.07 (s, 1H, C≡CH), 6.78 (s, 1H, pymH), 6.45 (s, 1H, pymH), 2.42 (s, 3H, CH_3), 2.28 (s, 3H, CH_3), 1.94 (s, 3H, CH_3), 1.29 (s, 3H, CH_3) ppm. ^{13}C NMR (CD_2Cl_2, 75 MHz): δ 236.3 (CO), 202.1 (C≡C), 200.5 (C≡C), 181.6 (CS), 177.7 (CS), 170.3 (CCH_3), 168.0 (CCH_3), 165.8 (CCH_3), 164.0 (CCH_3), 117.1 (pymCH), 115.8 (pymCH), 25.6 (CH_3), 24.5 (CH_3), 24.0 (CH_3), 22.0 (CH_3) ppm. IR (cm^{-1}): 1931 (s, C≡O). EI-MS (70 eV) *m/z*: $[M – CO]^+$ 402.0, $[M – CO – C_2H_2]^+$ 376.0. Anal. Calcd. For $C_{15}H_{16}N_4OS_2Mo$: C, 42.06; H, 3.76; N, 13.08; S, 14.97. Found: C, 41.61; H, 3.54; N, 12.60; S, 14.68.

[MoO(C_2H_2)(PymS)$_2$] (**Mo2**): A solution of [Mo(CO)(C_2H_2)(PymS)$_2$] (428 mg, 1.00 mmol) and trimethylamine-*N*-oxide (94 mg, 1.25 mmol) in 24 mL of CH_2Cl_2 was stirred for 2 h at 0 °C. After evaporation of all volatiles, 60 mL of CH_2Cl_2 was added to the solid, and the resulting suspension was filtered through silica gel giving a lemon yellow filtrate. The volume of the filtrate was then reduced to 10 mL, and 15 mL of heptane was added. Evaporation to approx. 3 mL gave a brown oil covered with light yellow solid which was then isolated by cannulation. The sticky solid was washed with 5 mL of pentane, dried in vacuo, ground with a spatula, again washed with 5 mL of pentane and dried in vacuo to give [MoO(C_2H_2)(PymS)$_2$] (260 mg, 62%) as yellow powder. ^{1}H NMR (CD_2Cl_2, 300 MHz, –40 °C, major isomer - A): δ 9.30 (s, 1H, C≡CH), 8.80 (s, 1H, C≡CH), 6.88 (s, 1H, pymH), 6.80 (s, 1H, pymH), 2.53 (s, 3H, CH_3), 2.41 (s, 3H, CH_3), 2.37 (s, 3H, CH_3), 2.05 (s, 3H, CH_3) ppm. ^{13}C NMR (CD_2Cl_2, 75 MHz, –40 °C, major isomer - A): δ 180.7 (CS), 178.8 (CS), 170.3 (CCH_3), 169.2 (CCH_3), 166.4 (CCH_3), 163.2 (CCH_3), 138.5 (C≡CH), 136.5 (C≡CH), 117.5 (pymCH), 116.1 (pymCH), 31.8 (CH_3), 22.7 (CH_3), 20.9 (CH_3), 14.1 (CH_3) ppm. IR (cm^{-1}): 928 (Mo=O). EI-MS (70 eV) *m/z*: $[M – C_2H_2]^+$ 362.0. Anal. Calcd. For $C_{14}H_{16}N_4OS_2Mo \cdot 0.1\ CH_2Cl_2 \cdot 0.1\ C_7H_{16}$: C, 40.87; H, 4.12; N, 12.88; S, 14.74. Found: C, 41.13; H, 4.04; N, 12.75; S, 14.39.

[MoO_2(PymS)$_2$]: The compound has been prepared from [MoO_2Cl_2(DMF)$_2$] (250 mg, 0.72 mmol) and the ligand salt Na(PymS) (240 mg, 1.48 mmol) in MeCN, following the literature procedure,[6] and isolated from a CH_2Cl_2/heptane mixture as ocher crystalline solid (190 mg, 65%). ^{1}H NMR (300 MHz, CD_2Cl_2) δ 6.76 (s, 2H, ArH), 2.62 (s, 6H, Me), 2.40 (s, 6H, Me)

ppm. ^{13}C NMR (75 MHz, CD_2Cl_2) δ 174.9 (qC), 171.2 (qC), 166.6 (qC), 118.1 (Ar**C**H), 24.5 (Me), 24.0 (Me) ppm. IR (Mo=O, cm^{-1}): 897, 929. EI-MS (70 eV) m/z: $[M]^+$ 408.1 Anal. Calcd. For $C_{12}H_{14}N_4O_2S_2Mo \cdot 1\ CH_2Cl_2$: C, 31.78; H, 3.28; N, 11.40; S, 13.05. Found: C, 32.02 ; H, 3.18; N, 11.32; S,12.66.

$[Mo_2O_3(PymS)_4]$: $[MoO_2(PymS)_2]$ (80 mg, 0.196 mmol) was suspended in 10 mL of MeCN, following the addition of PPh_3 (31 mg, 0.118 mmol), and stirring for 2 h at room temperature. The suspension was reduced to 3 mL, 5 mL of diethyl ether was added to precipitate the product. The violet residue was washed with 5 mL of diethyl ether two times and dried to yield 82 mg (52 %) of $[Mo_2O_3(PymS)_4]$. ^1H NMR (300 MHz, CD_2Cl_2, rt) broad signals at 6.76 (ArH), 6.73 (ArH), 6.49 (ArH), 2.66 (Me), 2.44 (Me) ppm and 2.34 (Me) ppm. ^{13}C NMR (CD_2Cl_2, 75 MHz): 117.0 (Ar**C**H), 115.6 (Ar**C**H), 24.8 (Me), 24.3 (Me) ppm. IR (Mo=O, cm^{-1}): 947. EI-MS (70 eV) m/z: $[MoO(PymS)_2]^+$ 392.0; $[MoO_2(PymS)_2]^+$ 408.0. Anal. Calcd. For $C_{24}H_{28}N_8O_3S_4Mo_2$: C, 36.18; H, 3.54; N, 14.07; S, 16.10. Found: C, 36.39 ; H, 3.00; N, 13.96; S, 15.50.

3 NMR Spectra

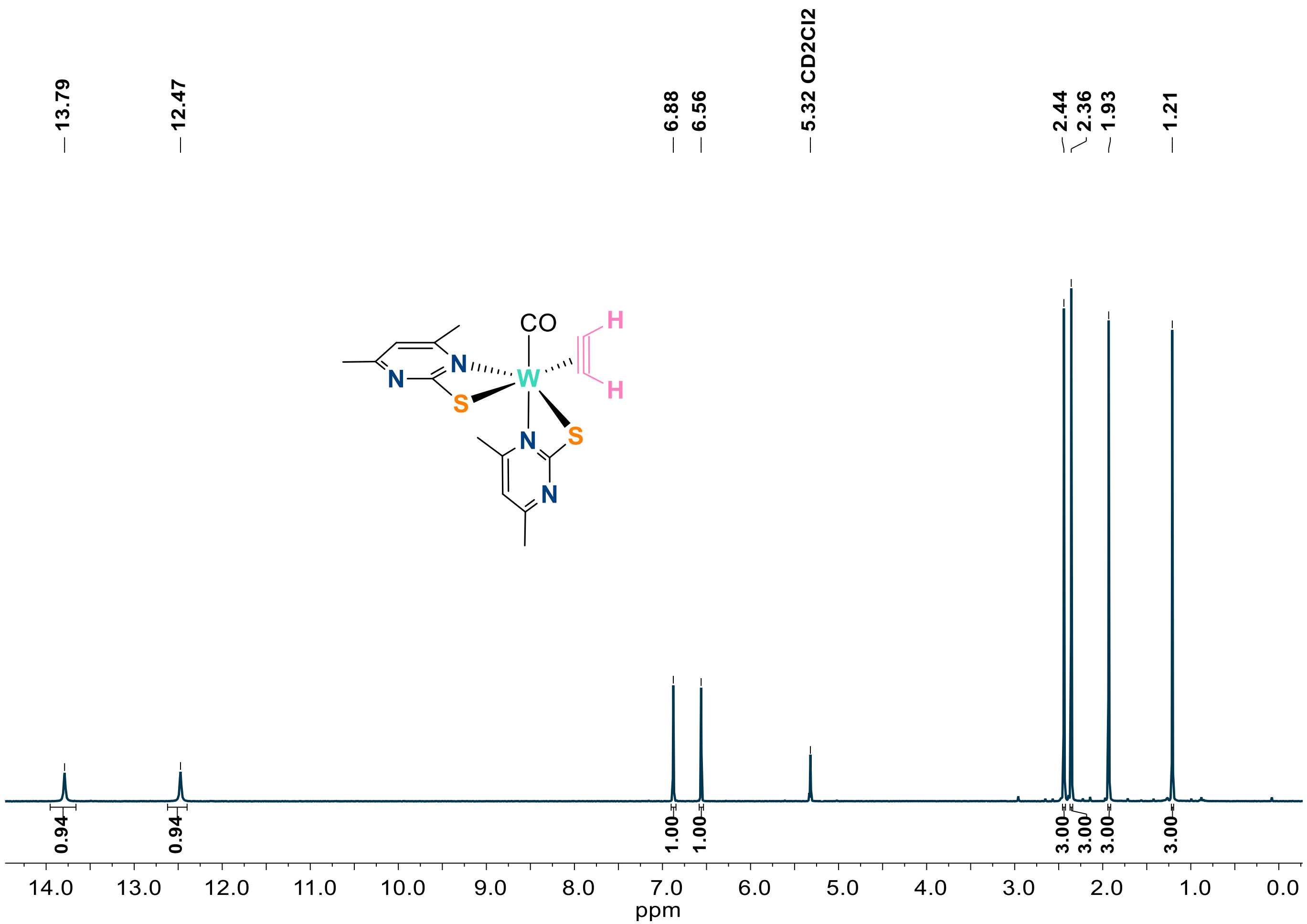

Figure S1. ^{1}H NMR spectrum of **W1** in CD_2Cl_2.

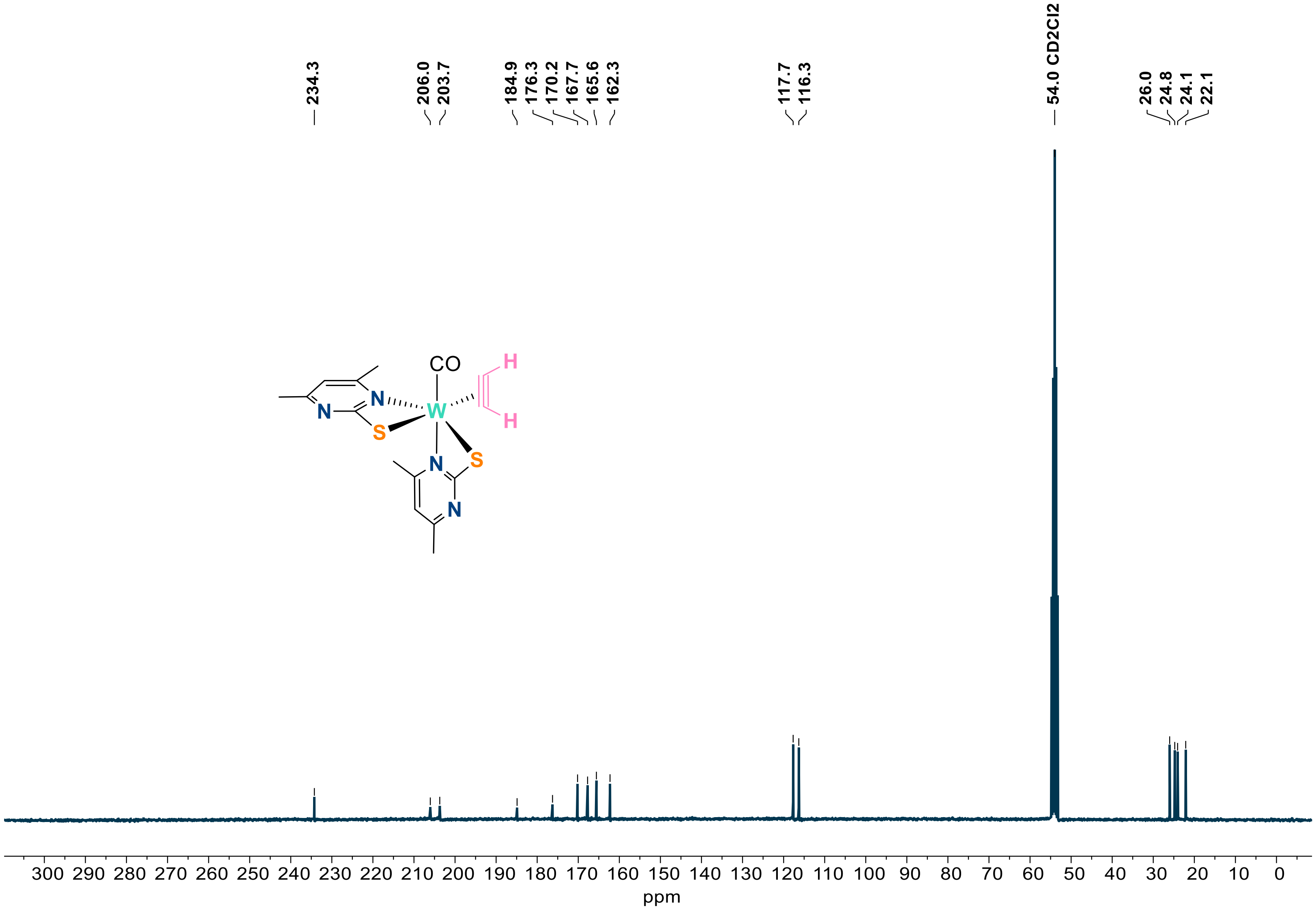

Figure S2. ^{13}C NMR spectrum of **W1** in CD_2Cl_2.

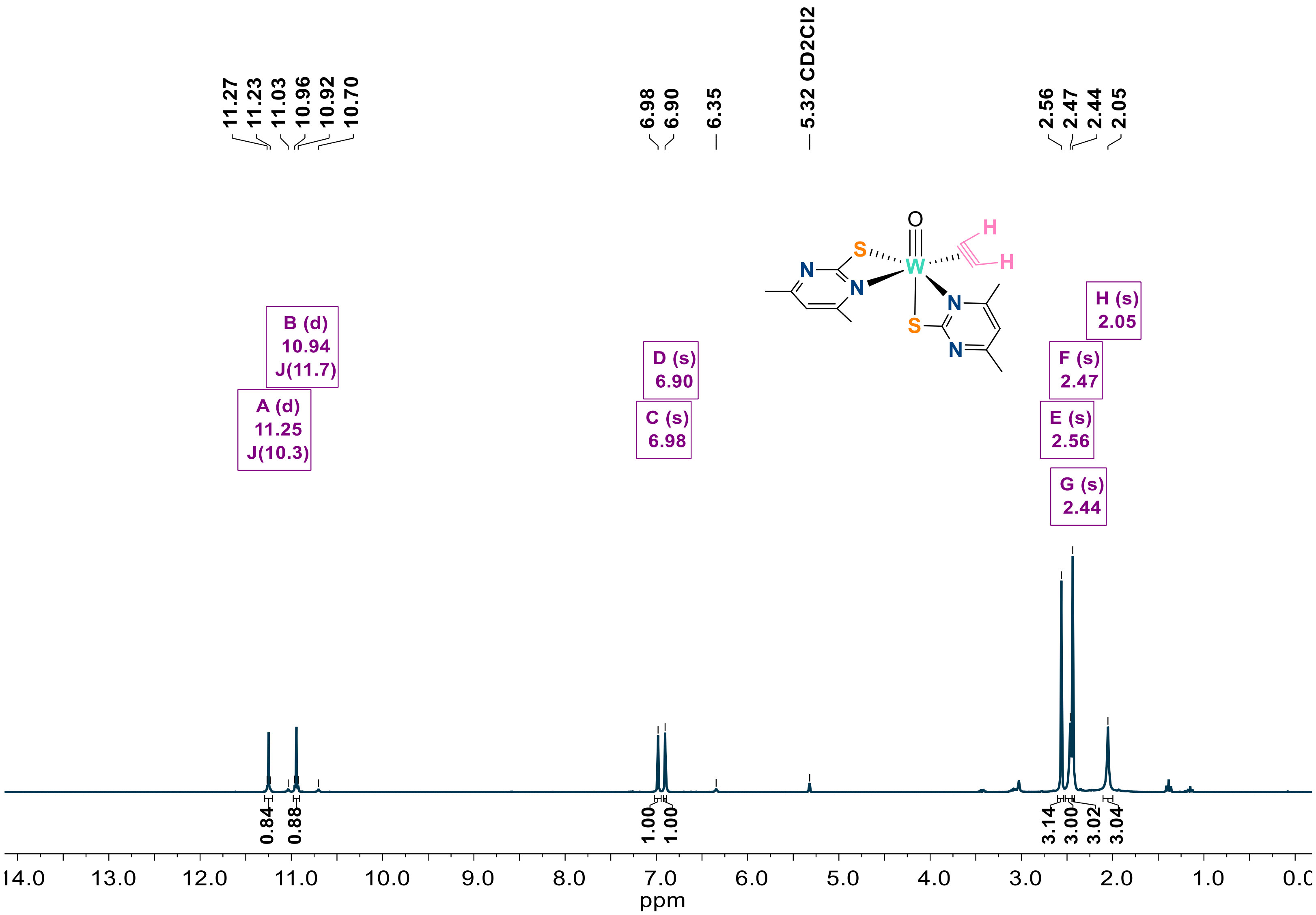

Figure S3. ^{1}H NMR spectrum of **W2** in CD_2Cl_2.

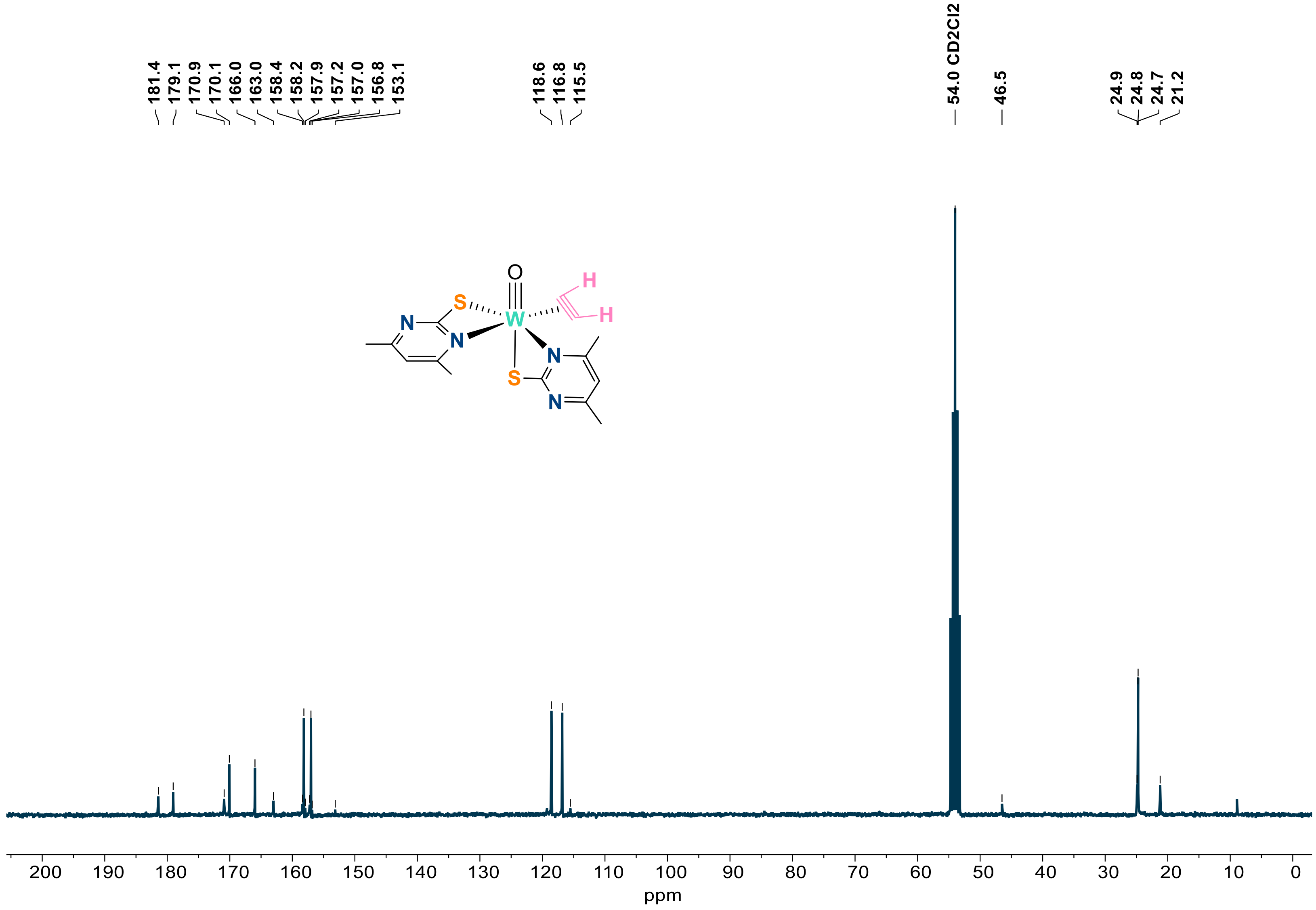

Figure S4. ^{13}C NMR spectrum of **W2** in CD_2Cl_2.

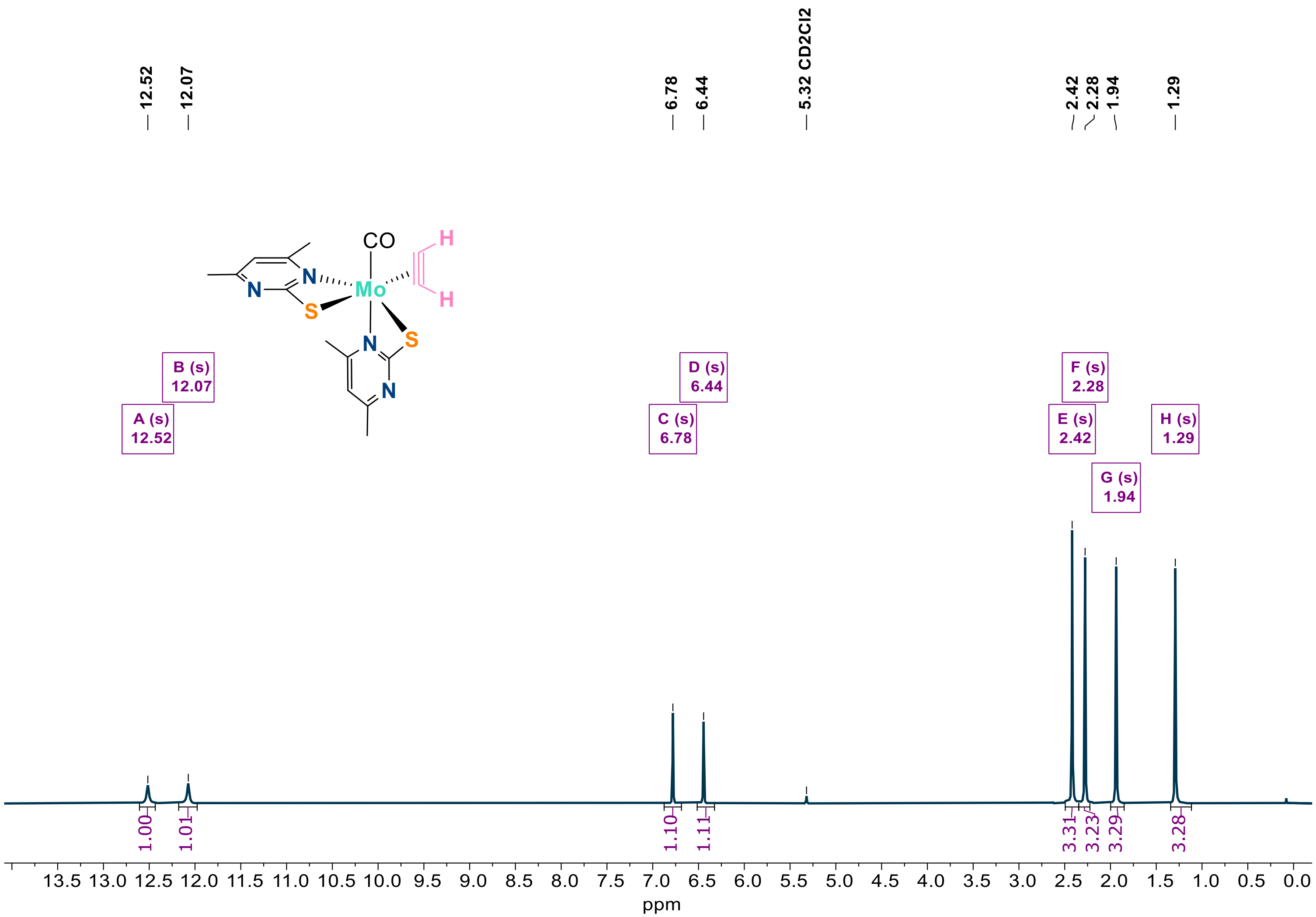

Figure S5. ^{1}H NMR spectrum of **Mo1** in CD_2Cl_2.

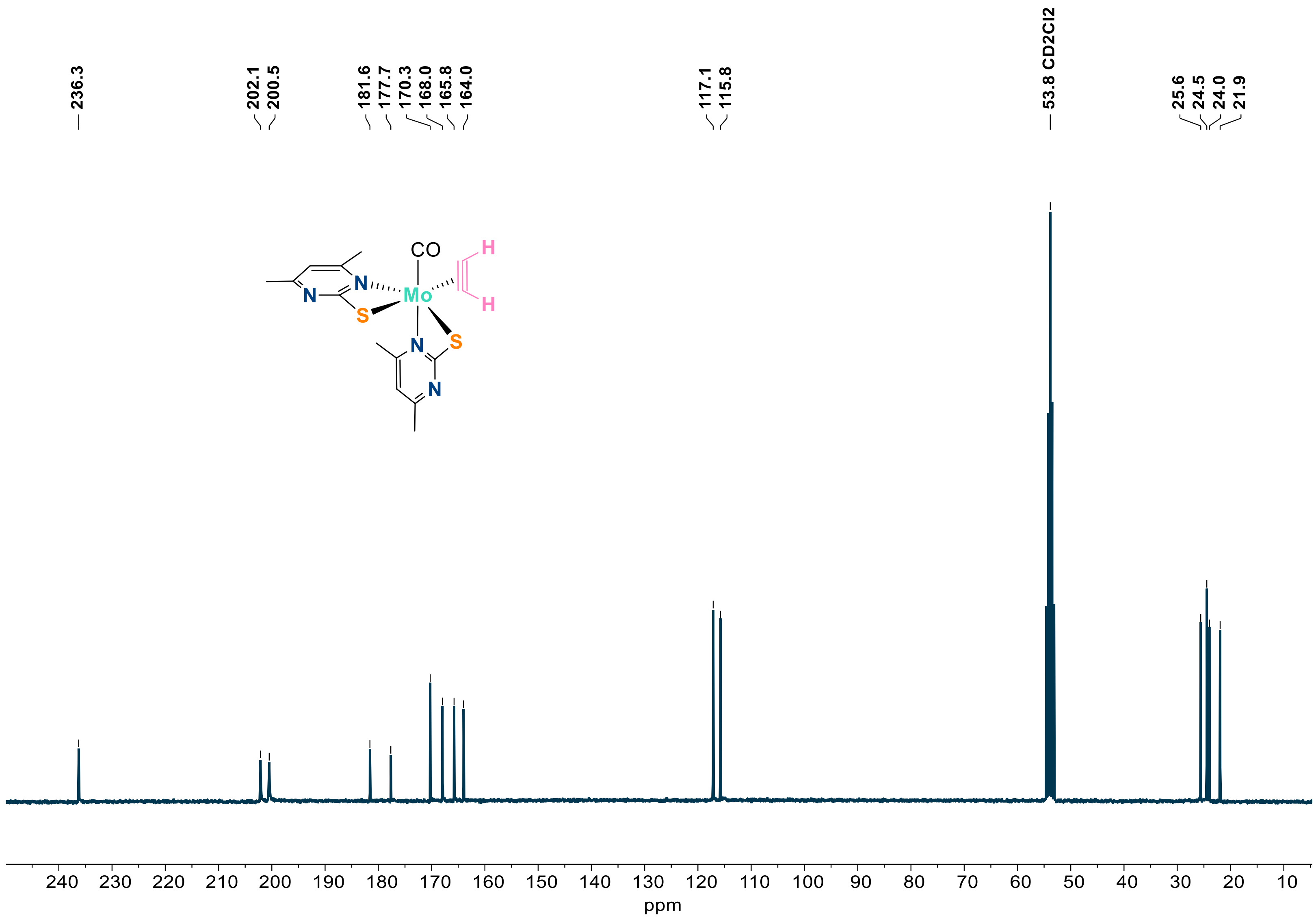

Figure S6. ^{13}C NMR spectrum of **Mo1** in CD_2Cl_2.

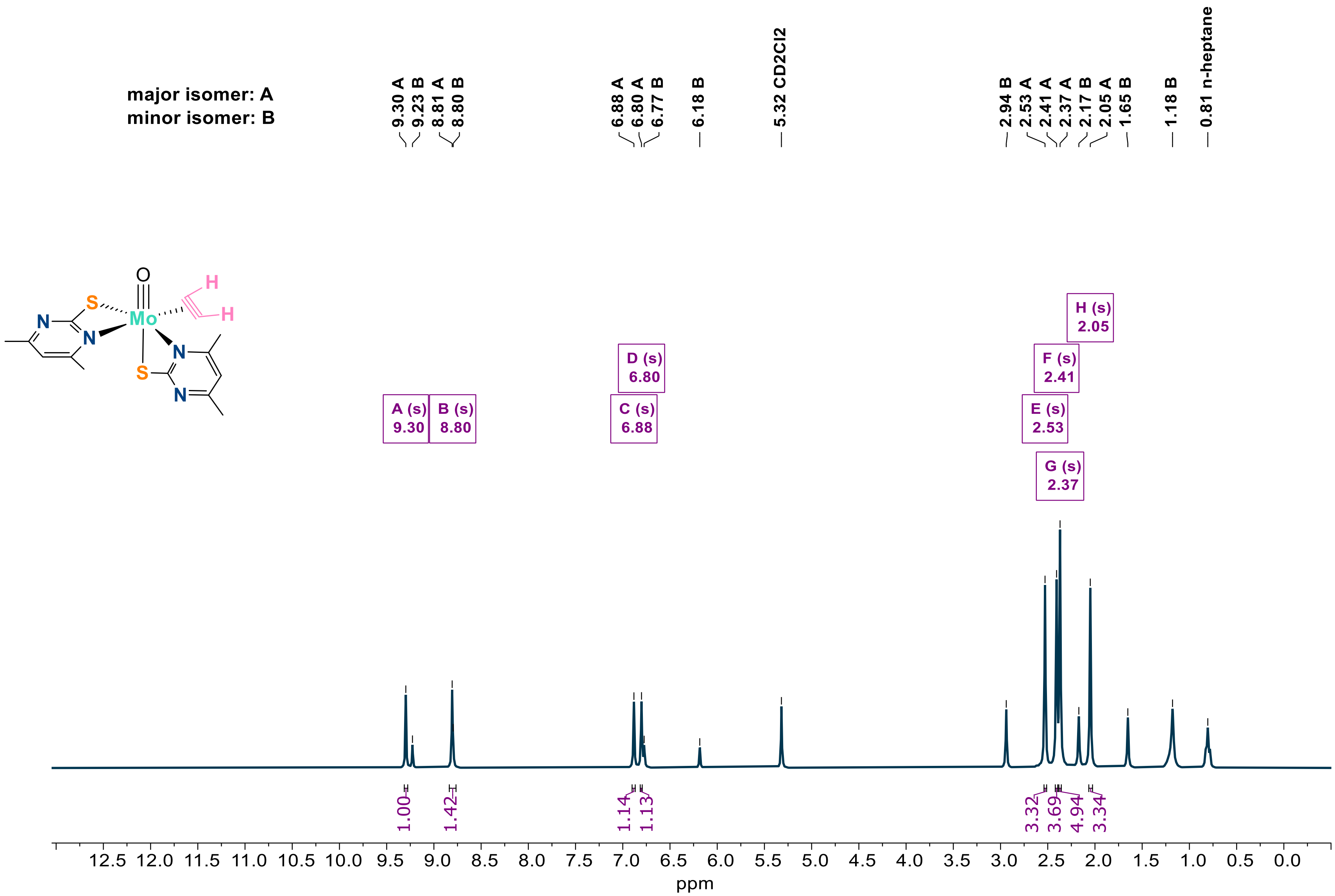

Figure S7. ^{1}H NMR spectrum of **Mo2** in CD_2Cl_2 at -40 °C. Top: Major and minor isomers are labeled as A, and B, respectively.

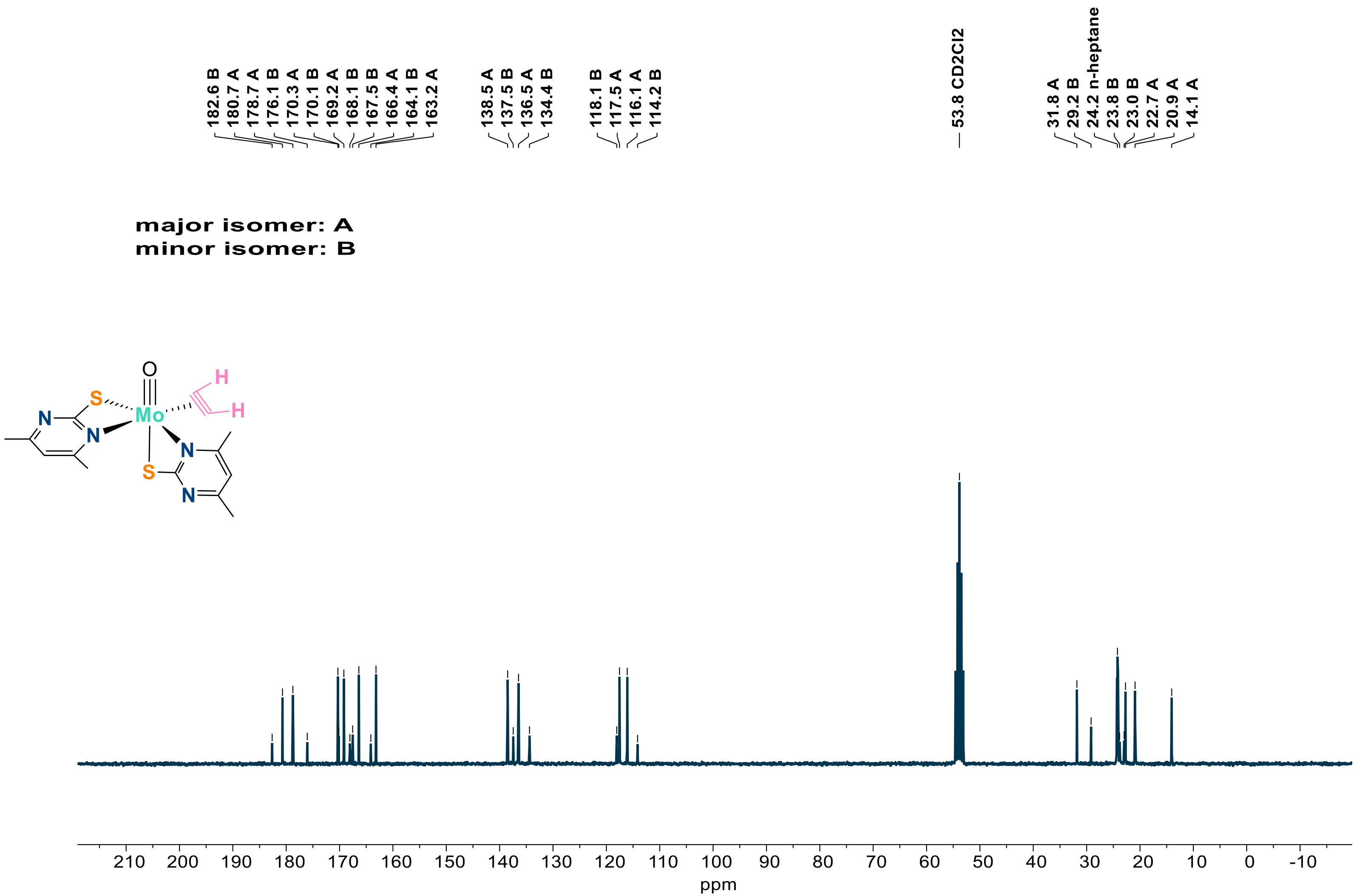

Figure S8. ^{13}C NMR spectrum of **Mo2** in CD_2Cl_2 at -40 °C. Major and minor isomer are labeled as A, and B, respectively.

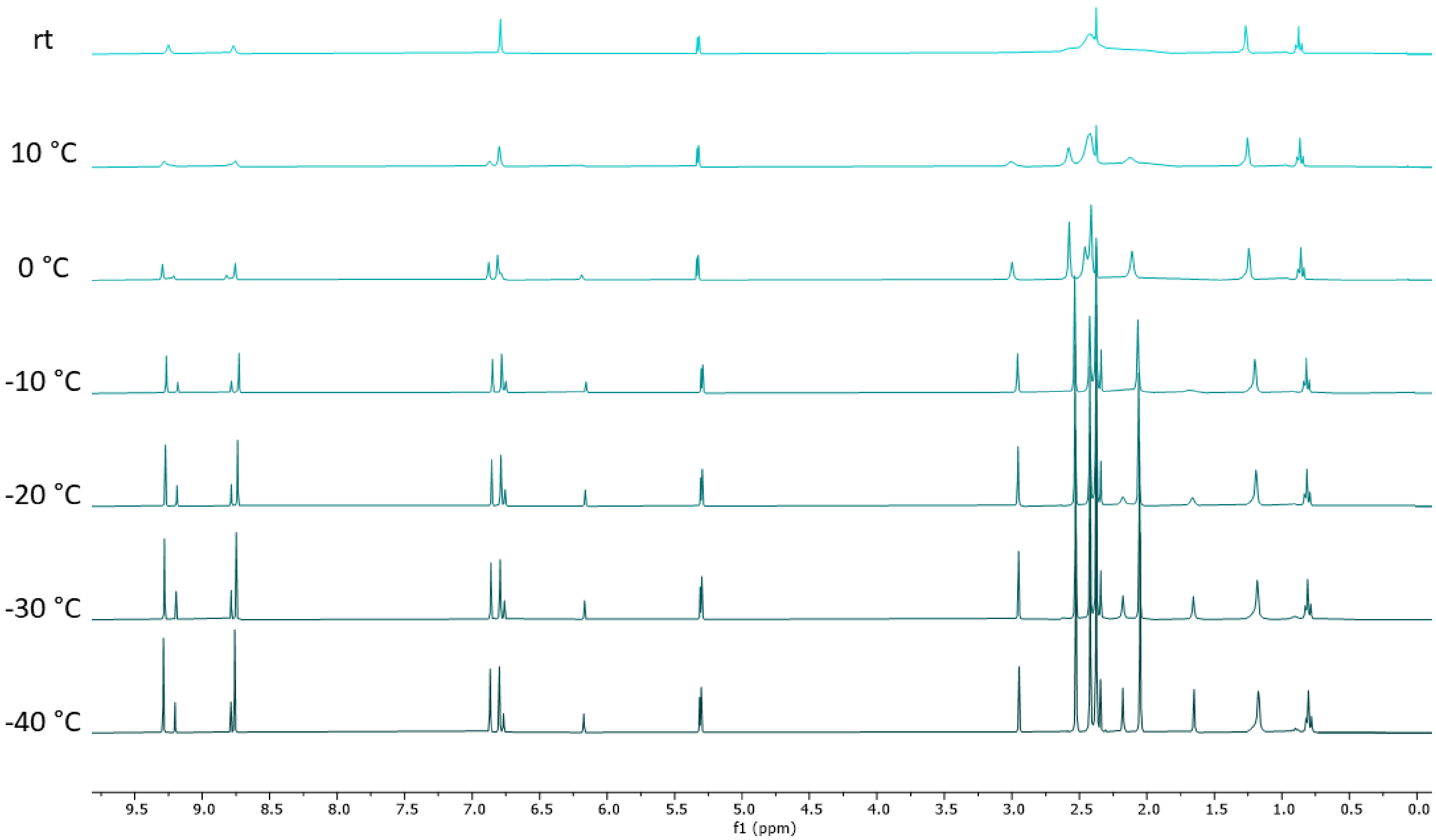

Figure S9. VT ^{1}H NMR spectra of complex **Mo2** in CD_2Cl_2 measured at rt, 10 °C, 0 °C, -10 °C, -20 °C, -30 °C, and -40 °C.

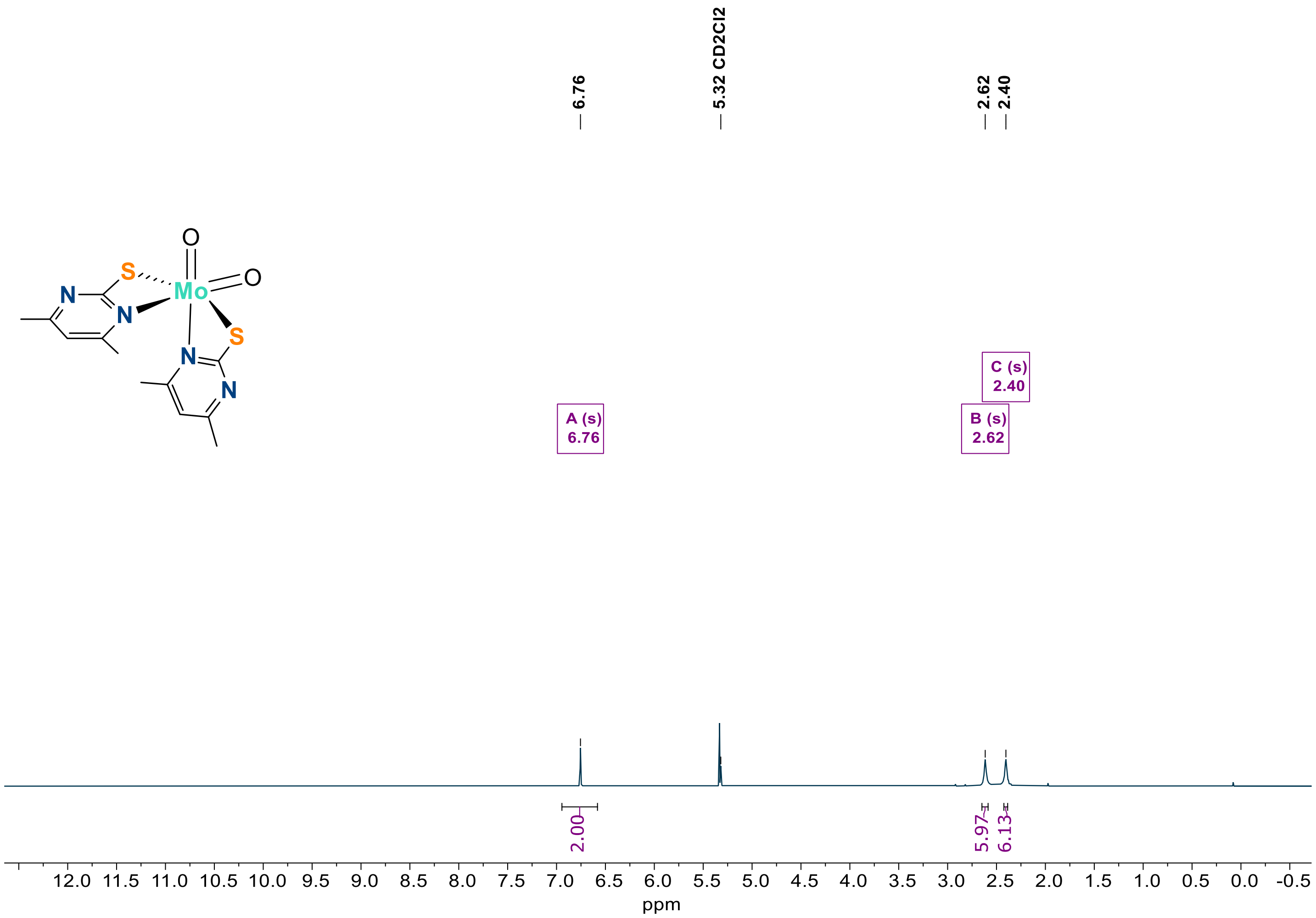

Figure S10. ^{1}H NMR spectrum of $[MoO_2(PymS)_2]$ in CD_2Cl_2.

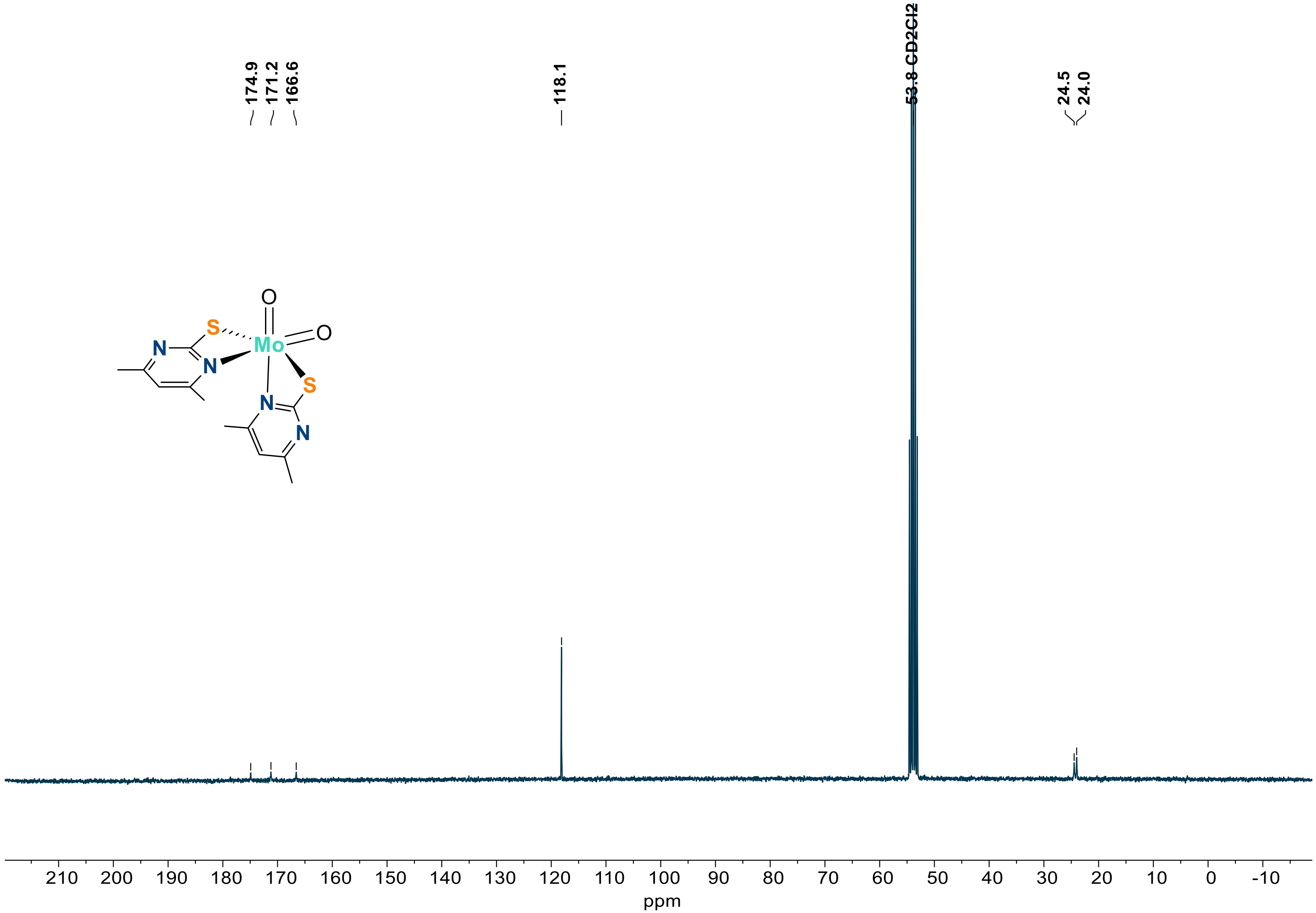

Figure S11. ^{13}C NMR spectrum of [$MoO_2(PymS)_2$] in CD_2Cl_2.

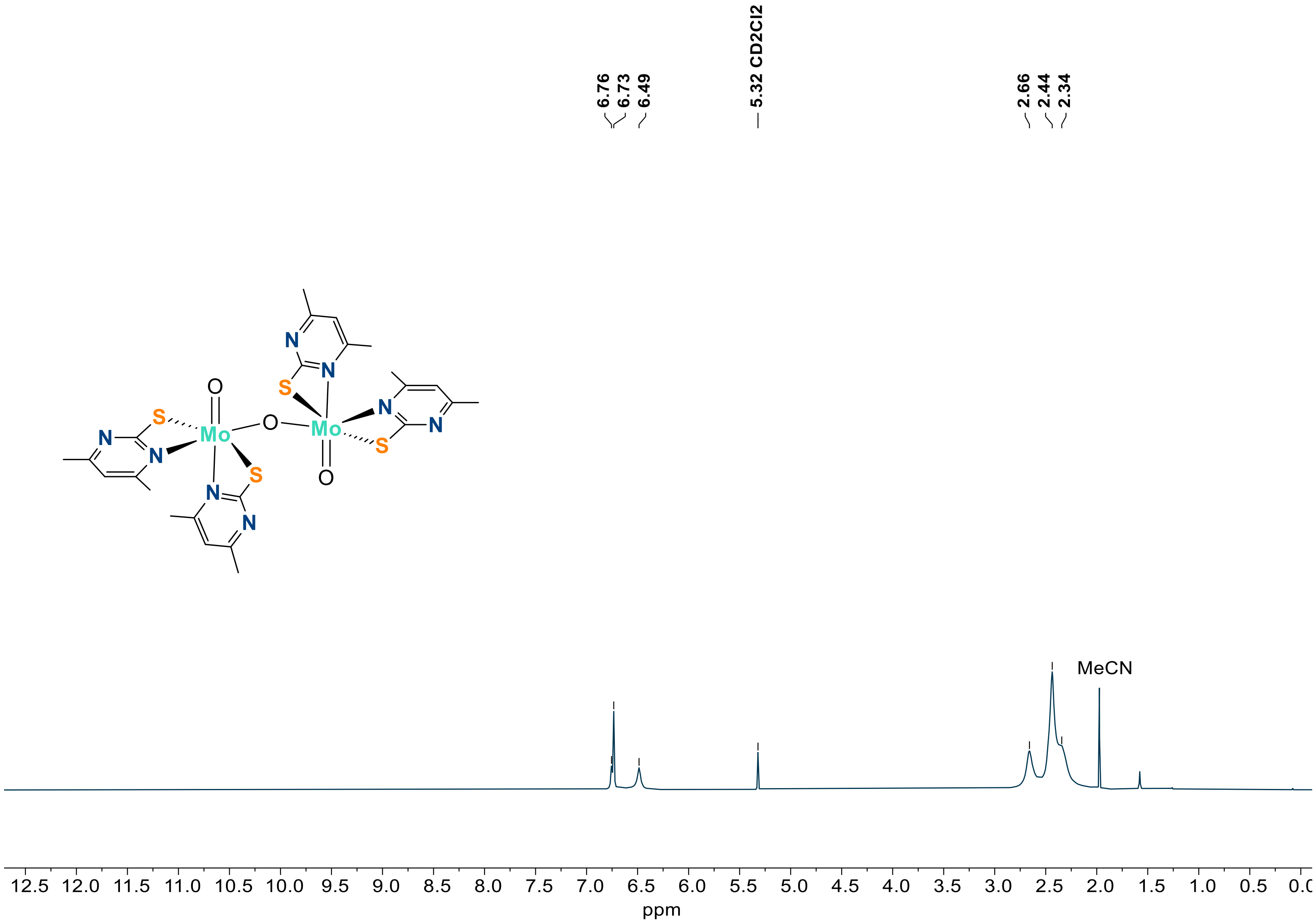

Figure S12. ^{1}H NMR spectrum of $[Mo_2O_3(PymS)_4]$ in CD_2Cl_2.

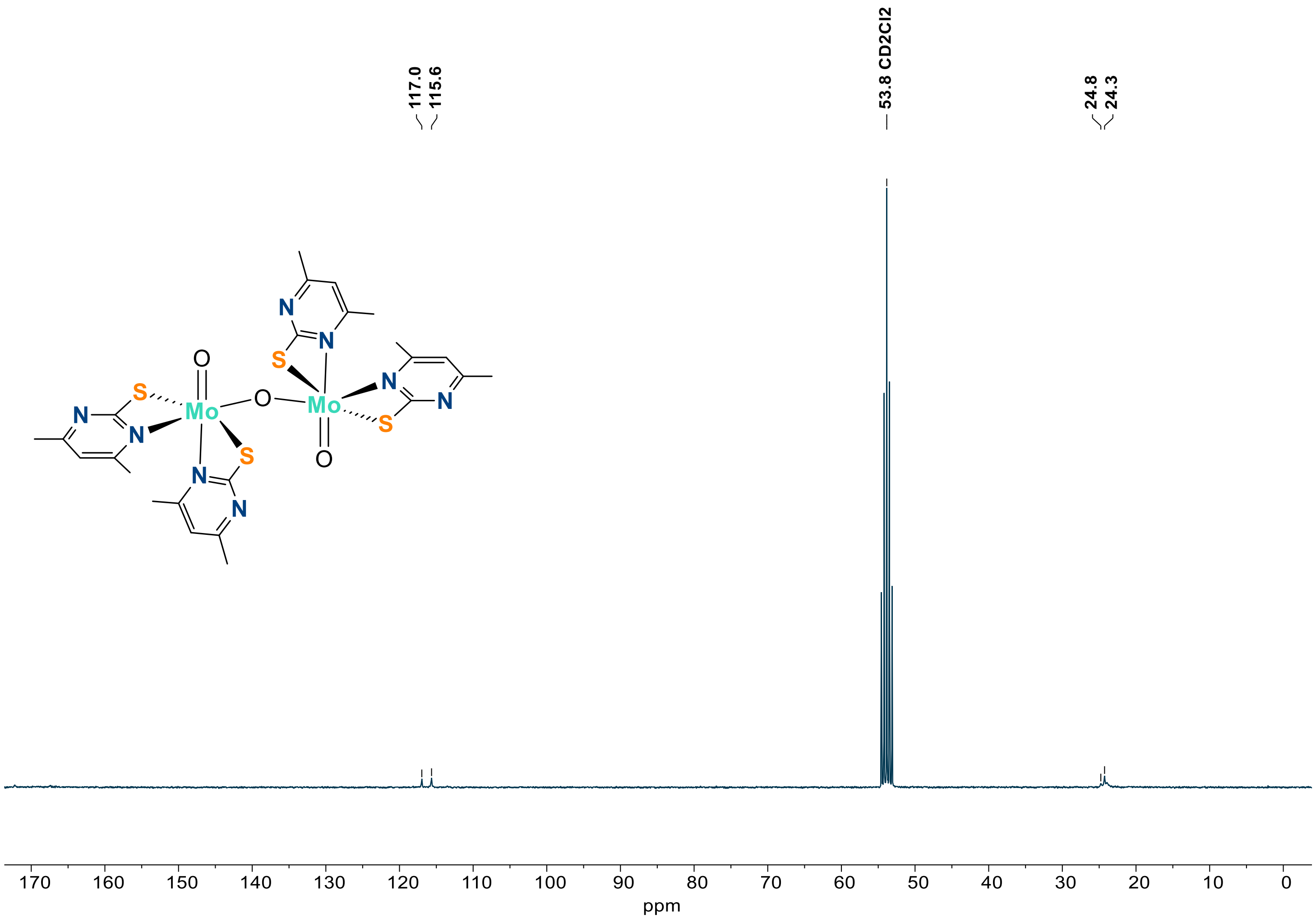

Figure S13. ^{13}C NMR spectrum of $[Mo_2O_3(PymS)_4]$ in CD_2Cl_2.

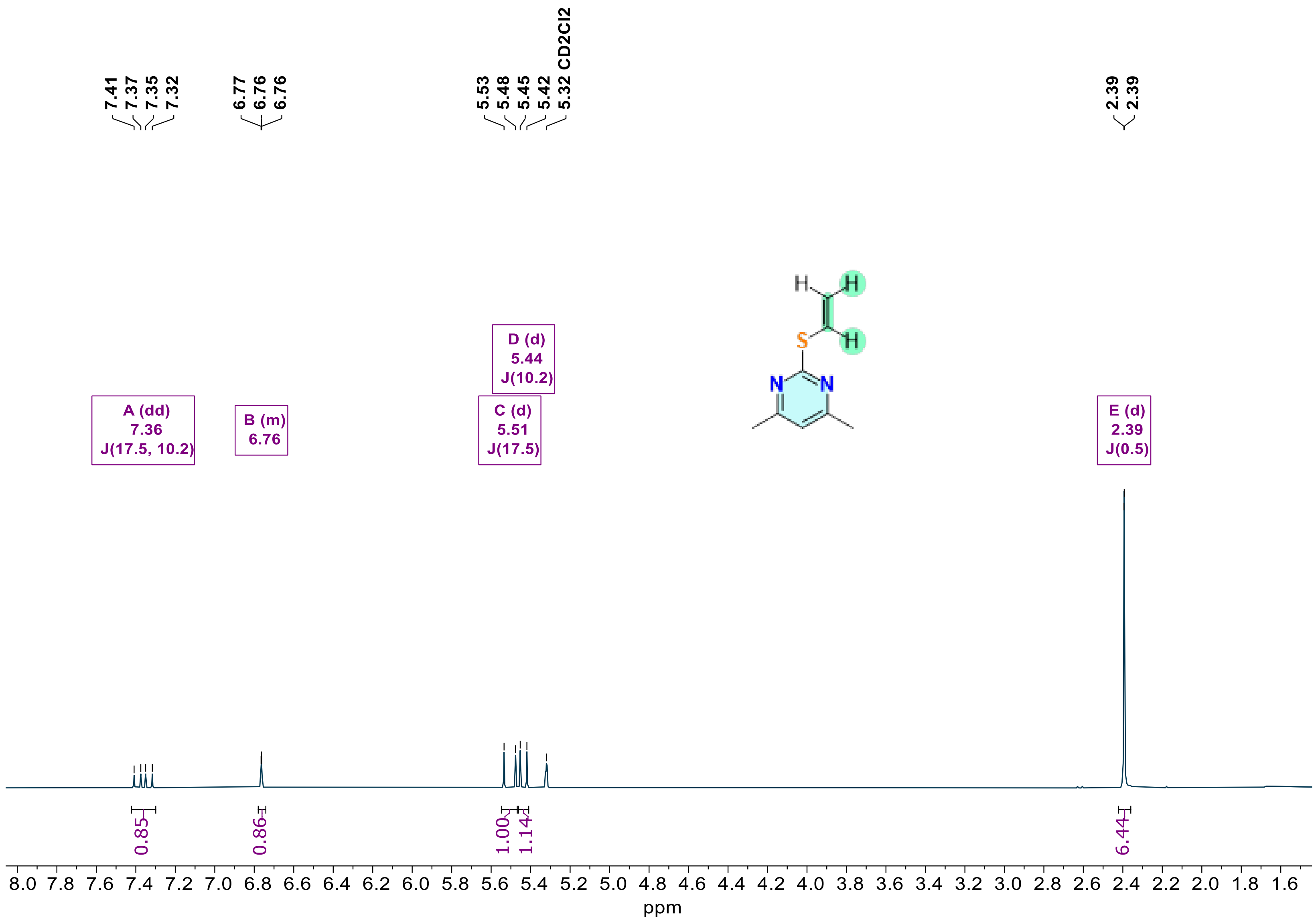

Figure S14. ^{1}H NMR spectrum of 4,6-dimethyl-2-(vinylthio)pyrimidine in CD_2Cl_2.

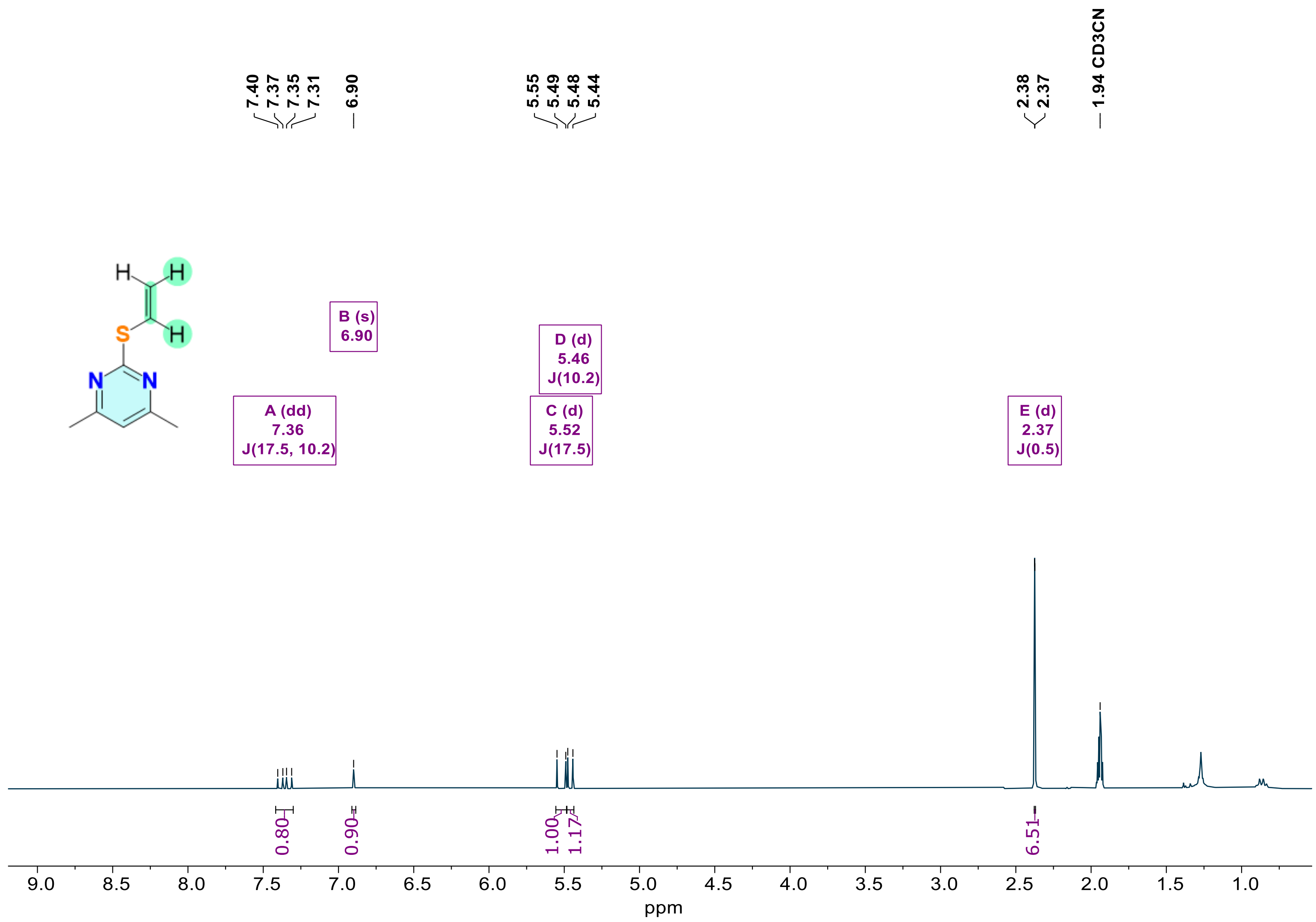

Figure S15. ^{1}H NMR spectrum of 4,6-dimethyl-2-(vinylthio)pyrimidine in CD_3CN.

4 Reactivity Studies

Reactions with trimethylamine-*N*-oxide dihydrate: The oxido compounds **Mo2** and **W2** were dissolved in CD_3CN, and reacted with varying equivalents of TMAO·$2H_2O$. Acetonitrile was chosen due to the limited solubility of TMAO·$2H_2O$ in less polar solvents. Typical experiment was performed in a 4 mL scintillation vial containing 1 mL CD_3CN solution with the complex (24 µmol), stoichiometric amount of trimethoxybenzene (internal standard) and varying equivalents of TMAO·$2H_2O$. The solutions were monitored via ^{1}H NMR spectroscopy over time. The excess of TMAO·$2H_2O$ led to the acetylene release (2.17 ppm in CD_3CN) and decomposition of the complex to free protonated ligand and the ligand disulfide, as confirmed by comparison to the NMR data from the literature.[3,7] In all the reaction, some amount of pale blue precipitate formed, which could be assigned to the polyoxometalates, commonly formed with high valent Mo- and W- compounds under oxidative conditions. Interestingly, the addition of sub-stoichiometric amounts of TMAO·$2H_2O$ to **Mo2** led to the two vinyl species namely: 4,6-dimethyl-1-vinylpyrimidine-2(1H)-thione (**A**), and 4,6-dimethyl-2-(vinylthio)pyrimidine (**B**).

A (4,6-dimethyl-1-vinylpyrimidine-2(1H)-thione) ^{1}H NMR (CD_3CN): δ 6.71 (dd, *J* = 15.8, 8.2 Hz, 1H, N-C**H**CH_2), 6.65 (s, 1H, ArH), 5.51 (dd, *J* = 8.2, 1.1 Hz, 1H, N-CHC**H**$_2$), 5.27 (dd, *J* = 15.8, 1.1 Hz, 1H, N-CHC**H**$_2$), 2.32 (s, 3H, Me group), 2.31 (s, 3H, Me group) ppm.

B (4,6-dimethyl-2-(vinylthio)pyrimidine) ^{1}H NMR (CD_3CN) δ 7.36 (dd, *J* = 17.5, 10.2 Hz, S-C**H**CH_2), 6.90 (s, 1H, ArH), 5.52 (d, *J* = 17.5 Hz, 1H, S-CHC**H**$_2$), 5.46 (d, *J* = 10.2 Hz, 1H, S-CHC**H**$_2$), 2.37 (s, 6H, Me groups) ppm.

The spectrum after 24 hours of the reaction between **Mo2** and 0.5 equiv of TMAO·$2H_2O$ shows the presence of the starting complex, internal standard, free acetylene, protonated ligand and the two vinyl species **A** and **B** (**Figure S17**). The highest yield of the vinyl species was obtained after 48h, namely 20 % of each with respect to the acetylene ligand. In contrast, the tungsten variant **W2** does not yield any vinyl-product, no matter of the equivalents of TMAO·$2H_2O$ added. Our attempts to prepare species **A** via alternative routes, which involved alkylation of pyrimidine-N with 1,2-dibromoethane and subsequent elimination failed, most likely for the high nucleophilicity of the neighboring sulfur and cyclization. Nevertheless, the vinyl group protons of **A** resonate in almost identical region as the *N*-vinyl compounds previously reported by our group.[8] To confirm the occurrence of species **B** in our reaction, it was separately prepared by literature methods for comparison.[9] For better visualization, two identical spectra (Figure S18 and Figure S19) of the reaction mixture containing **Mo2** with 0.5 equiv TMAO·$2H_2O$ in CD_3CN are presented and only the peaks corresponding to species **A** and **B** are labeled.

m/z Determination: The solvent was removed from an NMR sample containing the reaction mixture. The residue was extracted with CH_2Cl_2, washed with water and dried. The residue was redissolved in MeCN and passed through a 0.22 μm Nylon syringe filter. The filter was then washed with methanol (2 × 250 μL) and the combined filtrate was collected in a 1.5 mL HPLC glass vial. An Agilent 1200 series HPLC system comprised of a binary pump, an autosampler, a temperature-controlled column compartment, a diode-array-detector, and a 6120 quadrupole ESI-MS detector was used for the analysis of the sample. A YMC-Triart C18 (150 × 2.1 mm, 5 μm) was used as the stationary phase maintained at 50 °C during analysis. The mobile phase comprised of a 60:40 mixture of methanol and 0.5% formic acid in ultrapure water 60:40 at a flow rate of 0.300 mL/min. Two peaks corresponding to an m/z of 167 were detected and correlated to UV-absorption

bands at 280 nm, and 260 nm, respectively. Both are assigned as $[M+H]^+$ ions of the species **A** and **B**, further confirming their presence in the sample.

Hydrolysis of B (4,6-dimethyl-2-(vinylthio)pyrimidine)

In a J. Young NMR tube, 10 mg of **B** were dissolved in 0.5 mL of CD_3CN and the tube was heated at 80 °C overnight leading to full hydrolysis of the starting material to acetaldehyde.

Control Experiments

All experiments were conducted in CD_3CN, except for entry 2, which was performed in CD_2Cl_2 due to solubility reasons. 1,3,5-Trimethoxybenzene was used as an internal standard in all cases. The spectroscopic yields of vinyl compounds **A** and **B** were determined by ^{1}H NMR spectroscopy after 48 hours. Detailed experimental procedures are provided below the **Table S1**.

Table S1. Control experiments for the C_2H_2 activation, including alternative reagents, radical traps, and blanks.

No.	Description	C_2H_2 source	Oxidant	Additive	**A**, yield	**B**, yield
1	standard	**Mo2**	TMAO·$2H_2O$	/	20 %	20 %
2	alternative OAT donor	**Mo2**	$MoO_2(PymS)_2$	H_2O	15 %	15 %
3	alternative oxidant (no OAT)	**Mo2**	Magic blue	H_2O	n.d.	n.d.
4	no Mo complex	C_2H_2	TMAO·$2H_2O$	Na(PymS)	n.d.	n.d.
5	more thiolate	**Mo2**	TMAO·$2H_2O$	Na(PymS)	25 %	75 %
6	radical trap	**Mo2**	TMAO·$2H_2O$	Galvioxyl	n.d.	n.d.
7	reductive pathway	C_2H_2	$MoO_2(PymS)_2$	Cp_2Co (1 equiv), H_2O	n.d.	Quant.

Reactions without TMAO but with the alternative oxygen atom source $[MoO_2(PymS)_2]$:

Without water: In a J. Young NMR tube, **Mo2** (8 µmol, 1 equiv) and $[MoO_2(PymS)_2]$ (8 µmol, 1 equiv) were dissolved in CD_2Cl_2 (0.5 mL). The ^1H NMR spectrum was recorded immediately after mixing (see Figure S21B). Spectrum B reveals the starting compounds as well as broad peaks deriving from the dimer $[Mo_2O_3(PymS)_4]$. Additionally, UV-Vis spectra were recorded for diluted solutions of the **Mo2** complex and $[Mo_2O_3(PymS)_4]$ in CH_2Cl_2, both individually and after mixing. These measurements were performed to corroborate the color change observed upon mixing, as illustrated in **Figure S16**.

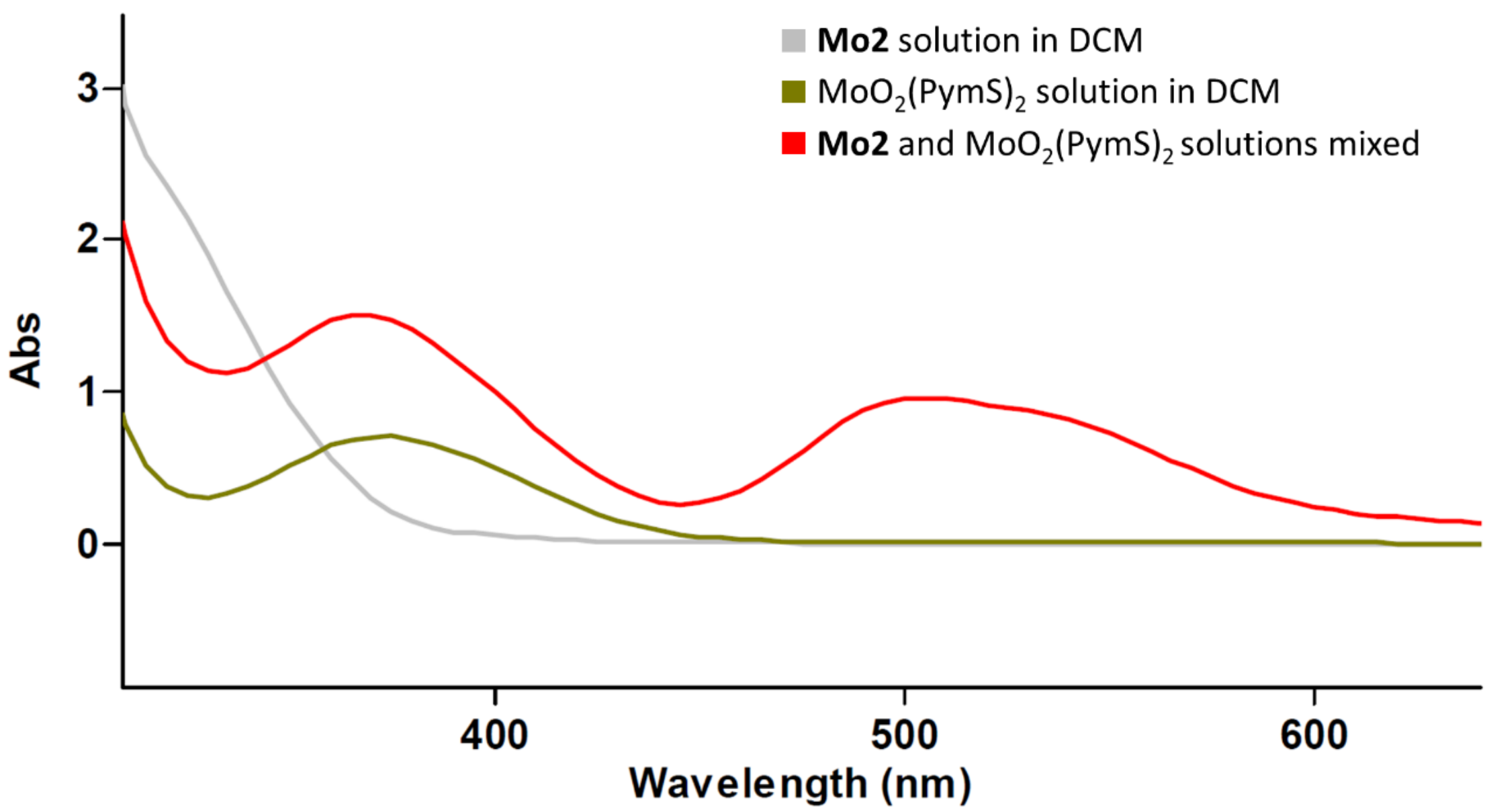

Figure S16. UV-Vis spectra of CH_2Cl_2 solutions of **Mo2**, $[Mo_2O_3(PymS)_4]$, and upon mixing the solutions of **Mo2** and $[Mo_2O_3(PymS)_4]$

With water: In a J. Young NMR tube, **Mo2** (8 µmol, 1 equiv), $[MoO_2(PymS)_2]$ (8 µmol, 1 equiv), trimethoxybenzene (8.8 µmol, 1.1 equiv), and water (240 µmol, 30 equiv) were combined in CD_2Cl_2 (0.5 mL). The reaction progress was monitored by ^1H NMR spectroscopy, and the final spectrum after 3 days is shown in Figure S21C.

Reactions with an alternative oxidant, no OAT:

In a 4 mL scintillation vial, **Mo2** (24 µmol, 1 equiv), magic blue $[(p\text{-}BrC_6H_4)_3N][SbCl_6]$ (24 µmol, 1 equiv), trimethoxybenzene (12 µmol, 0.5 equiv), and water (1 µmol, approx. 2 equiv) were combined in CD_3CN (1 mL). The vial was sealed and the reaction mixture was stirred at room temperature for 48 hours. After this period, the reaction mixture was analyzed by recording a ^1H NMR spectrum. No vinyl products were detected.

Reactions without metal:

In a 20 mL Schlenk tube, Na(PymS) (24 µmol, 1 equiv), TMAO·$2H_2O$ trimethoxybenzene (12 µmol, 0.5 equiv), were combined in CD_3CN (1 mL). Acetylene was bubbled through the solution for 5 minutes, after which the tube was sealed and the reaction mixture was stirred at room temperature for 48 hours. After this period, the reaction mixture was analyzed by recording a ^1H NMR spectrum. No vinyl products were detected.

Addition of excess thiolate Na(PymS):

To a standard reaction mixture containing **Mo2** (24 µmol, 1 equiv), TMAO·$2H_2O$ (12 µmol, 0.5 equiv), trimethoxybenzene (12 µmol, 0.5 equiv) in 1 mL CD_3CN, Na(PymS) (24 µmol, 1 equiv) was added. The vial was sealed and the reaction mixture was stirred at room temperature for 48 hours. After this period, the reaction mixture was analyzed by recording a ^1H NMR spectrum. An increase in the yield of the S-vinyl species has been detected (see Figure S23).

Addition of galvinoxyl as a radical scavenger:

To a standard reaction mixture containing **Mo2** (24 µmol, 1 equiv), TMAO·$2H_2O$ (12 µmol, 0.5 equiv), trimethoxybenzene (12 µmol, 0.5 equiv) in 1 mL

CD_3CN, galvinoxyl (24 µmol, 1 equiv) was added. The vial was sealed and the reaction mixture was stirred at room temperature for 48 hours. After this period, the reaction mixture was analyzed by recording a ^1H NMR spectrum. No vinyl products were detected.

Reductive pathway (1 equiv of Cp_2Co)

In a 20 mL Schlenk tube, $MoO_2(PymS)_2$ (80 µmol, 1 equiv) and cobaltocene (80 µmol, 1 equiv) were combined in CD_3CN (2 mL). The flask was removed from the glovebox and degassed water (1.5 µL) was added under inert atmosphere. The reaction mixture was then purged with acetylene for 5 minutes and the flask was sealed and stirred at room temperature for 48 hours. After this period, the reaction mixture was analyzed by recording a ^1H NMR spectrum. A quantitative yield of S-vinyl product has been determined vs. the cobaltocenium peak alongside several unidentified products (see Figure S23).

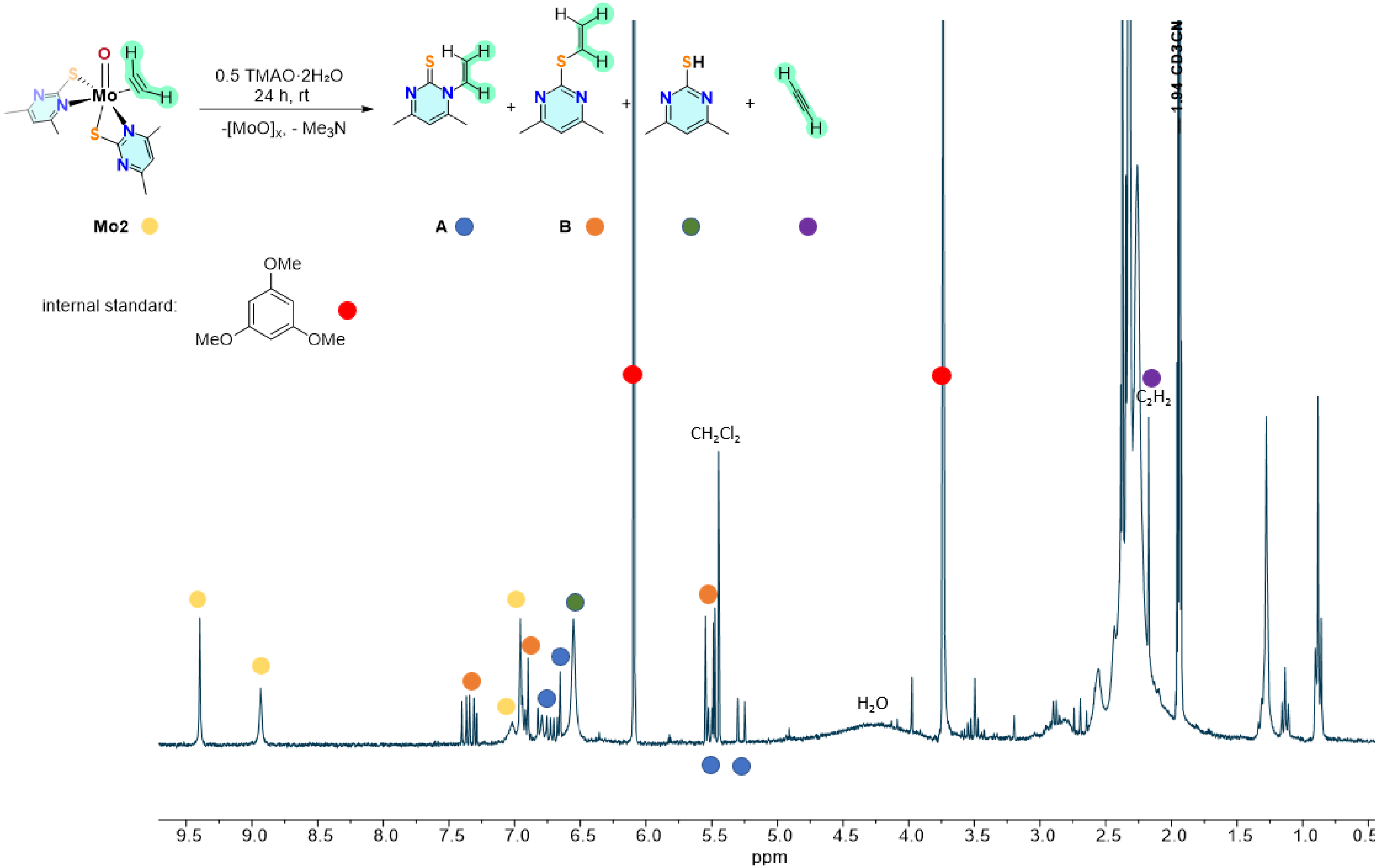

Figure S17. ^{1}H NMR spectrum of the reaction of **Mo2** with 0.5 equiv of TMAO·2H_2O in CD_3CN after 24 hours of stirring. The spectrum contains the starting complex, internal standard and several reaction products, all labeled with differently colored circles.

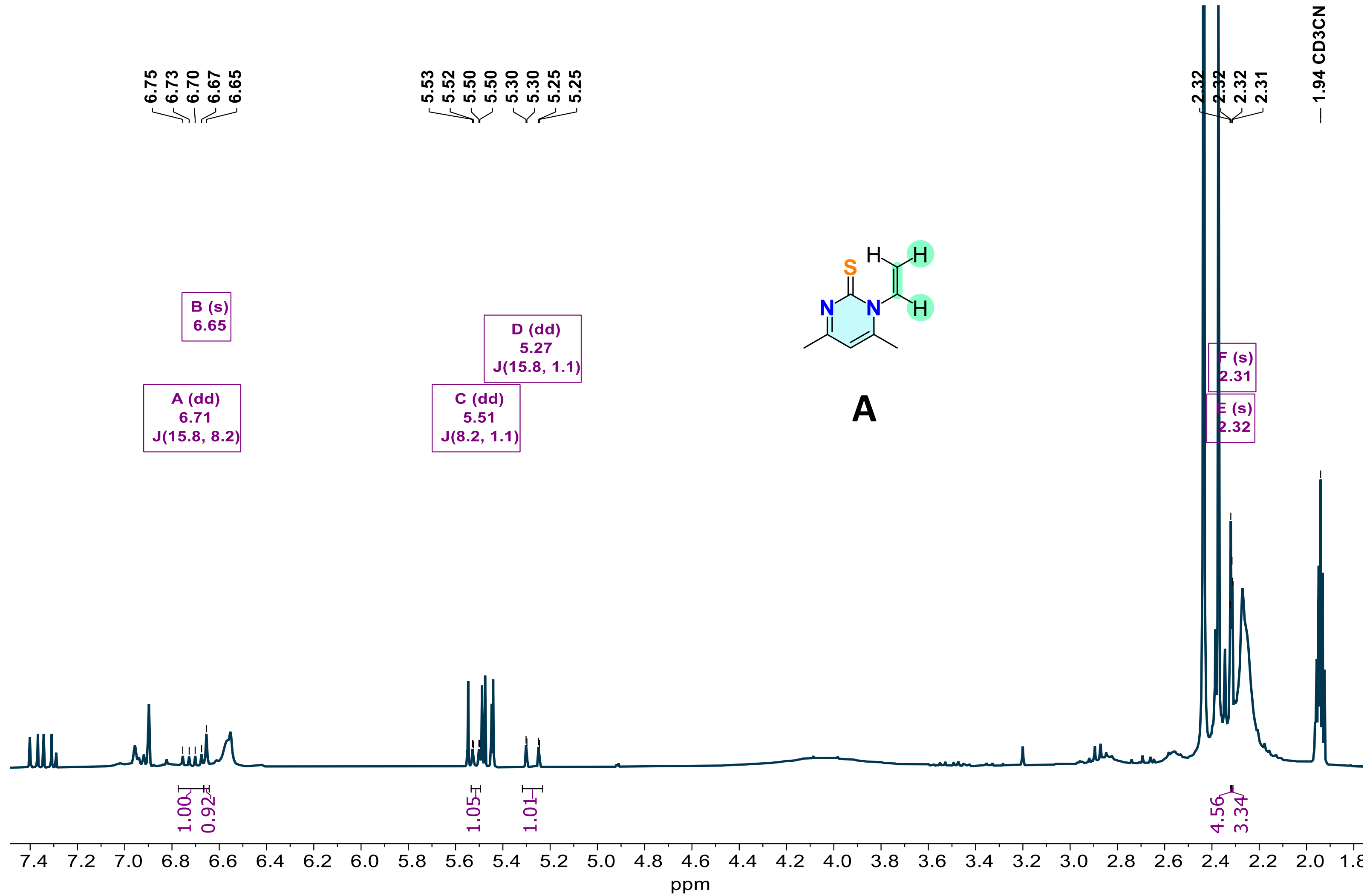

Figure S18. ^{1}H NMR spectrum of the reaction between the **Mo2** with 0.5 equiv TMAO·$2H_2O$ in CD_3CN after 48 h. Only the signals deriving from 4,6-dimethyl-1-vinylpyrimidine-2(1H)-thione (**A**) are labeled.

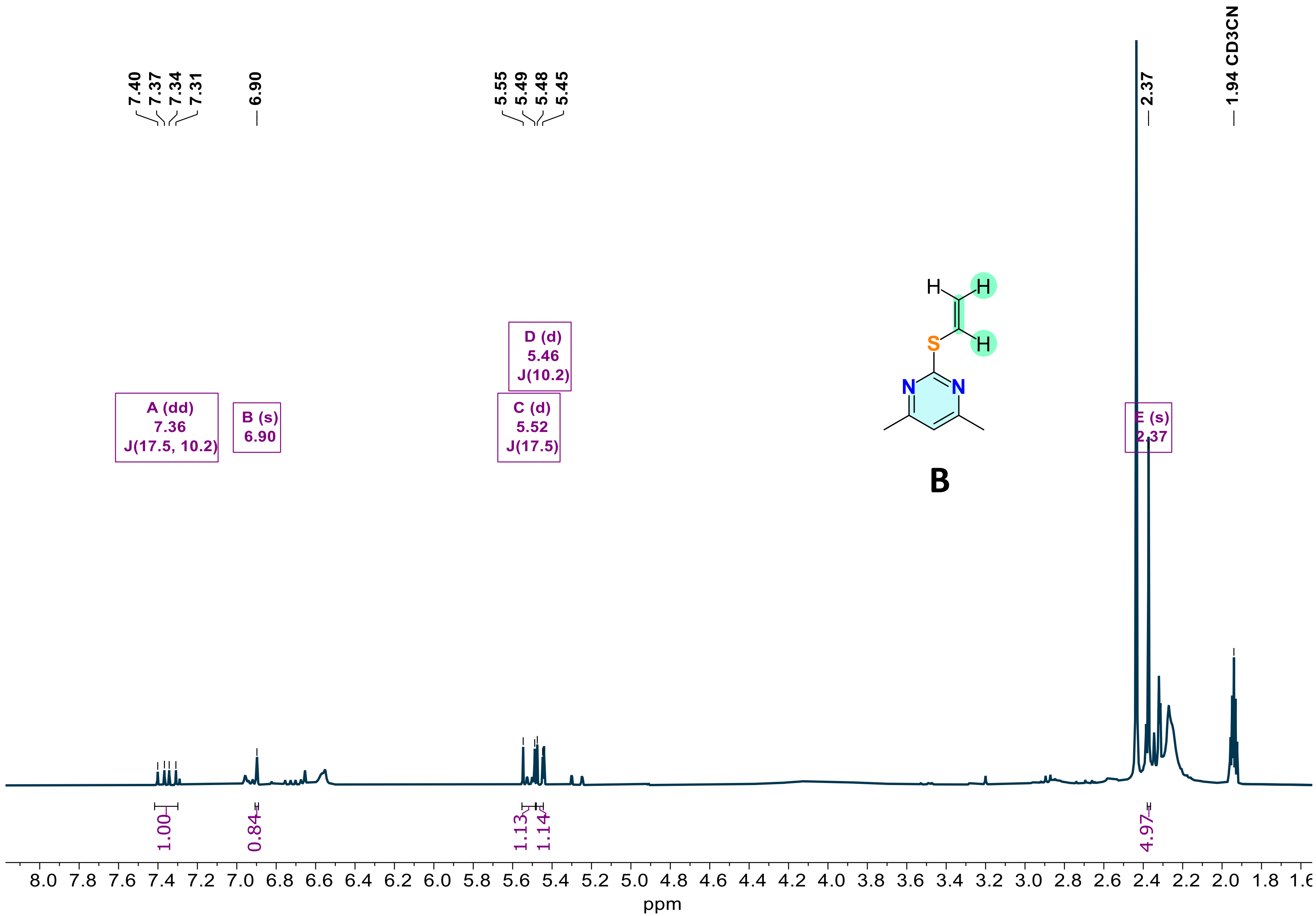

Figure S19.^{1}H NMR spectrum of the reaction between the **Mo2** with 0.5 equiv TMAO·2H_2O in CD_3CN after 48 h. Only the signals deriving from 4,6-dimethyl-2-(vinylthio)pyrimidine (**B**) are labeled.

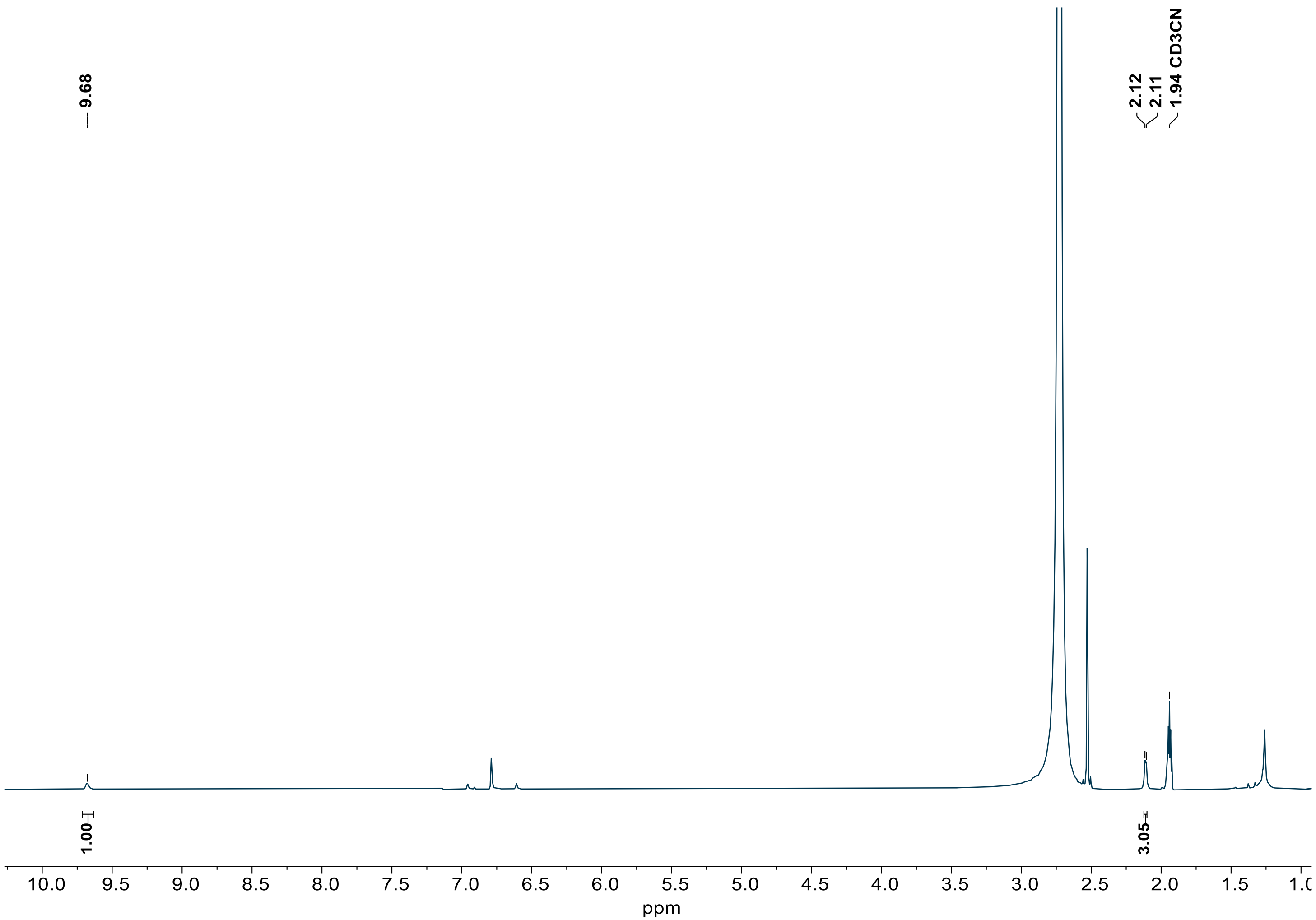

Figure S20. Adding concentrated HCl to a CD_3CN solution of 4,6-dimethyl-2-(vinylthio)pyrimidine (**B**) and heating at 80°C overnight leads to its hydrolysis to acetaldehyde. In this ^{1}H NMR spectrum, peaks belonging to acetaldehyde are labeled.

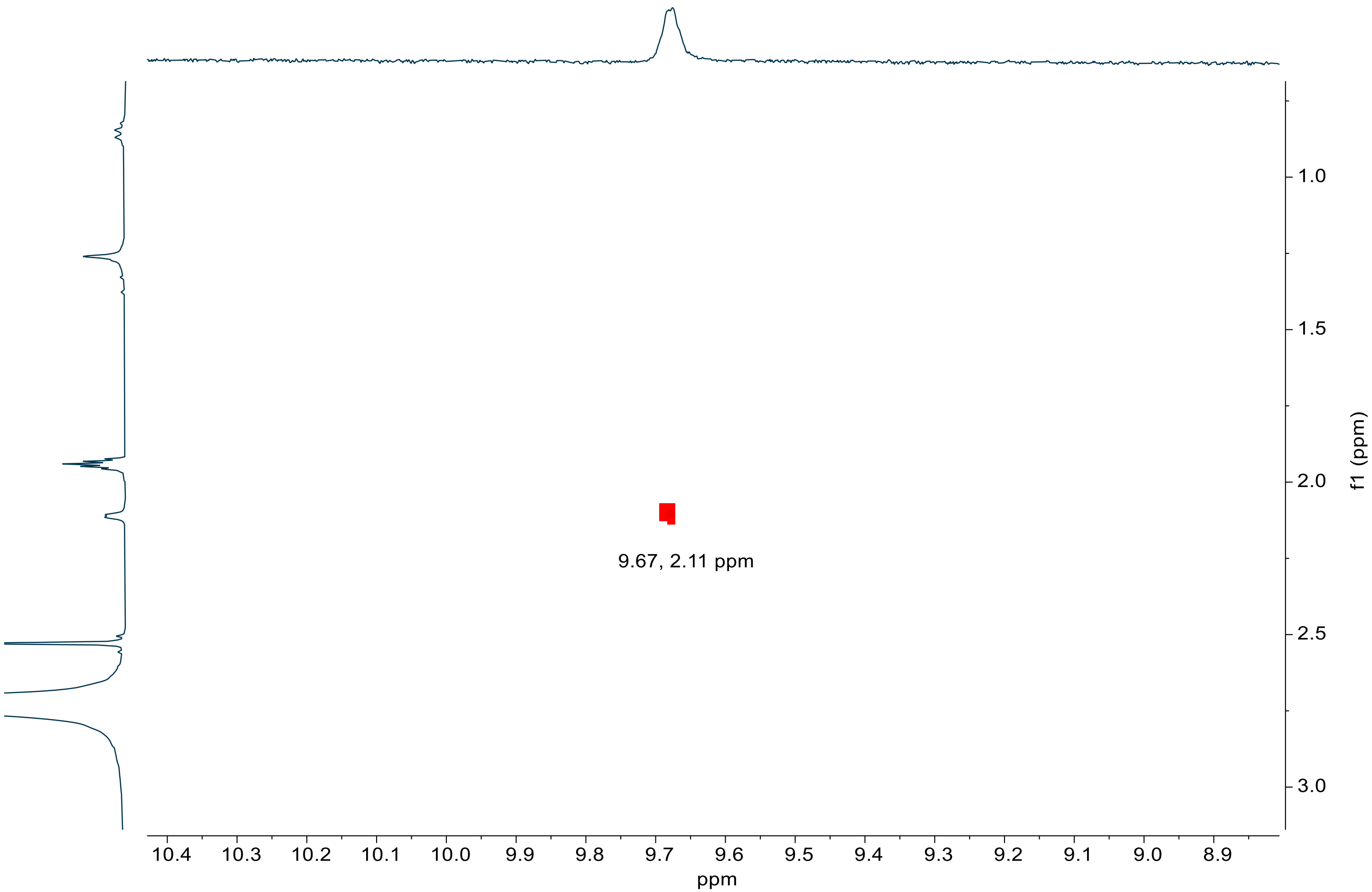

Figure S21. {^{1}H-^{1}H} COSY spectrum showing the crosspeak assigned to acetaldehyde from the hydrolysis of 4,6-dimethyl-2-(vinylthio)pyrimidine (**B**).

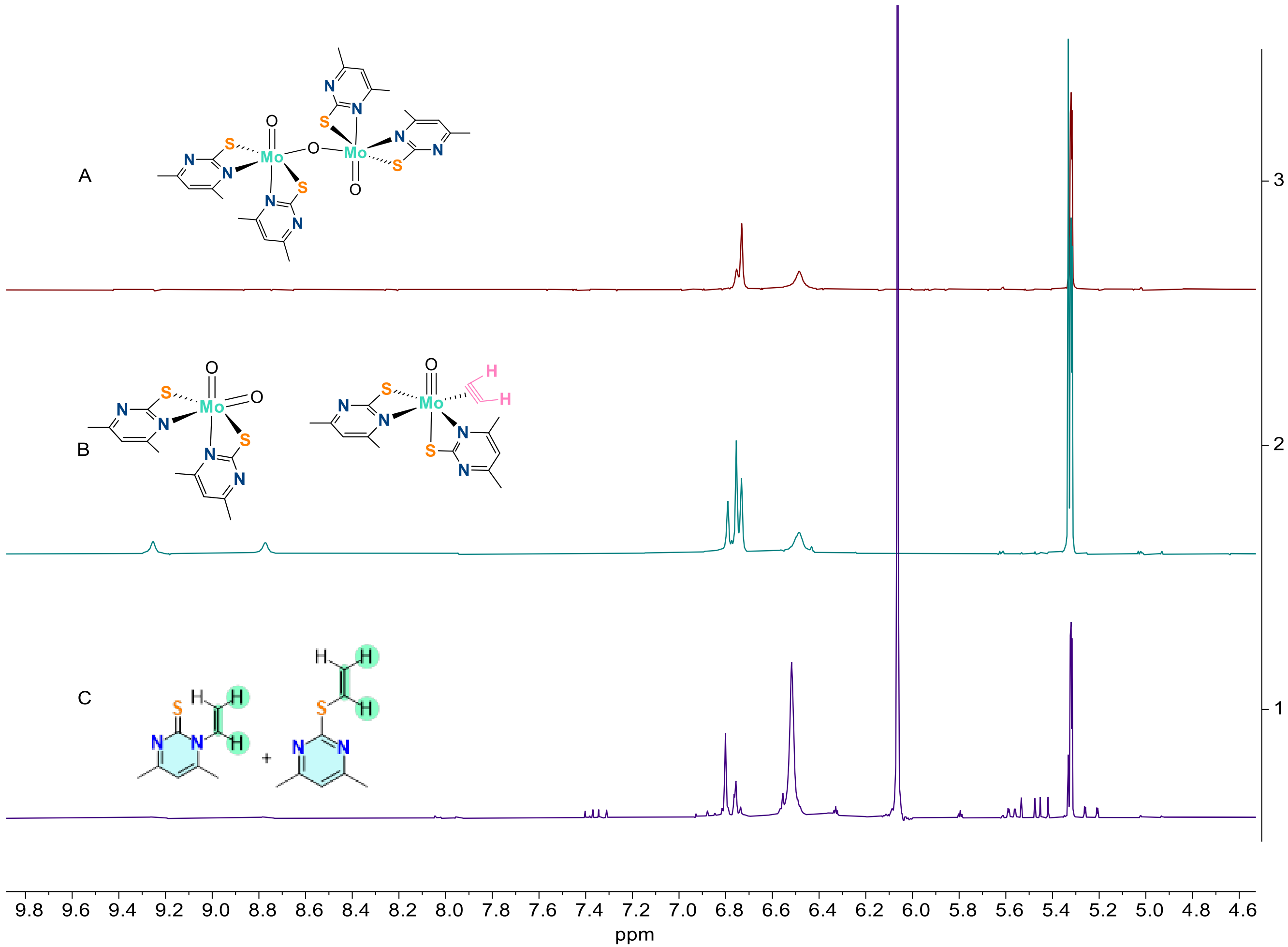

Figure S22. **A**: ^{1}H NMR spectrum of the [$Mo_2O_3(PymS)_4$] in CD_2Cl_2. **B**: ^{1}H NMR spectrum of the reaction mixture containing equimolar amounts of **Mo2** and [$MoO_2(PymS)_2$] in CD_2Cl_2, immediately upon mixing. **C**: ^{1}H NMR spectrum of the reaction mixture containing equimolar amounts of **Mo2** and [$MoO_2(PymS)_2$], 30 equiv of H_2O and trimethoxybenzene as internal standard in CD_2Cl_2.

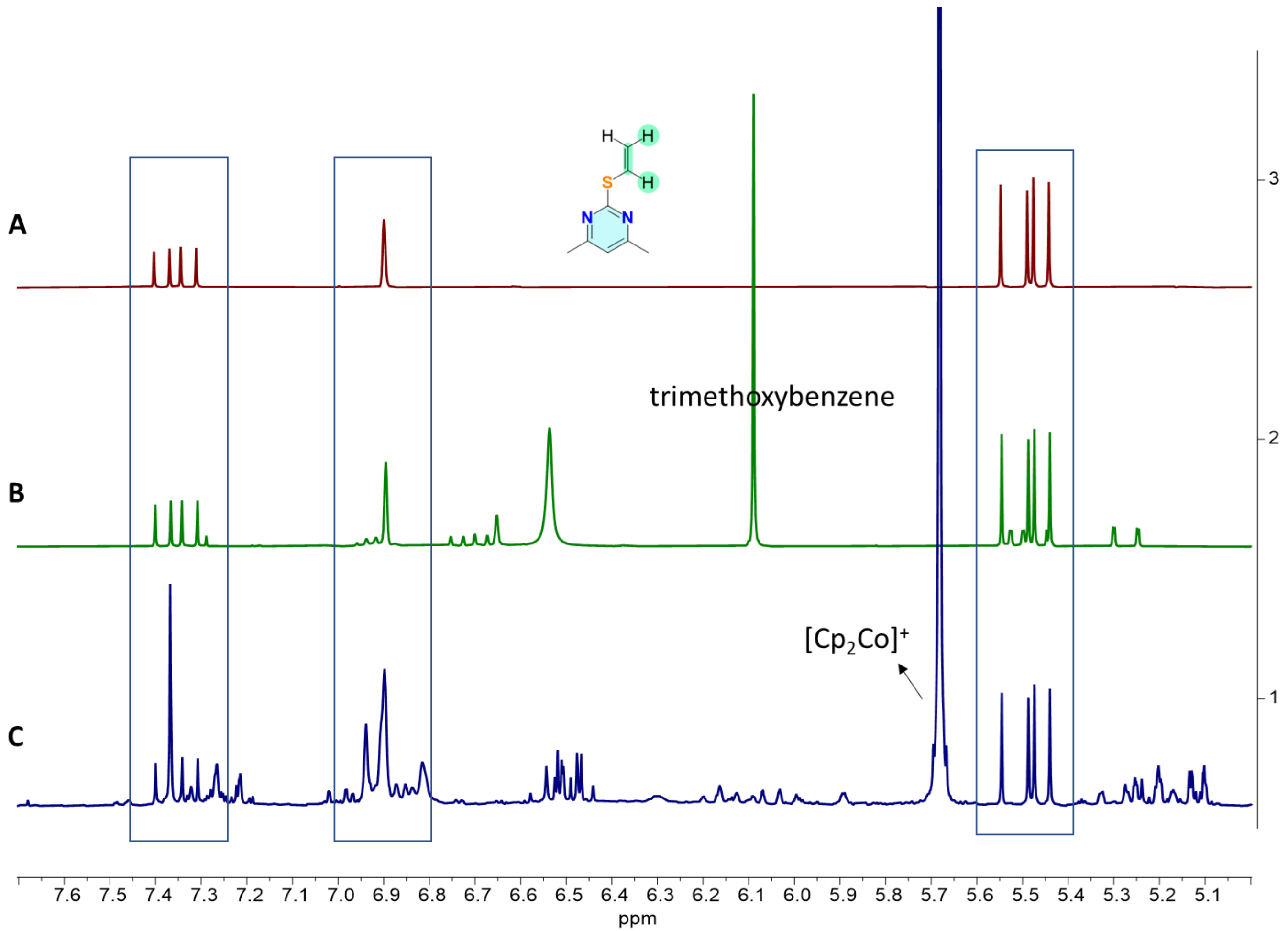

Figure S23. **A**: ^{1}H NMR spectrum of 4,6-dimethyl-2-(vinylthio)pyrimidine in CD_3CN. **B**: ^{1}H NMR spectrum of the reaction between the **Mo2** with 0.5 equiv TMAO·$2H_2O$, with additional equivalent of the PymS ligand in CD_3CN after 48 h. **C**: ^{1}H NMR spectrum of the reaction mixture containing equimolar amounts of [MoO_2(PymS)$_2$] and [Cp_2Co], and excess of water and C_2H_2 in CD_3CN after 48 h. For clarity, only the aromatic region of the spectra is shown. The peaks belonging to the 4,6-dimethyl-2-(vinylthio)pyrimidine are framed.

5 X-Ray Crystallographic Data

Crystal Structure Determination – General. The X-ray data collections for complexes **W1**, **W2, Mo1** and **Mo2** were performed on a Bruker APEX-II CCD diffractometer at 100 K using Mo Kα radiation (λ= 0.71073 Å). Absorption corrections were made semi-empirically from equivalents. The structures were solved using direct methods (SHELXS-97)[10] and refined by full-matrix least-squares techniques against F^2 (SHELXL-2014/6)[11]. The non-hydrogen atoms were refined with anisotropic displacement parameters without any constraints. The positions of the H atoms of the ethyne ligand were taken from a difference Fourier map, the C–H distances were fixed to 0.95 Å, and the H atoms were refined with common isotropic displacement parameters without any constraints to the bond angles. The H atoms of the pyrimidine rings were put at the external bisectors of the C–C–C angles at C–H distances of 0.95Å and individual isotropic displacement parameters were refined for these H atoms. The H atoms of the methyl groups were refined with common isotropic displacement parameters for the H atoms of the same group and idealized geometries with tetrahedral angles, enabling rotation around the C–C bonds, and C–H distances of 0.98 Å.

The X-ray data collections for complexes [MoO_2(PymS)$_2$] and [Mo_2O_3(PymS)$_4$] were performed on a XtaLAB Synergy, Dualflex, HyPix-Arc 100 diffractometer at 100 K using Cu Kα radiation (λ= 1.54184 Å). Data reduction, scaling and absorption corrections were performed using the *CrysAlisPro*[12] software. A numerical absorption correction based on gaussian integration over a multifaceted crystal model and an empirical absorption correction using spherical harmonics, implemented in SCALE3 ABSPACK scaling algorithm, were performed. The structures were solved using the intrinsic phasing method (SHELXT-2018/2)[13] and by using the Olex2[14] software as the graphical interface. The models were refined by full-matrix least-squares techniques against F^2 (SHELXL-

2019/3).[11] The non-hydrogen atoms were refined with anisotropic displacement parameters without any constraints. Hydrogen atom positions were calculated geometrically and refined using the riding model.

Crystal data, data collection parameters, and structure refinement details are given in Tables **S2-S4**. CCDC 2492679 (complex **W1**), 2492680 (complex **W2**), 2492681 (complex **Mo1**), 2492682 (complex **Mo2**), 2546096 ([$MoO_2(PymS)_2$]) and 2546097 ([$Mo_2O_3(PymS)_4$]) contain the supplementary crystallographic data for this paper. This data can be obtained free of charge via http://www.ccdc.cam.ac.uk/ or from Cambridge Crystallographic Data Centre, 12 Union Road, Cambridge, CB2 1EZ, UK.

Table S2. Crystallographic data and structure refinement details of **W1** and **W2**.

**Crystal data**	**W1**	**W2**
CIF data code	MB67	MB74
Empirical formula	$C_{15}H_{16}N_4OS_2W$	$C_{14}H_{16}N_4OS_2W$
Formula weight	516.29	504.28
Crystal description	block, red	needle, colourless
Crystal size	0.19 x 0.19 x 0.16 mm	0.25 x 0.11 x 0.09 mm
Radiation	Mo K_α (0.71073 Å)	Mo K_α (0.71073 Å)
Crystal system	monoclinic	monoclinic
Space group	$P2_1/c$	$C2/c$
a	14.6041(13) Å	19.236(2) Å
b	8.2934(7) Å	11.1271(13) Å
c	15.8933(12) Å	16.563(2) Å
β	117.315(4)°	109.575(5)°
Volume	1710.3(3) Å^3	3340.4(7) Å^3
Z	4	8
Calc. density	2.005 g/cm^3	2.005 g/cm^3
F(000)	992	1936
Linear absorption coefficient μ	7.006 mm^{-1}	7.172 mm^{-1}
Max. and min. transmission	1.000 and 0.555	1.000 and 0.801
Unit cell determination	4.58° < Θ < 30.74°	2.31° < Θ < 35.55°
Reflections used	6500	9248
**Data collection**		
Θ range for data collection	2.58 to 30.00°	2.15 to 35.00°
Reflections collected / unique	12318 / 5003	39148 / 7350
Significant unique reflections	4164 with I > 2σ(I)	6332 with I > 2σ(I)
R(int), R(sigma)	0.0390, 0.0499	0.0350, 0.0268
Completeness to Θ_{max}	99.9%	100.0%
**Refinement**		
Data / parameters / restraints	5003 / 225 / 2	7350 / 216 / 2
Goodness-of-fit on F^2	1.065	1.015
Final R indices [I > 2σ(I)]	R1 = 0.0301, wR2 = 0.0607	R1 = 0.0187, wR2 = 0.0403
R indices (all data)	R1 = 0.0400, wR2 = 0.0639	R1 = 0.0261, wR2 = 0.0425
Weighting scheme param. a, b	0.0073, 3.3035	0.0171, 3.2578
Largest Δ/σ in last cycle	0.002	0.003
Largest diff. peak and hole	1.796 and -1.775 e/Å^3	1.329 and -0.710 e/Å^3
**CCDC no.**	2492679	2492680

Table S3. Crystallographic data and structure refinement details of **Mo1** and **Mo2**.

**Crystal data**	**Mo1**	**Mo2**
CIF data code	ME180	ME201
Empirical formula	$C_{15}H_{16}MoN_4OS_2$	$C_{14}H_{16}N_4OS_2Mo$
Formula weight	428.38	416.37
Crystal description	needle, green	block, yellow
Crystal size	0.27 x 0.09 x 0.04 mm	0.23 x 0.15 x 0.12 mm
Radiation	Mo K_α (0.71073 Å)	Mo K_α (0.71073 Å)
Crystal system	monoclinic	monoclinic
Space group	$P2_1/c$	$C2/c$
a	14.592(4) Å	19.158(2) Å
b	8.314(2) Å	11.0957(13) Å
c	15.857(4) Å	16.497(2) Å
β	117.211(3)°	109.380(7)°
Volume	1710.8(7) $Å^3$	3308.1(7) $Å^3$
Z	4	8
Calc. density	1.663 g/cm^3	1.672 g/cm^3
F(000)	864	1680
Linear absorption coefficient μ	1.019 mm^{-1}	1.051 mm^{-1}
Max. and min. transmission	1.000 and 0.788	1.000 and 0.365
Unit cell determination	2.84° < Θ < 35.58°	2.62° < Θ < 28.60°
Reflections used	9987	4867
**Data collection**		
Θ range for data collection	2.58 to 35.00°	2.15 to 28.00°
Reflections collected / unique	27782 / 7522	11983 / 4001
Significant unique reflections	6478 with I > 2σ(I)	3122 with I > 2σ(I)
R(int), R(sigma)	0.0413, 0.0472	0.0637, 0.0651
Completeness to Θ_{max}	99.9%	99.8%
**Refinement**		
Data / parameters / restraints	7522 / 225 / 2	4001 / 216 / 2
Goodness-of-fit on F^2	1.109	1.097
Final R indices [I > 2σ(I)]	R1 = 0.0486, wR2 = 0.1415	R1 = 0.0524, wR2 = 0.1505
R indices (all data)	R1 = 0.0550, wR2 = 0.1455	R1 = 0.0693, wR2 = 0.1639
Weighting scheme param. a, b	0.0825, 2.9540	0.0989, 1.0102
Largest Δ/σ in last cycle	0.001	0.001
Largest diff. peak and hole	1.563 and -1.087 e/$Å^3$	1.607 and -1.549 e/$Å^3$
**CCDC no.**	2492681	2492682

Table S4. Crystallographic data and structure refinement details of $[MoO_2(PymS)_2]$ and $[Mo_2O_3(PymS)_4]$.

**Crystal data**	$[MoO_2(PymS)_2]$	$[Mo_2O_3(PymS)_4]$
CIF data code	AD297	AD301
Empirical formula	$C_{12}H_{14}MoN_4O_2S_2$, CH_2Cl_2	$C_{24}H_{28}Mo_2N_8O_3S_4$
Formula weight	491.26	796.66
Crystal description	block, yellow	block, purple
Crystal size	0.16 x 0.13 x 0.10 mm	0.13 x 0.11 x 0.08 mm
Radiation	Cu K_α (1.54184 Å)	Cu K_α (1.54184 Å)
Crystal system	tetragonal	monoclinic
Space group	$I4_1cd$	$C2/c$
a	22.7554(2)	19.2271(2) Å
b	22.7554(2)	10.96100(10) Å
c	14.4508(2)	14.52380(10) Å
β	90°	93.5510(10)°
Volume	7482.74(17)°	3054.99(5) $Å^3$
Z	16	4
Calc. density	1.744 g/cm^3	1.732 g/cm^3
F(000)	3936	1600
Linear absorption coefficient μ	10.584 mm^{-1}	9.630 mm^{-1}
Max. and min. transmission	1.000 and 0.77	0.949 and 0.537
Unit cell determination	3.85° < Θ < 77.43°	4.61° < Θ < 77.36°
Reflections used	21709	12862
**Data collection**		
Θ range for data collection	3.89 to 77.37°	4.61 to 77.36°
Reflections collected / unique	37628 / 3975	20117 / 3238
Significant unique reflections	3867 with I > 2σ(I)	3122 with I > 2σ(I)
R(int), R(sigma)	0.0449, 0.0235	0.0430, 0.0262
Completeness to Θ_{max}	99.9%	99.9%
**Refinement**		
Data / parameters / restraints	3975 / 221 / 1	3238 / 191 / 0
Goodness-of-fit on F^2	1.086	1.086
Final R indices [I > 2σ(I)]	R1 = 0.0292, wR2 = 0.0786	R1 = 0.0227, wR2 = 0.0584
R indices (all data)	R1 = 0.0301, wR2 = 0.0791	R1 = 0.0236, wR2 = 0.0589
Weighting scheme param. a, b	0.051, 9.6548	0.0262, 4.8815
Largest Δ/σ in last cycle	0.001	0.001
Largest diff. peak and hole	0.54 and -0.60 $e/Å^3$	0.36 and -0.57 $e/Å^3$
**CCDC no.**	2546096	2546097

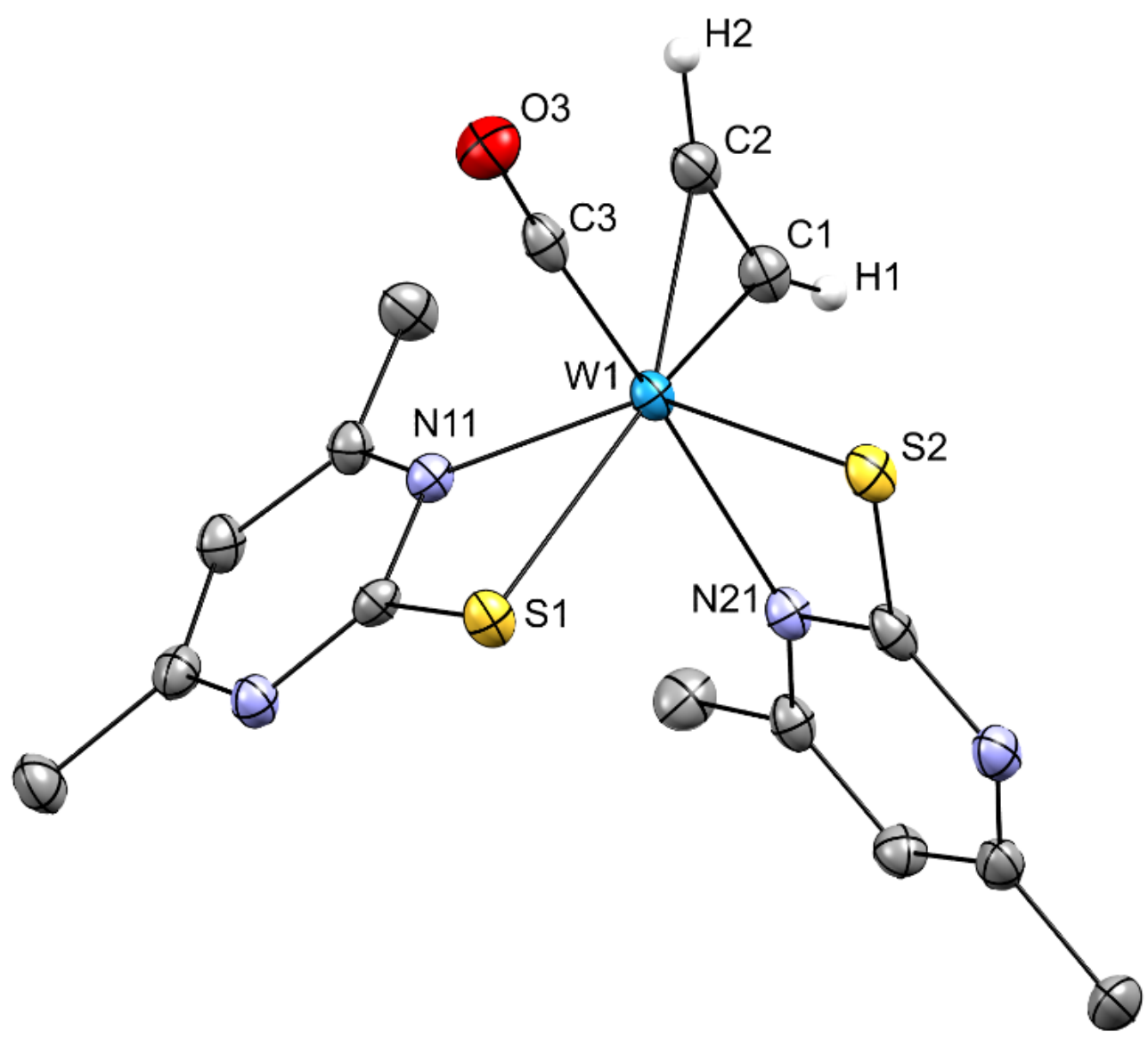

Figure S24. Molecular structure of **W1**. The probability ellipsoids are drawn at the 50% probability level. Hydrogen atoms are omitted for clarity, except those of the ethyne ligand.

Table S5. Selected bond lengths [Å] and angles [°] for **W1**.

W1–C1	2.020(3)	N11–W1–S2	144.86(8)
W1–C2	2.040(3)	C2–C1–H1	147.5(9)
W1–C3	1.966(4)	C1–C2–H2	141.9(6)
W1–N11	2.198(3)	O3–C3–W1	176.8(3)
W1–N21	2.249(3)	C12–S1–W1	80.35(13)
W1–S1	2.5925(10)	C16–N11–C12	118.0(3)
W1–S2	2.4065(10)	C16–N11–W1	137.3(2)
C1–C2	1.309(4)	C12–N11–W1	104.3(2)
C3–O3	1.153(5)	C22–S2–W1	83.59(13)
S1–C12	1.719(4)	C26–N21–C22	117.9(3)
S2–C22	1.753(4)	C26–N21–W1	142.7(3)
		C22–N21–W1	99.3(2)
C1–W1–S1	160.17(11)		
C2–W1–S1	155.50(8)	C1–C2–W1–C3	-179.9(11)
C3–W1–N21	160.56(14)	C2–C1–W1–C3	0.1(8)

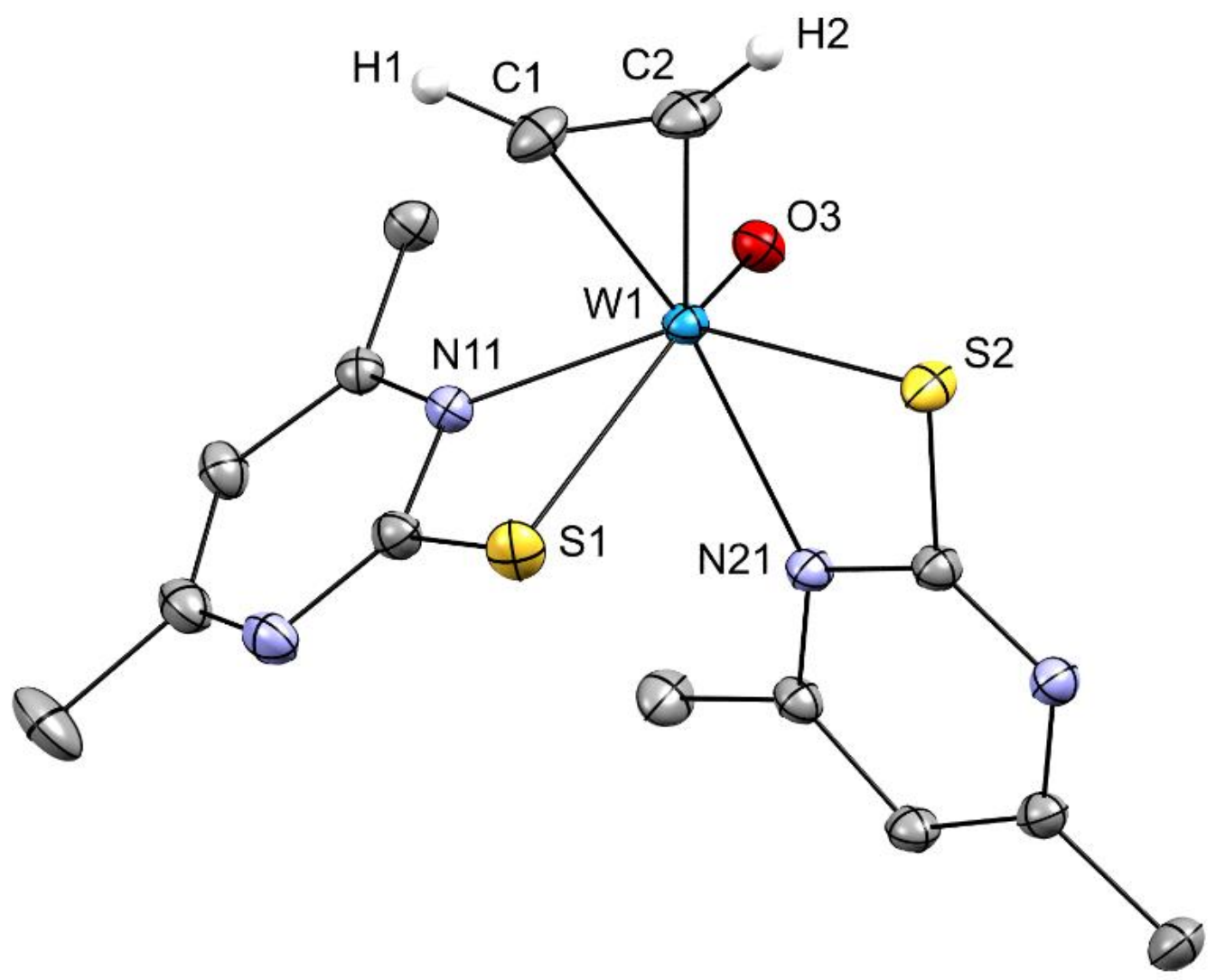

Figure S25. Molecular structure of **W2**. The probability ellipsoids are drawn at the 50% probability level. Hydrogen atoms are omitted for clarity, except those of the ethyne ligand.

Table S6. Selected bond lengths [Å] and angles [°] for **W2**.

W1–C1	2.0911(17)	N11–W1–N21	90.98(5)
W1–C2	2.0810(16)	S1–W1–S2	89.046(17)
W1–O3	1.7178(14)	C12–S1–W1	78.80(6)
W1–N11	2.1926(14)	C16–N11–C12	119.18(14)
W1–N21	2.2714(14)	C16–N11–W1	135.25(12)
W1–S1	2.6567(6)	C12–N11–W1	105.46(11)
W1–S2	2.4176(5)	C22–S2–W1	84.11(6)
C1–C2	1.276(3)	C26–N21–C22	118.22(14)
S1–C12	1.7260(18)	C26–N21–W1	142.19(11)
S2–C22	1.7465(17)	C22–N21–W1	99.58(10)
C1–W1–N21	165.74(7)	C1–C2–W1–O3	99.37(14)
C2–W1–N21	146.85(6)	C2–C1–W1–O3	-89.73(14)
O3–W1–S1	150.81(4)	C1–C2–W1–N11	-2.25(18)
N11–W1–S2	148.17(4)	C2–C1–W1–N11	178.02(11)

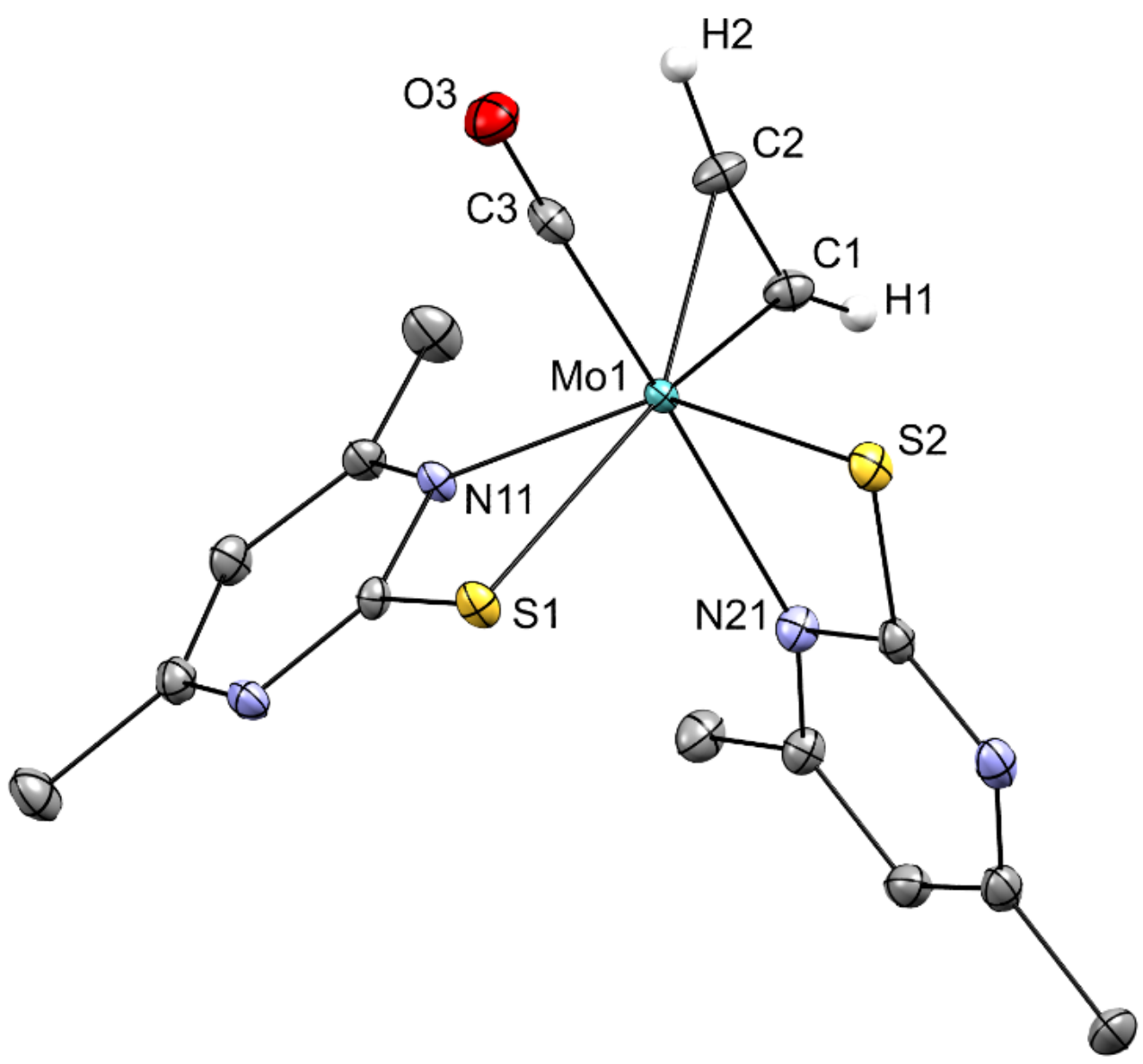

Figure S26. Molecular structure of **Mo1**. The probability ellipsoids are drawn at the 50% probability level. Hydrogen atoms are omitted for clarity, except those of the ethyne ligand.

Table S7. Selected bond lengths [Å] and angles [°] for **Mo1**.

Mo1-C1	2.029(2)	C3-Mo1-N21	161.43(10)
Mo1-C2	2.046(2)	N11-Mo1-S2	145.05(6)
Mo1-C3	1.972(3)	C2-C1-H1	144.2(8)
Mo1-N11	2.212(2)	C1-C2-H2	154.9(7)
Mo1-N21	2.251(2)	O3-C3-Mo1	178.0(2)
Mo1-S1	2.5992(9)	C12-S1-Mo1	79.90(8)
Mo1-S2	2.4048(8)	C16-N11-C12	118.5(2)
C1-C2	1.298(3)	C16-N11-Mo1	137.47(16)
C3-O3	1.138(3)	C12-N11-Mo1	103.65(15)
S1-C12	1.726(2)	C22-S2-Mo1	83.47(8)
S2-C22	1.761(3)	C26-N21-C22	118.0(2)
C1-Mo1-S1	159.95(8)	C26-N21-Mo1	142.57(18)
C2-Mo1-S1	155.75(6)	C22-N21-Mo1	99.29(16)
		C1-C2-Mo1-C3	-179.1(2)

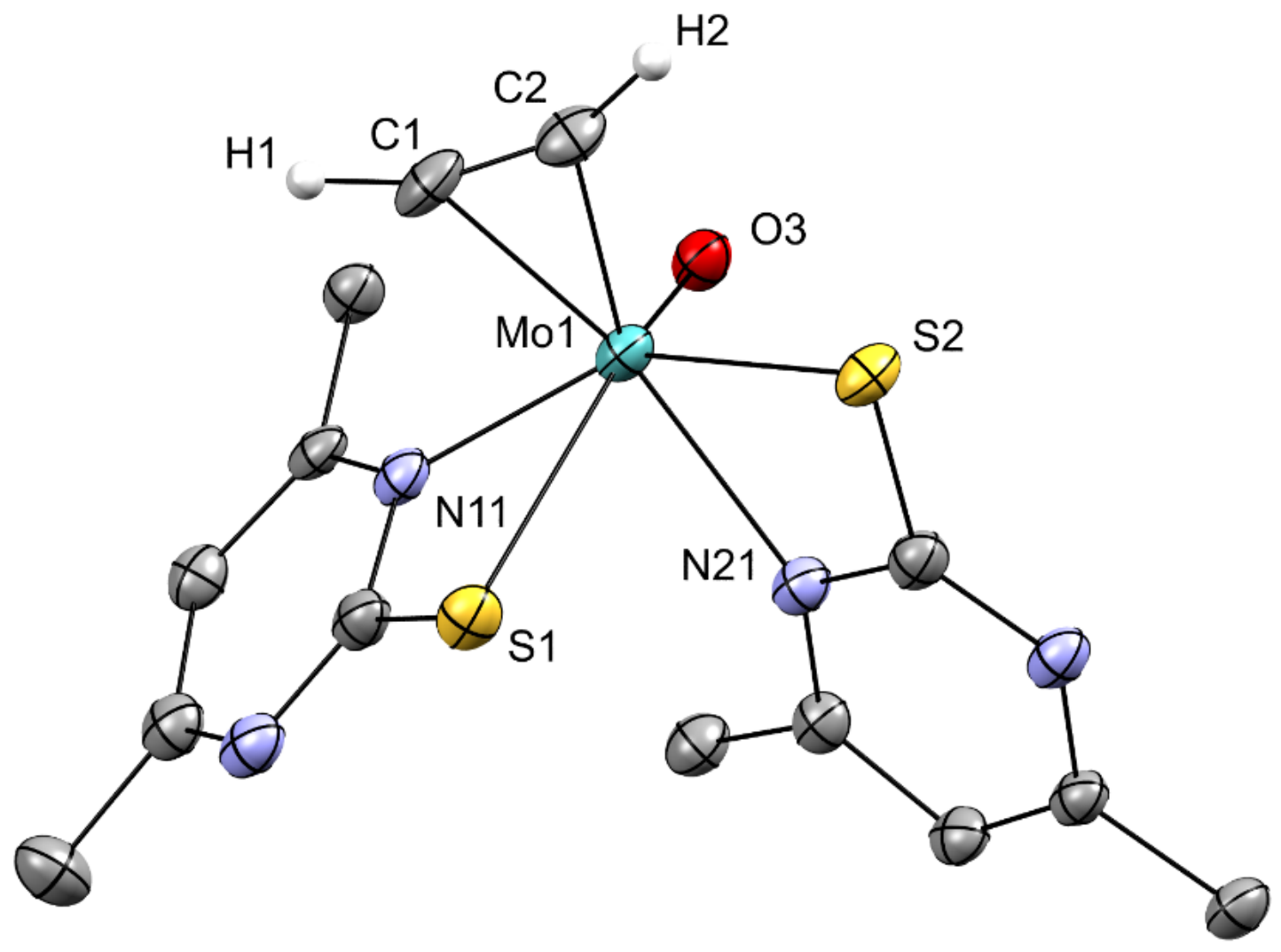

Figure S27. Molecular structure of **Mo2**. The probability ellipsoids are drawn at the 40% probability level. Hydrogen atoms are omitted for clarity, except those of the ethyne ligand.

Table S8. Selected bond lengths [Å] and angles [°] for **Mo2**.

Mo1-C1	2.108(3)	N11-Mo1-N21	92.53(12)
Mo1-C2	2.109(4)	S1-Mo1-S2	89.61(4)
Mo1-O3	1.688(3)	C12-S1-Mo1	79.09(16)
Mo1-N11	2.204(4)	C16-N11-C12	119.3(4)
Mo1-N21	2.274(3)	C16-N11-Mo1	135.5(3)
Mo1-S1	2.6652(12)	C12-N11-Mo1	104.9(3)
Mo1-S2	2.4141(11)	C22-S2-Mo1	83.48(15)
C1-C2	1.252(5)	C26-N21-C22	118.9(3)
S1-C12	1.714(5)	C26-N21-Mo1	142.2(3)
S2-C22	1.748(4)	C22-N21-Mo1	98.9(3)
C1-Mo1-N21	165.07(13)	C1-C2-Mo1-O3	98.8(3)
C2-Mo1-N21	146.84(14)	C2-C1-Mo1-O3	-90.2(3)
O3-Mo1-S1	150.79(12)	C1-C2-Mo1-N11	-2.4(3)
N11-Mo1-S2	149.38(9)	C2-C1-Mo1-N11	177.8(3)

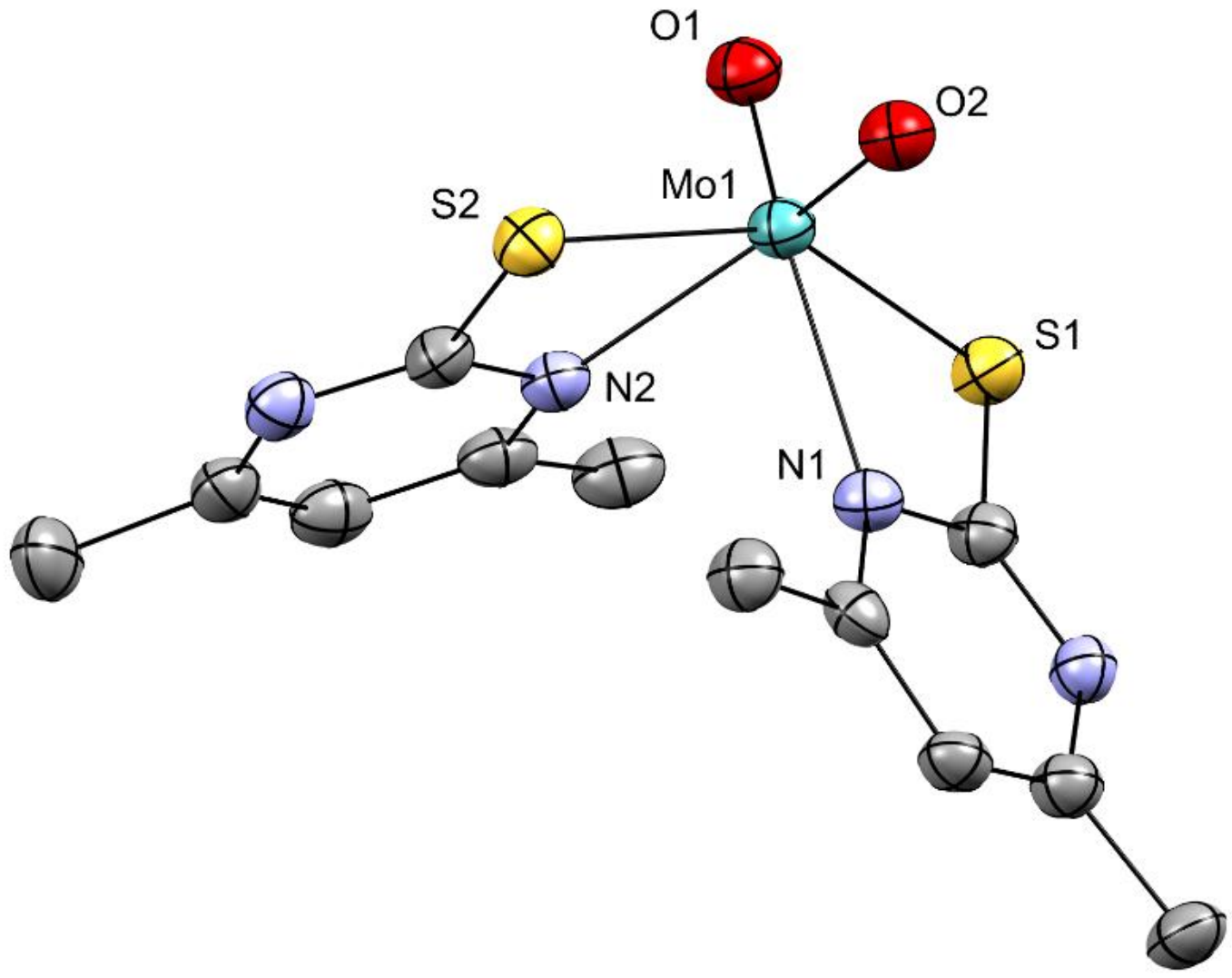

Figure S28. Molecular structure of [$MoO_2(PymS)_2$] with ellipsoids at 50% probability. Hydrogen atoms and the solvent molecule are omitted for clarity.

Table S9. Selected bond lengths [Å] and angles [°] for [$MoO_2(PymS)_2$].

Mo1-O1	1.701(4)	O1-Mo1-N1	155.73(16)
Mo1-O2	1.697(4)	O1-Mo1-N2	89.06(17)
Mo1-S1	2.4350(12)	O2-Mo1-S1	105.90(15)
Mo1-S2	2.4361(13)	O2-Mo1-S2	91.53(15)
Mo1-N1	2.356(4)	O2-Mo1-N1	90.30(17)
Mo1-N2	2.320(5)	O2-Mo1-N2	155.33(19)
		N1-Mo1-S1	64.96(10)
S1-Mo1-S2	149.55(5)	N1-Mo1-S2	90.74(11)
O1-Mo1-O2	106.02(19)	N2-Mo1-S1	92.41(12)
O1-Mo1-S1	92.87(12)	N2-Mo1-S2	65.14(12)
O1-Mo1-S2	106.31(13)	N1-Mo1-N2	82.50(15)

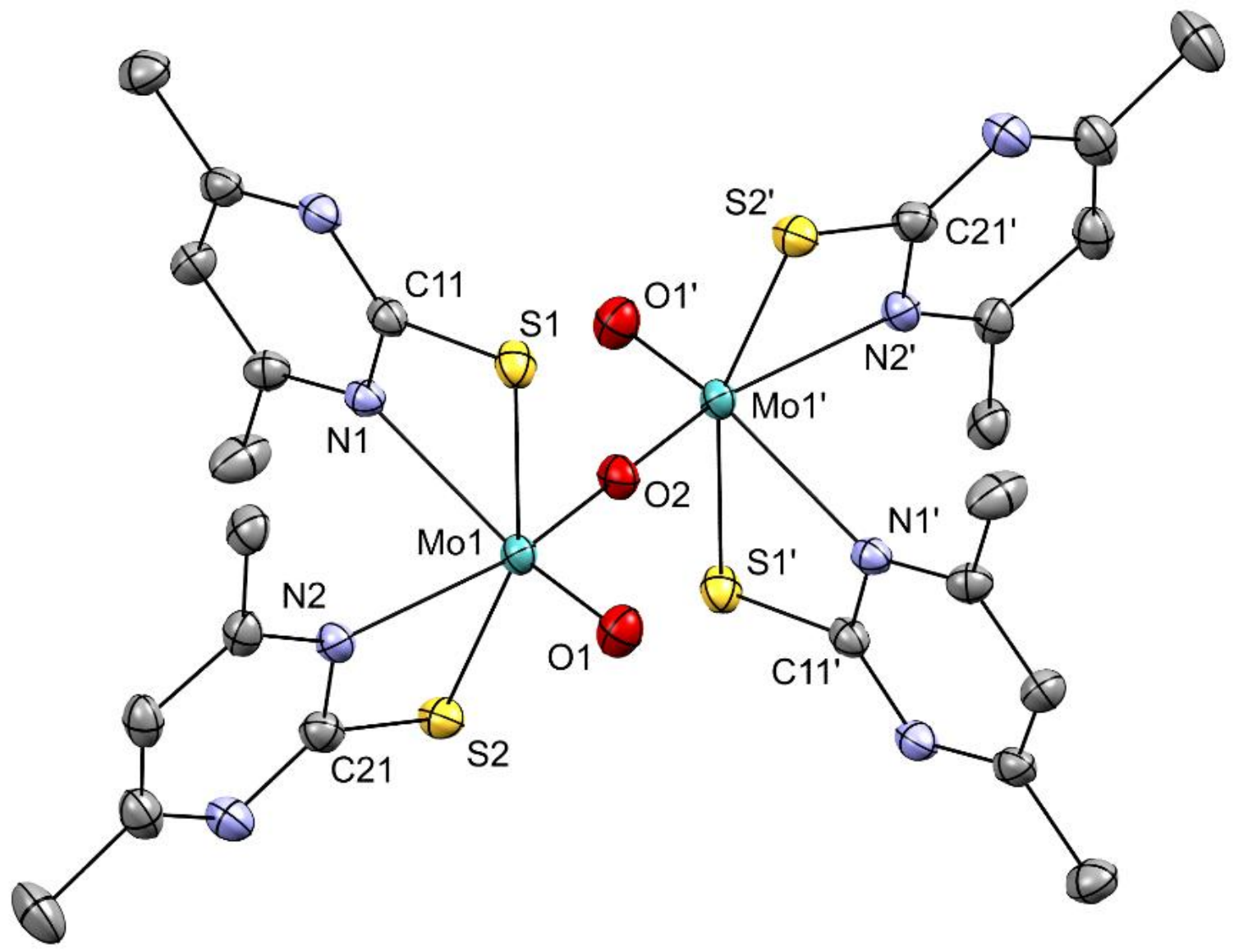

Figure S29. Molecular structure of [Mo_2O_3(PymS)$_4$] with ellipsoids at 50% probability. Hydrogen atoms are omitted for clarity. Symmetry used to generate equivalent atoms: 1/2-x, 1/2-y, 1-z

Table S10. Selected bond lengths [Å] and angles [°] for [Mo_2O_3(PymS)$_4$].

Mo1-O1	1.6791(16)	O1-Mo1-N1	154.10(7)
Mo1-O2	1.85017(15)	O1-Mo1-N2	99.81(7)
Mo1-S1	2.4914(5)	O2-Mo1-S1	94.074(14)
Mo1-S2	2.4365(5)	O2-Mo1-S2	93.558(13)
Mo1-N1	2.3611(16)	O2-Mo1-N1	85.83(4)
Mo1-N2	2.2301(17)	O2-Mo1-N2	150.93(4)
		N1-Mo1-S1	64.08(4)
Mo1-O2-Mo1′	180	N1-Mo1-S2	97.27(4)
S1-Mo1-S2	159.214(18)	N2-Mo1-S1	98.44(5)
O1-Mo1-O2	105.97(5)	N2-Mo1-S2	66.64(5)
O1-Mo1-S1	91.68(6)	N1-Mo1-N2	76.39(6)
O1-Mo1-S2	104.73(6)		

6 Electron Paramagnetic Resonance

Room-temperature EPR spectra were recorded on an X-band Bruker EMX EPR spectrometer. Samples were measured in solution (CH_2Cl_2, exclusion of oxygen) under identical instrumental conditions. Each EPR spectrum is the result of 250 scans.

Three reaction mixture were followed by EPR spectroscopy over 72 h:

(**1**) Solution of **Mo2** (24 µmol, 1equiv) in CH_2Cl_2 (2.5 mL) mixed with TMAO·$2H_2O$ (12 µmol, 0.5 equiv); After stirring for 5 min, an 0.3 mL aliquot has been submitted in J. Young tube for the EPR measurement (Figure S30).

(**2**) Solution of **Mo2** (24 µmol, 1equiv) and [MoO_2(PymS)$_2$] (24 µmol, 1equiv) was prepared in CH_2Cl_2 (3 mL). To a 0.3 mL aliquot of this mixture, water (70 µL) was added and the sample has been submitted in J. Young tube for the EPR measurement (Figure S31)

(**3**)) Solution of **W2** (24 µmol, 1equiv) in CH_2Cl_2 (2.5 mL) mixed with TMAO·$2H_2O$ (12 µmol, 0.5 equiv); After stirring for 5 min, an 0.3 mL aliquot has been submitted in J. Young tube for the EPR measurement

For both reaction mixtures with **Mo2**, EPR spectra was recorded upon mixing and the pattern is consistent with hyperfine coupling of an S=1/2 paramagnetic center to a nucleus with I=5/2, as expected for $^{95/97}$Mo in Mo(V)-centered species.[15,16]

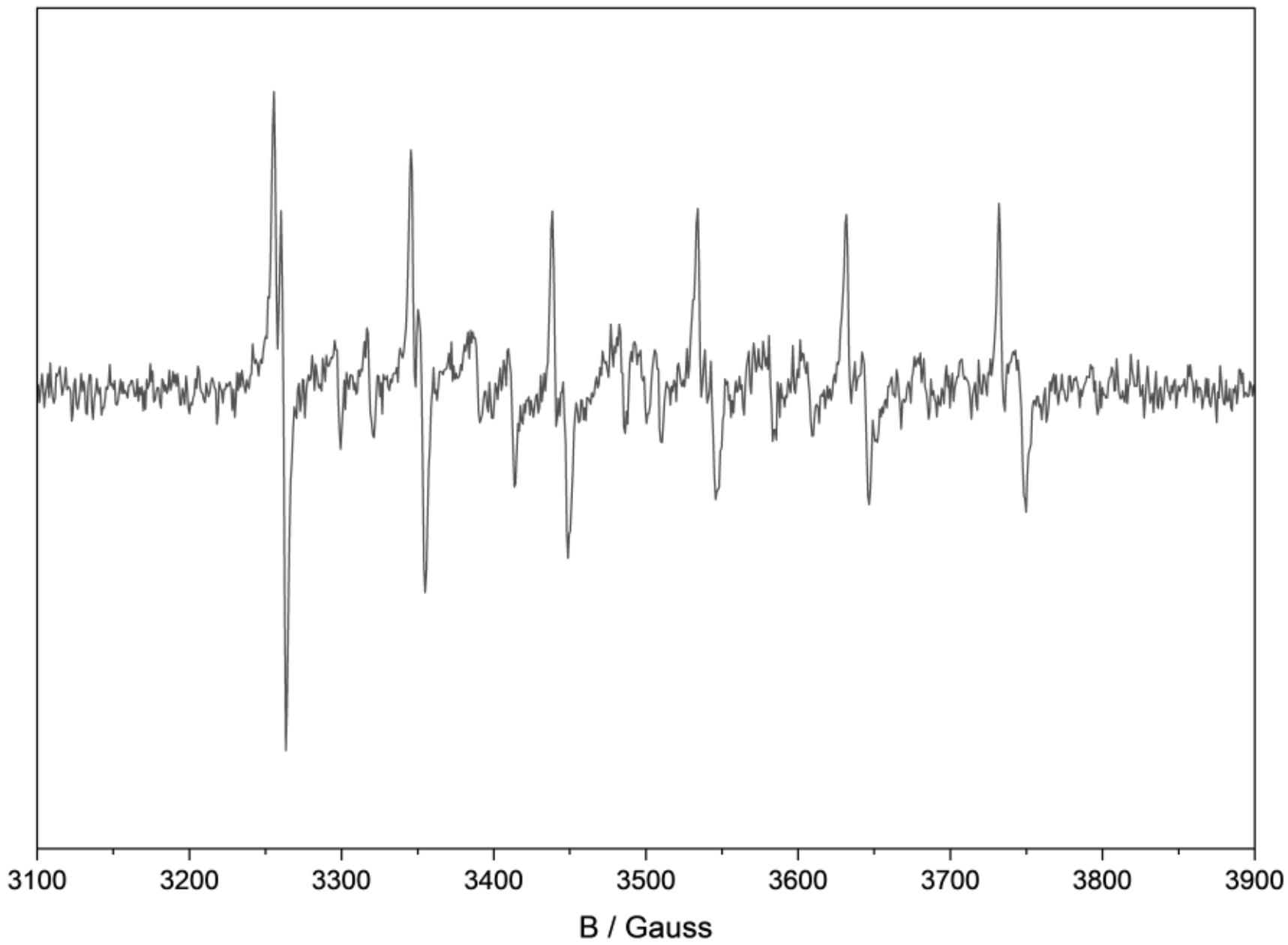

Figure S30. EPR spectrum of the reaction mixture containg **Mo2** (20 µmol, 1equiv) and TMAO·2H_2O (10 µmol, 0.5 equiv) in CH_2Cl_2 (5 mL)

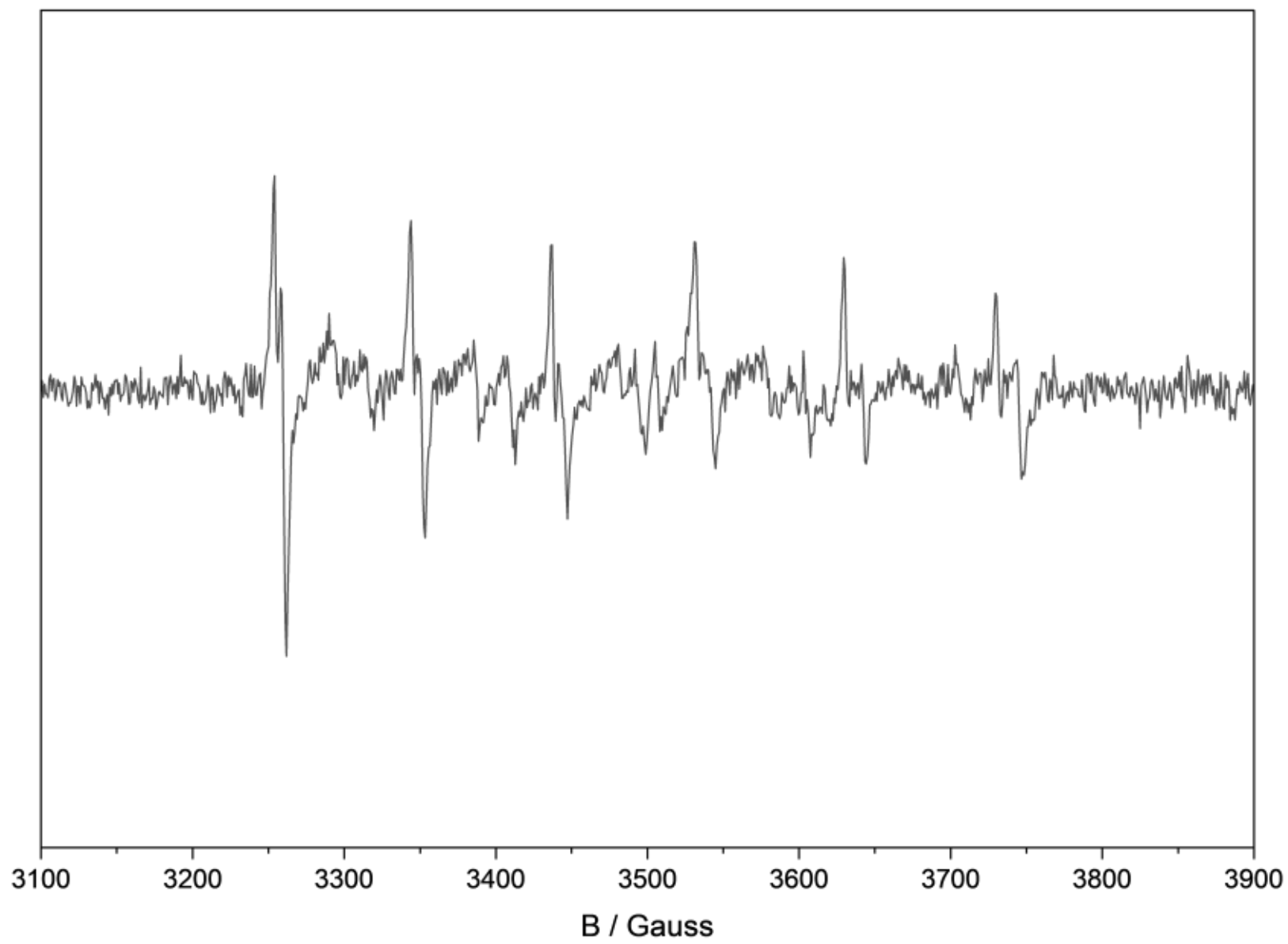

Figure S31. EPR spectrum of the reaction mixture containg **Mo2** (20 µmol, 1 equiv), [MoO_2(PymS)$_2$] (20 µmol, 1equiv) and water (20 µL, 1 equiv) in CH_2Cl_2 (5 mL)

The spectra are very weak, but reproducible for both samples. No significant changes in the EPR spectra were observed over a time span of approximately 72 hours, suggesting that the concentration and nature of the detected paramagnetic species remain largely unchanged under these conditions. The unequal line intensities and minor additional features suggest that the

spectra may contain contributions from more than one Mo(V)-type species. In line with this, we were not able to reproduce the experimental spectra satisfactorily using a simulation model containing only one Mo(V) species. Given the limitations in signal quality and the challenges of quantitatively comparing weak EPR spectra, these observations should be interpreted with caution and cannot be considered definitive at this stage.

Overall, the EPR data are consistent with the presence of Mo(V)-centered paramagnetic species in both samples. However, the spectra alone do not allow us to distinguish unambiguously between a single Mo(V)-type species with unresolved anisotropy, two overlapping Mo(V)-type species, or non-equivalent Mo environments within the binuclear complex.

In contrast, EPR spectra for the reaction (**3**) of **W2** and 0.5 equivalents TMAO·$2H_2O$ revealed no radicals.

7 Cyclic Voltammetry

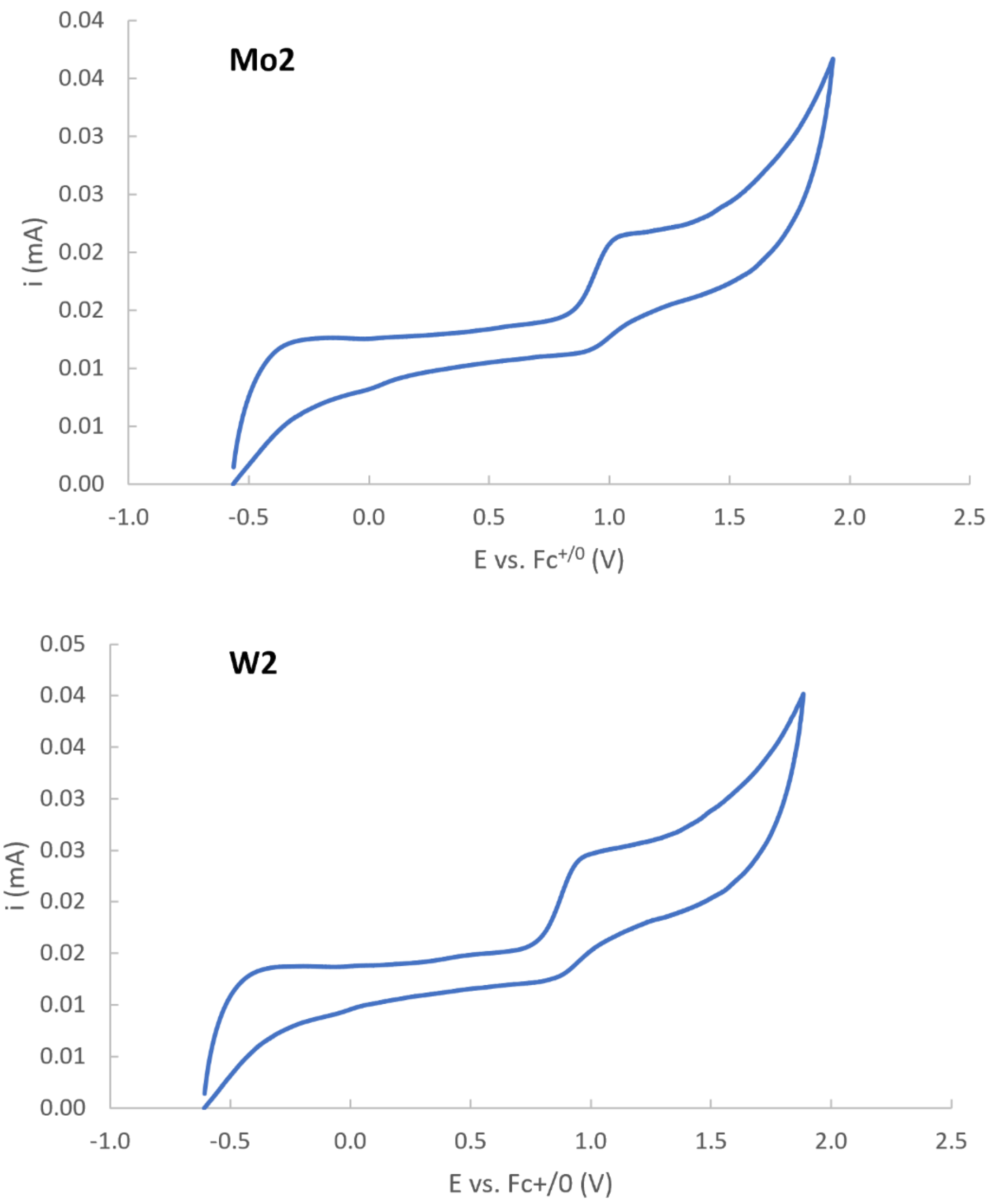

Figure S32. Cyclic voltammograms of a 1 mM solution complexes **Mo2** (top) and **W2** (bottom) in MeCN (0.1 M *n*Bu_4NPF_6 electrolyte) at 100 mV/s scan rate.

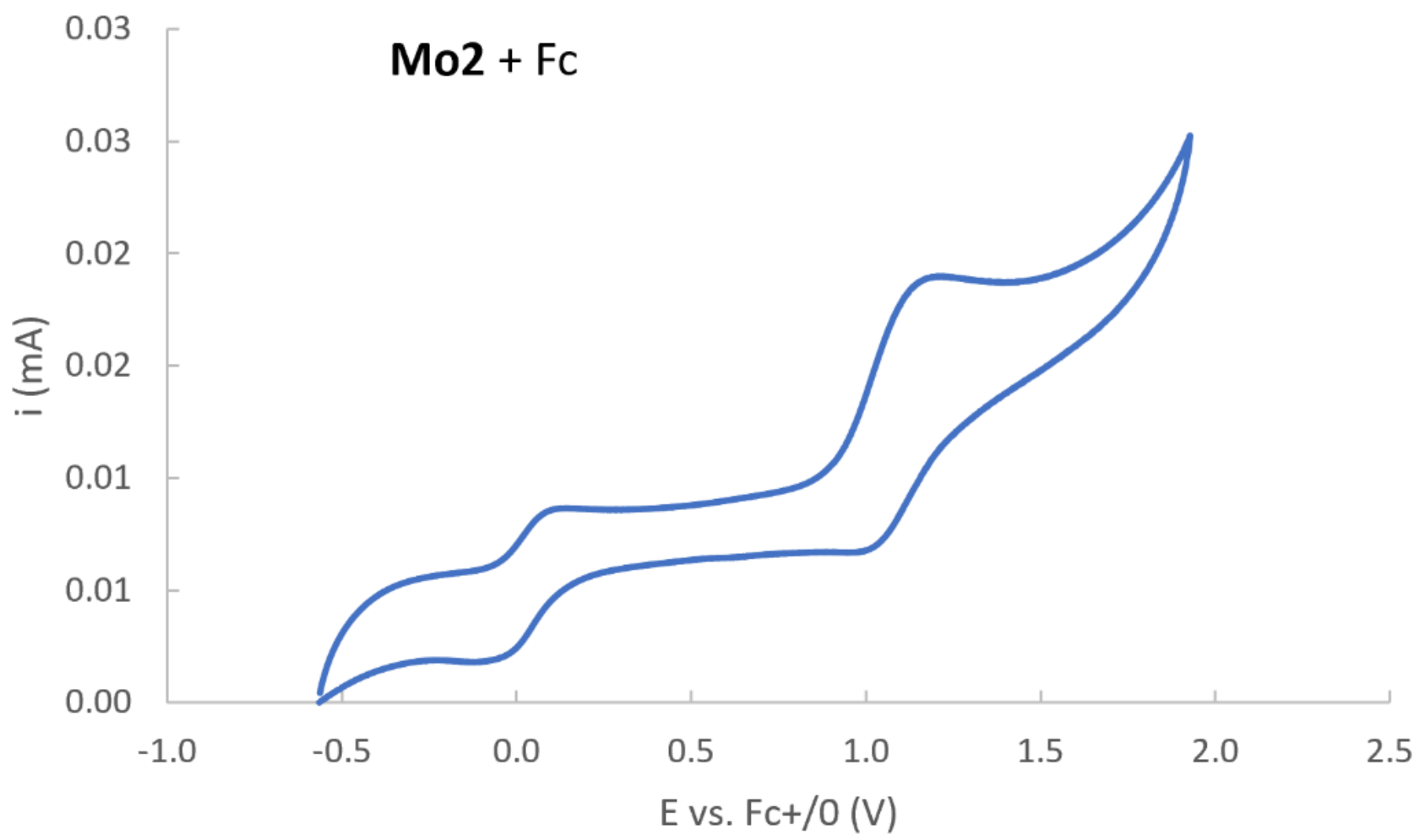

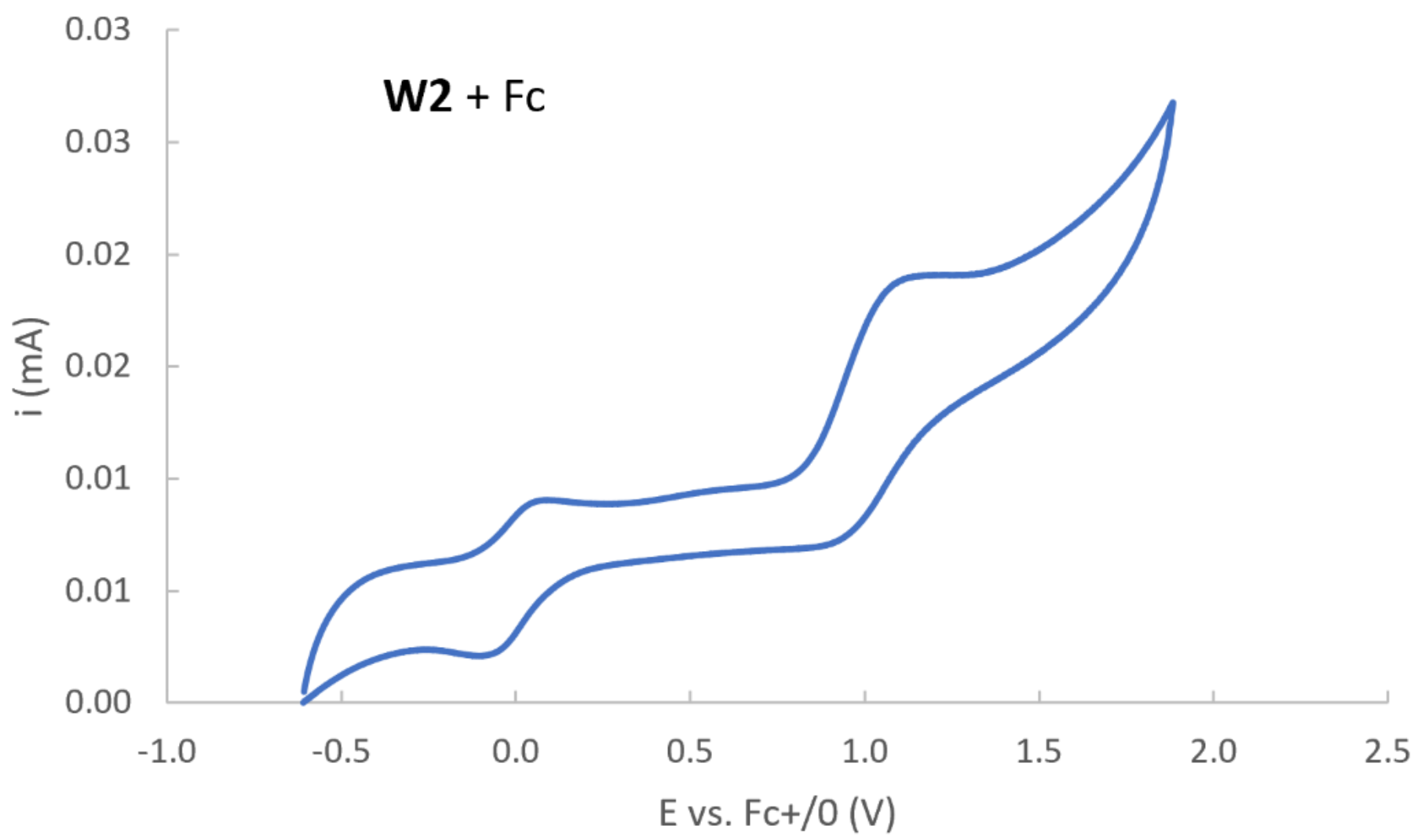

Figure S33. Top: cyclic voltammogram of 1 mM **Mo2** and 1 mM Fc solution in MeCN (0.1 M nBu$_4$NPF$_6$ electrolyte) at 100 mV/s scan rate. Bottom: cyclic voltammogram of 1 mM **W2** and 1 mM Fc solution in MeCN (0.1 M nBu$_4$NPF$_6$ electrolyte) at 100 mV/s scan rate.

The number of electrons exchanged in a single oxidation step was determined using the Randles–Ševčík equation (equation 1).[17] Cyclic voltammetry measurements were performed on equimolar solutions of the complexes and ferrocene (both 1mM in MeCN (0.1 M nBu$_4$NPF$_6$ electrolyte) at 100 mV/s scan rate), allowing direct comparison of their peak current intensities. Diffusion coefficients for **Mo2**, **W2**, and ferrocene (Fc) were obtained by Diffusion-Ordered (DOSY) NMR spectroscopy under the same conditions, enabling calculation of number of the exchanged electrons (equation 2).

$$i = 0.4463Ac\sqrt{\frac{{n_e}^3F^3vD}{RT}} \qquad \text{equation 1}$$

i: forward peak-current (A), A: electrode surface area (cm²); c: bulk concentration of the analyte (mol/cm³); n_e: number of electrons transferred in the redox event; F: Faraday's constant (C/mol); v: scan rate (V/s); D_0: diffusion coefficient of the oxidized analyte (cm²/s); R: ideal gas constant (J/(mol·K)); T: absolute temperature (K).

$$n_e = \left(\frac{i_{complex}}{i_{Fc}} \cdot \sqrt{\frac{D_{Fc}}{D_{complex}}}\right)^{\frac{2}{3}} \qquad \text{equation 2}$$

Table S11. Calculation of the number of exchanged electrons (n_e) from Randles–Ševčík equation.

Complex	**E_{ox} vs. Fc (V)**	**$i_{complex}/i_{Fc}$**	**$D_{Fc}/D_{complex}$**	**n_e**
**Mo2**	1.03	2.236	1.675	2.03 ≈ **2**
**W2**	0.98	2.120	1.664	1.96 ≈ **2**

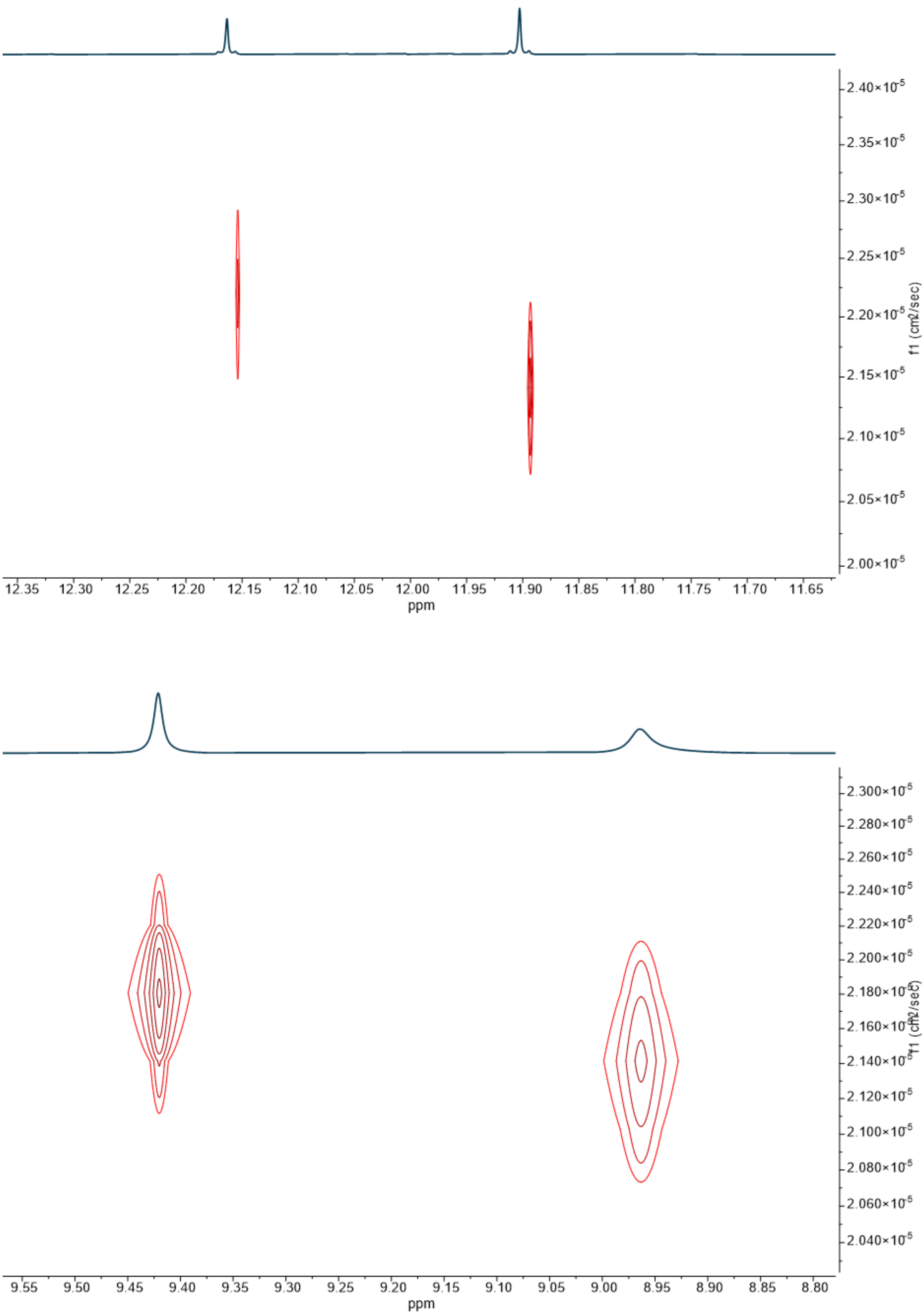

Figure S34. DOSY NMR spectra of **W2** (top) and **Mo2** (bottom) in CD_3CN. Only acetylenic region is shown for clarity.

8 Computational Data

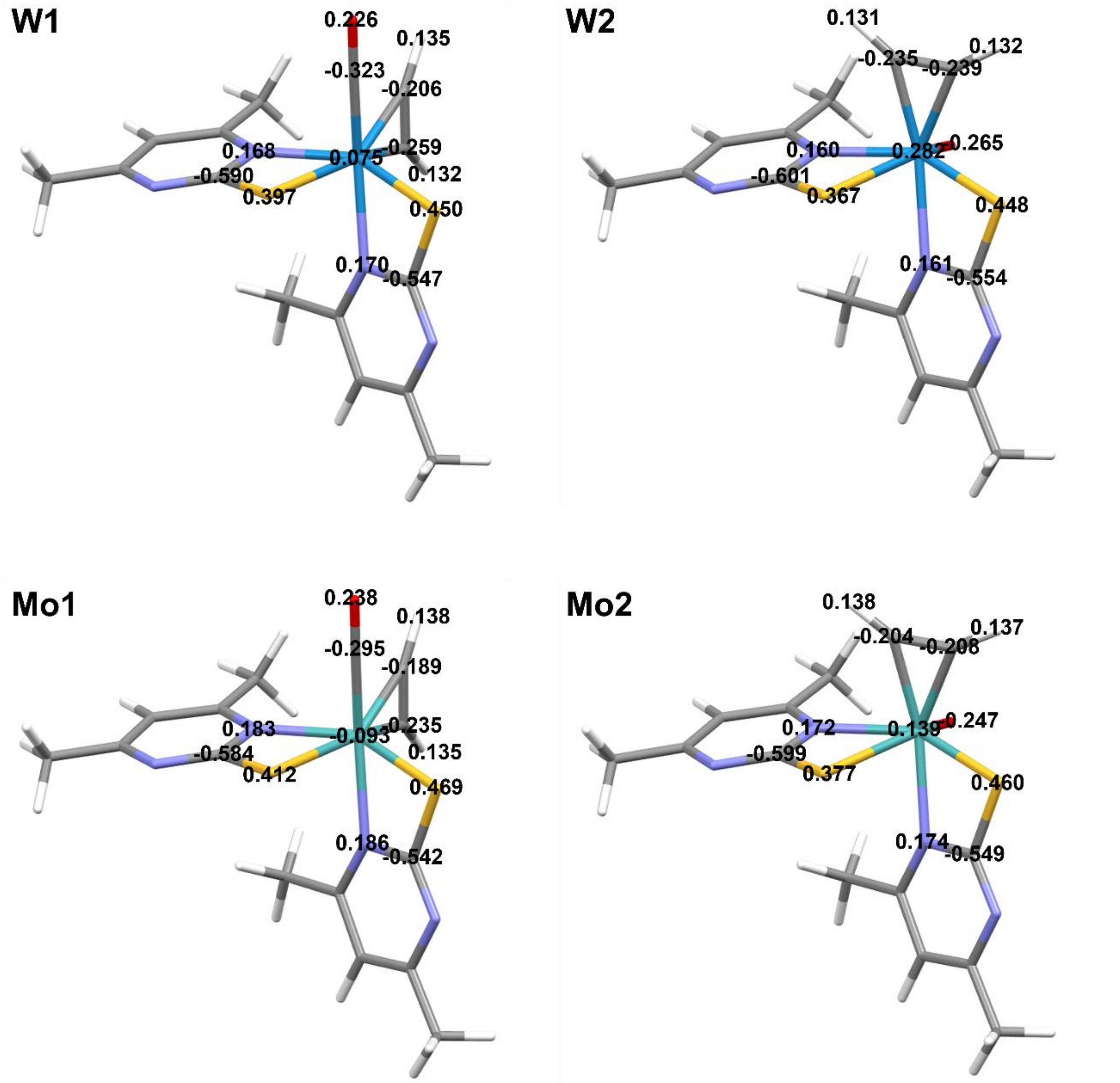

Figure S35. Optimized structures for all four investigated complexes. Löwdin charges of selected atoms have been depicted.

Table S12. Comparison of bond lengths obtained from XRD analysis and DFT-optimized structures. Only the first coordination sphere is considered: M–C, M–N and M–S bonds (M = Mo or W).

	W1		W2		Mo1		Mo2	
bonds	XRD	DFT	XRD	DFT	XRD	DFT	XRD	DFT
M-C	2.020	2.024	2.091	2.082	2.029	2.016	2.108	2.078
M-C	2.040	2.036	2.081	2.073	2.046	2.029	2.109	2.081
M-N	2.198	2.193	2.193	2.202	2.212	2.201	2.204	2.207
M-N	2.249	2.245	2.271	2.270	2.251	2.241	2.274	2.257
M-S	2.592	2.579	2.657	2.637	2.600	2.570	2.665	2.637
M-S	2.406	2.401	2.418	2.423	2.405	2.383	2.414	2.408

Table S13. Löwdin charges of atoms in **Mo1**.

0 Mo: -0.092514	13 H : 0.152286	26 N : 0.163872
1 C : -0.234662	14 C : -0.222121	27 C : -0.225336
2 H : 0.135128	15 C : -0.250309	28 C : -0.117220
3 C : -0.188873	16 H : 0.140174	29 H : 0.153087
4 H : 0.138491	17 H : 0.127334	30 C : -0.211735
5 C : -0.294906	18 H : 0.142551	31 C : -0.248544
6 O : 0.237825	19 C : -0.238496	32 H : 0.141824
7 S : 0.412187	20 H : 0.146828	33 H : 0.127984
8 N : 0.183121	21 H : 0.134092	34 H : 0.143243
9 C : -0.584426	22 H : 0.140044	35 C : -0.236587
10 N : 0.158461	23 S : 0.469166	36 H : 0.150422
11 C : -0.239241	24 N : 0.186004	37 H : 0.133827
12 C : -0.130816	25 C : -0.541805	38 H : 0.139639

Table S14. Löwdin charges of atoms in **Mo2**.

0 Mo: 0.139097	13 C : -0.219523	26 C : -0.221317
1 C : -0.203769	14 C : -0.249753	27 C : -0.112743
2 H : 0.137690	15 H : 0.140407	28 H : 0.154083
3 C : -0.208131	16 H : 0.127552	29 C : -0.205125
4 H : 0.137328	17 H : 0.142446	30 C : -0.247197
5 O : -0.246696	18 C : -0.241077	31 H : 0.142722
6 S : 0.377506	19 H : 0.147865	32 H : 0.128570
7 N : 0.171783	20 H : 0.131774	33 H : 0.143602
8 C : -0.598807	21 H : 0.146400	34 C : -0.234543
9 N : 0.154520	22 S : 0.459744	35 H : 0.151246
10 C : -0.238975	23 N : 0.174483	36 H : 0.134522
11 C : -0.129900	24 C : -0.549445	37 H : 0.146314
12 H : 0.152658	25 N : 0.164692	

Table S15. Löwdin charges of atoms in **W1**.

0 W :	0.075336	13 H :	0.152594	26 N :	0.163631
1 C :	-0.258862	14 C :	-0.222011	27 C :	-0.225403
2 H :	0.131521	15 C :	-0.250594	28 C :	-0.117655
3 C :	-0.206340	16 H :	0.140066	29 H :	0.153302
4 H :	0.134846	17 H :	0.127533	30 C :	-0.210159
5 C :	-0.322938	18 H :	0.142563	31 C :	-0.248218
6 O :	0.225581	19 C :	-0.239638	32 H :	0.142164
7 S :	0.396721	20 H :	0.146861	33 H :	0.128155
8 N :	0.168276	21 H :	0.133742	34 H :	0.143469
9 C :	-0.589846	22 H :	0.139506	35 C :	-0.237128
10 N :	0.157974	23 S :	0.450328	36 H :	0.149248
11 C :	-0.240767	24 N :	0.170361	37 H :	0.133943
12 C :	-0.131087	25 C :	-0.546614	38 H :	0.139539

Table S16. Löwdin charges of atoms in **W2**.

0 W :	0.281600	13 C :	-0.217263	26 C :	-0.221685
1 C :	-0.234538	14 C :	-0.249240	27 C :	-0.113391
2 H :	0.131349	15 H :	0.140784	28 H :	0.154194
3 C :	-0.238852	16 H :	0.127881	29 C :	-0.204272
4 H :	0.132077	17 H :	0.142874	30 C :	-0.246951
5 O :	-0.264566	18 C :	-0.241310	31 H :	0.142974
6 S :	0.366782	19 H :	0.148497	32 H :	0.128693
7 N :	0.160266	20 H :	0.131380	33 H :	0.143817
8 C :	-0.600627	21 H :	0.146109	34 C :	-0.235478
9 N :	0.154684	22 S :	0.447775	35 H :	0.149264
10 C :	-0.238236	23 N :	0.160944	36 H :	0.134603
11 C :	-0.129319	24 C :	-0.553931	37 H :	0.145842
12 H :	0.153043	25 N :	0.164229		

References

(1) Vidovič, C.; Peschel, L. M.; Buchsteiner, M.; Belaj, F.; Mösch-Zanetti, N. C. Structural Mimics of Acetylene Hydratase: Tungsten Complexes Capable of Intramolecular Nucleophilic Attack on Acetylene. *Chem. Eur. J.* **2019**, *25* (63), 14267–14272. DOI: 10.1002/chem.201903264.

(2) Baker, P. K.; Fraser, S. G.; Keys, E. M. The synthesis and spectral properties of some highly reactive new seven-coordinate molybdenum(II) and tungsten(II) bisacetonitrile dihalogenotricarbonyl complexes. *J. Organomet. Chem.* **1986**, *309* (3), 319–321. DOI: 10.1016/S0022-328X(00)99633-5.

(3) Chen, X.; Qu, J.-Q. Three Ternary Rare Earth(III) Complexes Based on 3-[(4,6-Dimethyl-2-pyrimidinyl)thio]-propanoic Acid and 1,10-Phenanthroline: Synthesis, Crystal Structure and Antioxidant Activity. *Anorg. All. Chem.* **2015**, *641* (7), 1301–1306. DOI: 10.1002/zaac.201500018.

(4) Bondi, R.; Ćorović, M. Z.; Buchsteiner, M.; Vidovič, C.; Belaj, F.; Mösch-Zanetti, N. C. The Effect of Pyridine-2-thiolate Ligands on the Reactivity of Tungsten Complexes toward Oxidation and Acetylene Insertion. *Organometallics* **2021**, *40* (21), 3591–3598. DOI: 10.1021/acs.organomet.1c00472.

(5) Rodygin, K. S.; Ananikov, V. P. An efficient metal-free pathway to vinyl thioesters with calcium carbide as the acetylene source. *Green Chem.* **2016**, *18* (2), 482–486. DOI: 10.1039/C5GC01552A.

(6) Ehweiner, M. A.; Wiedemaier, F.; Belaj, F.; Mösch-Zanetti, N. C. Oxygen Atom Transfer Reactivity of Molybdenum(VI) Complexes Employing Pyrimidine- and Pyridine-2-thiolate Ligands. *Inorg. Chem.* **2020**, *59* (19), 14577–14593. DOI: 10.1021/acs.inorgchem.0c02412.

(7) Ghorbani-Choghamarani, A.; Tahmasbi, B.; Arghand, F.; Faryadi, S. Nickel Schiff-base complexes immobilized on boehmite nanoparticles and their application in the oxidation of sulfides and oxidative coupling of thiols as novel and reusable nano organometal catalysts. *RSC Adv.* **2015**, *5* (112), 92174–92183. DOI: 10.1039/C5RA14974F.

(8) Ehweiner, M. A.; Belaj, F.; Kirchner, K.; Mösch-Zanetti, N. C. Synthesis and Reactivity of a Bioinspired Molybdenum(IV) Acetylene Complex. *Organometallics* **2021**, *40* (15), 2576–2583. DOI: 10.1021/acs.organomet.1c00289.

(9) Afonin, A. V.; Ushakov, I. A.; Kuznetsova, S. Y.; Andriyankova, L. V. Influence of the C—H· · ·N intramolecular interaction on the spatial structures and ^{1}H and ^{13}C NMR

parameters of heteroaryl vinyl ethers and sulfides. *Magn. Reson. Chem.* **2003**, *41* (8), 557–566. DOI: 10.1002/mrc.1215.

(10) Sheldrick, G. M. A short history of SHELX. *Acta Cryst.* **2008**, *A64*, 112–122. DOI: 10.1107/S0108767307043930.

(11) Sheldrick, G. M. Crystal structure refinement with SHELXL. *Acta Cryst.* **2015**, *C71*, 3–8. DOI: 10.1107/S2053229614024218.

(12) CrysalisPro. *Rigaku Oxford Diffraction*, 2024.

(13) Sheldrick, G. M. SHELXT - integrated space-group and crystal-structure determination. *Acta Cryst.* **2015**, *A71* (Pt 1), 3–8. DOI: 10.1107/S2053273314026370.

(14) Dolomanov, O. V.; Bourhis, L. J.; Gildea, R. J.; Howard, J. A. K.; Puschmann, H. OLEX2 : a complete structure solution, refinement and analysis program. *J. Appl. Cryst.* **2009**, *42* (2), 339–341. DOI: 10.1107/S0021889808042726.

(15) Drosou, M.; Wehrung, I.; Pantazis, D. A.; Orio, M. Accurate Calculation of Electron Paramagnetic Resonance Parameters for Molybdenum Compounds. *Chemphyschem : a European journal of chemical physics and physical chemistry* **2025**, *26* (22), e202500317. DOI: 10.1002/cphc.202500317.

(16) Grimaldi, S.; Biaso, F.; Burlat, B.; Guigliarelli, B. CHAPTER 3. Electron Paramagnetic Resonance Studies of Molybdenum Enzymes. In *Molybdenum and Tungsten Enzymes*; Hille, R., Schulzke, C., Kirk, M. L., Eds.; Metallobiology; Royal Society of Chemistry, 2016; pp 68–120. DOI: 10.1039/9781782628842-00068.

(17) Zanello, P.; Connelly, N. G. *Inorganic Electrochemistry*:*Theory, Practice and Application*; Royal Society of Chemistry, 2003.
